# Selection bias and subject refusal in a cluster-randomized controlled trial

**DOI:** 10.1186/s12874-017-0368-7

**Published:** 2017-07-10

**Authors:** Rochelle Yang, Barry L. Carter, Tyler H. Gums, Brian M. Gryzlak, Yinghui Xu, Barcey T. Levy

**Affiliations:** 1 Department of Pharmacy Practice & Science, College of Pharmacy, University of Iowa, Iowa City, IA 52242 USA; 2 Department of Family Medicine, Roy J. and Lucille A. Carver College of Medicine, University of Iowa, Iowa City, IA USA; 3 Department of Health Outcomes and Pharmacy Practice, University of Texas, Austin, TX USA; 4 Department of Epidemiology, College of Public Health, University of Iowa, Iowa City, IA USA

**Keywords:** Selection bias, Non participation bias, Cluster randomized trial

## Abstract

**Background:**

Selection bias and non-participation bias are major methodological concerns which impact external validity. Cluster-randomized controlled trials are especially prone to selection bias as it is impractical to blind clusters to their allocation into intervention or control. This study assessed the impact of selection bias in a large cluster-randomized controlled trial.

**Methods:**

The Improved Cardiovascular Risk Reduction to Enhance Rural Primary Care (ICARE) study examined the impact of a remote pharmacist-led intervention in twelve medical offices. To assess eligibility, a standardized form containing patient demographics and medical information was completed for each screened patient. Eligible patients were approached by the study coordinator for recruitment. Both the study coordinator and the patient were aware of the site’s allocation prior to consent. Patients who consented or declined to participate were compared across control and intervention arms for differing characteristics. Statistical significance was determined using a two-tailed, equal variance t-test and a chi-square test with adjusted Bonferroni *p*-values. Results were adjusted for random cluster variation.

**Results:**

There were 2749 completed screening forms returned to research staff with 461 subjects who had either consented or declined participation. Patients with poorly controlled diabetes were found to be significantly more likely to decline participation in intervention sites compared to those in control sites. A higher mean diastolic blood pressure was seen in patients with uncontrolled hypertension who declined in the control sites compared to those who declined in the intervention sites. However, these findings were no longer significant after adjustment for random variation among the sites. After this adjustment, females were now found to be significantly more likely to consent than males (odds ratio = 1.41; 95% confidence interval = 1.03, 1.92).

**Conclusions:**

Though there appeared to be a higher consent rate for females than for males, the overall impact of potential selection bias and refusal to participate was minimal. Without rigorous methodology, selection bias may be a threat to external validity in cluster-randomized trials.

**Trial registration:**

NCT01983813. Date of registration: Oct. 28, 2013.

**Electronic supplementary material:**

The online version of this article (doi:10.1186/s12874-017-0368-7) contains supplementary material, which is available to authorized users.

## Background

Selection bias is a major methodological concern for researchers. Bias can be introduced into a study through errors in subject identification or factors related to subject participation [1]. The result is a study population that may be unrepresentative of the total target population eligible for the study, which compromises external validity. In a cluster-randomized controlled trial, randomization occurs at the cluster, or group level, rather than at the individual level. This type of trial design is increasingly used in health services research to reduce the probability of experimental contamination, such as in studies examining effects of new clinical guidelines or health education [2–6]. Cluster-randomized controlled trials are often susceptible to selection bias because many of these studies cannot effectively blind study recruiters or potential subjects to the allocation of their cluster prior to consent [7, 8]. There may be characteristics of the clusters that, although randomly allocated, affect the study results. For example, subjects with a certain characteristic may be less likely to enroll in a study if they are aware of being assigned to the control group and there may be differences between study groups with regards to the aforementioned characteristic.

There are several accepted ways to mitigate the risk of selection bias in cluster-randomized studies [7, 9–11]. One strategy is to identify and consent subjects prior to cluster randomization to ensure that recruitment will not be affected by subject knowledge of his or her allocation. This approach is usually not logistically practical. Another method is to have a third party who is blinded to cluster allocation carry out recruitment. However, this method also poses challenges to consent, may require multiple rounds of consenting, and runs the risk of participants opting out of the study if they do not end up in their desired intervention arm. It may not be practical, or possible, to employ either of these methods in most studies.

Differences in recruitment between control and intervention groups have been documented in many cluster-randomized controlled studies [12–14]. In a review analyzing 36 cluster-randomized trials that were published in three leading medical journals between 1997 and 2002, 14 (39%) showed susceptibility to bias at the individual level due to differing consent rates or a differing patient characteristic between control and intervention groups [11]. Likewise, in a systematic review of 34 cluster-randomized controlled trials published between 2004 and 2005, a quarter of them were potentially biased due to the methodology for subject recruitment, leading to selection bias with differential consent rates between control and intervention groups [4]. In the Diabetes Education and Self-Management for Ongoing and Newly Diagnosed (DESMOND) study of a diabetes intervention [15], possible recruitment bias resulted in a baseline population with a significantly higher mean HbA1c in the intervention group than in the control group (8.3% vs 7.9%; *p* < 0.05) as well as a significantly higher percentage of intervention group participants on oral hypoglycemic medications compared to controls (17% vs 12%; *p* < 0.05).

The Improved Cardiovascular Risk Reduction to Enhance Rural Primary Care (ICARE) study was a prospective cluster-randomized controlled trial that compared a centralized clinical pharmacist care management model to usual care in patients with cardiovascular disease risk [16]. A total of 302 subjects were recruited from 12 different rural primary care offices across Iowa. Half of the sites were randomly assigned to the intervention arm where centralized clinical pharmacists worked with intervention site patients to improve their cardiovascular conditions, manage medication regimens, and promote preventative care, while patients from sites in the control arm received usual care. Because randomization of the offices occurred prior to subject identification, and potential subjects were aware of site allocation at the time of recruitment, the study was potentially affected by selection bias. The patient’s decision to consent may have been influenced by the allocation of his or her office to control or intervention arm based on their desire to work with a study pharmacist. In addition, the staff at the sites may have preferentially recruited participants based on allocation. For example, clinics in the intervention arm, aware that a clinical pharmacist would be helping to manage care, may have enrolled more complicated patients than their control group counterparts.

The purpose of this study was to assess the impact of possible selection bias in subject participation by comparing the subjects who actively refused consent (“declines”) in the control vs. intervention clusters and comparing baseline characteristics of those who enrolled in the study to those who declined within each of the study arms.

The hypotheses for this study (stated as the a priori null hypotheses) are that there will be no significant difference in patient characteristics in:

1. Subjects who declined to participate in the intervention group compared with those who declined in the control group.
2. Subjects who were enrolled into the intervention group and those who are enrolled into the control group.
3. Subjects who declined to participate in the control group compared with those who were enrolled into the control group.
4. Subjects who declined to participate in the intervention group compared with those who were enrolled into the intervention group.

## Methods

### Screening

The study background and methods have been published [16]. Briefly, a study coordinator, usually the office manager or a nurse, was designated at each office and trained by the research team in study procedures. Office staff were asked to generate lists of patients with one or more ICARE study inclusion criterion using their practice’s electronic medical record. Eligibility criteria for the study were based on a two-step inclusion criteria process [16]:

First, English speaking males or females at least 50 years of age, seen at least once in the clinic or practice in the previous 24 months with a history of at least one of the following chronic medical conditions and associated uncontrolled risk factors identified:

- Diabetes, with HbA1c ≥7.5% and/or
- Hypertension, with

i) Systolic Blood Pressure (SBP) ≥150 mmHg or Diastolic Blood Pressure (DBP) ≥90 mmHg for patients with uncomplicated hypertension OR
ii) SBP ≥140 mmHg or DBP ≥90 mmHg for patients with diabetes or chronic kidney disease.
  and/or

c. Hyperlipidemia, with
  - LDL >110 mg/dL for patients with peripheral artery disease, coronary artery disease, history of stroke, history of transient ischemic attack, or diabetes OR
  - LDL >140 mg/dL in all other subjects

Study coordinators were instructed to record only the most recent laboratory value found in the patient’s medical record from within the past 24 months. The inclusion criteria did not specify whether a patient had either Type I or Type II diabetes. If the patient met at least one of the above criteria for uncontrolled diabetes, hyperlipidemia, or hypertension, further eligibility criteria were assessed. To meet final eligibility, the subject had to have a history of at least three or more conditions from the above list and/or any of the following: coronary artery disease, myocardial infarction, stroke, transient ischemic attack, atrial fibrillation, peripheral vascular disease, claudication, carotid artery disease, be a current smoker, or have a diagnosis of obesity (body mass index ≥30). Subjects were excluded if they had: cancer with a life expectancy less than 24 months, pregnancy, diagnosis of primary pulmonary hypertension, inability to give informed consent, nursing home residence or diagnosis of dementia, no telephone access or a hearing impairment preventing them from telephone use, refusal to consider attempting to using the internet either at home or at a community location to access the study’s online communication tool (Iowa Personal Health Record) between pharmacist and subject, inability to use the provided Omron blood pressure cuff over the arm for any reason, or plans to move from the area or transfer care to a different clinic in the next 12 months. The ICARE eligibility criteria for uncontrolled conditions were developed in 2012 and were higher than those recommended by clinical guidelines [17–19] to ensure a patient truly had an uncontrolled, rather than borderline, condition.

Specific criteria used to generate patient lists varied based on the sites’ ability to program more or less complex queries. A typical list might include patient age greater than or equal to 50 years, at least one visit in the previous 24 months, and a diagnosis of hypertension, hypercholesterolemia, or diabetes. The study coordinators were instructed to use the generated lists and complete a screening form for each patient screened (see Additional file 1). A unique, site-specific numeric ID was attached to each screening form. Completed forms were returned to the research team for entry into a database. Patients who met eligibility criteria were contacted by the study coordinator and invited to participate in the study.

### Recruitment

Study coordinators could employ multiple recruitment approaches once an eligible patient was identified (see Additional file 2). First, the patient could be mailed a letter with information regarding the study along with a return postcard to indicate interest in participation. Second, the patient could be contacted via phone by the study coordinator using an approved phone script to briefly explain the study with the option to send additional information through the mail. Third, a patient could be approached by the study coordinator in-person at his or her next office visit. Sites also had the option of placing study brochures in their waiting and exam rooms. If the study coordinator was not able to contact the patient after a maximum of three attempts using any method, the patient was deemed “unable to be reached.”

In all recruitment scenarios, the patient was made aware of the designation of the office as intervention or control and whether he or she would be receiving the pharmacist intervention or usual care prior to enrollment. All potential subjects were also informed of the required research clinic visits at baseline and at 12 months, standardized blood pressure measurements, venipuncture for HbA1c and LDL, patient questionnaires, and subject compensation ($75 for each of two study visits; a total of $150). Patients expressing interest were asked additional screening questions. Those who fully met eligibility criteria were scheduled for an initial study visit where written informed consent was obtained. The study was approved by the University of Iowa Institutional Review Board.

### Data collection and quality assurance

Screening forms were completed by the study coordinators and mailed or faxed back to the research team. Outcomes for each screened patient, including “patient enrolled”, “patient ineligible”, “patient declined”, “patient unable to be reached”, or “other outcome” (with explanation) were indicated on every form. Returned forms were entered by research staff into a customized database hosted by an in-house web portal. Quality assurance measures were then conducted by the research team. Reports were run to check for duplicate entries of the same screening form, which were then reconciled. Entries with values lying outside of reasonable range for age, dates, HbA1c, LDL cholesterol, or blood pressure were checked against the physical screening form copies to correct data entry errors.

A study monitor from the university research team obtained access to the electronic medical records at all sites. The study monitor selected a review date that was after the first 3–5 participants had been enrolled at a given site, or approximately three months after the first eligible participant had been enrolled from that site, whichever came first. The intent for this approach was for the study monitor to catch recurring errors by the study coordinator early on to prevent future errors. The study monitor reviewed forms for all subjects who had been enrolled up until the review date. Medical charts of these patients were reviewed for accuracy in screening form completion, as well as to verify that the patient had indeed met all eligibility criteria for the study. Discrepancies were communicated to the study coordinator, who could either appeal the decision or accept the changes. Once the necessary action was agreed upon, the entries were corrected in the database.

Entered forms were also checked against their physical copies for cases where the patient declined, but the outcome was entered incorrectly in the database as “patient unable to be reached,” “other outcome,” or the outcome was left blank. In addition, research staff attempted to confirm the appropriate outcome with the study coordinators for returned screening forms without an outcome selected. A second report was run that examined screening forms in which the “patient declined” outcome had been selected, but the patient did not actually meet study eligibility criteria. Again, data fields were compared to the physical copies and data entry errors were corrected.

### Analysis

Patient characteristics were selected for analysis based on the content of the screening forms. The primary screening variables of age, gender, status of uncontrolled diabetes, uncontrolled hyperlipidemia, and uncontrolled hypertension, and latest HbA1c, LDL, and blood pressure recorded in the medical chart were selected for analysis as these received the highest completion rates among the screening forms. Smoking status, was also selected for analysis based on literature suggesting a correlation between smoking and willingness to participate in health behavioral intervention trials [20, 21]. Study coordinators were instructed not to record values on the screening form unless they were above these threshold criteria (i.e. if a patient’s most recent HbA1c was below 7.5%, the field for HbA1c was left blank.)

Each of the patient characteristics were tested between the groups as stated in the four hypotheses. Statistical significance was determined using a two-tailed, equal variance t-test for means and a chi-square test for percentage values. Since we performed multiple comparisons on the same data, which may increase the probability of false-positives, we calculated the adjusted Bonferroni *p*-values using SAS proc. MULTTEST. In order to control the inter-cluster correlation, we developed the generalized estimating equation (GEE) or MIXED model for the analysis. If the dependent variable was a dummy variable, SAS proc. GENMOD was used, and the binomial distribution with the logit link were fit in the model. A repeated statement was used to accommodate the correlations of patients within clinics, and the exchangeable correlation matrix was specified as the working correlation matrix. If the dependent variable was continuous and with approximate normal distribution, SAS proc. MIXED with random intercept statement was used. The interaction between two variables: the study group (intervention vs. control) and whether patient consented (consented vs. declined) was tested in the model. The analyses were performed using SAS version 9.4 (SAS, Inc., Cary, North Carolina, USA) on Windows 7 Enterprise.

## Results

**Fig. 1:**
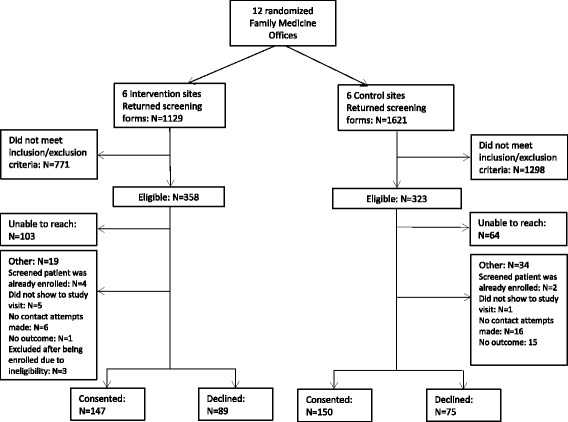
Subjects screen and enrolled into the trial

Of the 2749 total screening forms returned, 358 from the intervention sites and 323 from the control sites met eligibility criteria (Fig. 1). There were 147 patients consented in the intervention group while 89 patients declined. Control group offices enrolled 150 patients, and 75 patients declined. Five subjects who consented in the study were not included in this analysis because they did not have screening form data returned. Both control and intervention groups saw a higher consent rate compared to decline rate, and decline rates between these groups were similar (24.9% intervention vs 23.2% control).

None of the patient characteristics were significantly different across the four comparison groups with the exception of two. In patients with uncontrolled diabetes (having a HbA1c ≥7.5%), where there was a higher decline rate (70.8%) in the intervention sites compared to the decline rate in the control sites (46.7%; *p* = 0.0017; adjusted Bonferroni *p* = 0.0068) (Table 1). There was a higher diastolic blood pressure seen in patients with uncontrolled hypertension who declined in the control sites (86.6 mmHg) compared to patients who declined in the intervention sites (79.7 mmHg; *p* = 0.012; adjusted Bonferroni *p* = 0.048) (Table 1). Though not statistically significant, patients with uncontrolled diabetes within control sites trended towards a higher rate of consent over decline to participate (63.3% consented vs. 46.7% declined; adjusted Bonferroni *p* = 0.068). Intervention sites trended towards having a higher rate of patients with uncontrolled hypertension who consented to the study (59.9%) compared to control sites (48.0%), but this also did not reach statistical significance after adjustment for Bonferroni (*p* = 0.08). There were no significant differences with regards to age or smoking status. Hyperlipidemia also did not appear to play a role in a patient’s decision to participate.

**Table 1:** Comparison of patient characteristics^a^

Characteristic	Intervention Sites			Control Sites			Enrolled in Intervention vs. Control	Declined in Intervention vs. Control	genmod model or mixed model
	A:	B:	C:	D:	E:	F:	H:	I:	G:
	Consented (*n* = 147)	Declined (*n* = 89)	*P*-value between A and B	Consented (*n* = 150)	Declined (*n* = 75)	*P*-value between D and E	*P*-value between A and D	*P*-value between B and E	Study group and consent as independent variables
Mean Age (±SD)	63.2 (±9.0) (*n* = 147)	65.2 (±8.9) ^b^ (*n* = 86)	0.099	63.8 (±8.0) (*n* = 150)	64.1 (±8.6) ^d^ (*n* = 73)	0.801	0.547	0.424	0.8963 0.1013
% Female	44.2% (65/147)	37.9% ^c^ (33/87)	0.346	53.3% (80/150)	42.7% (32/75)	0.131	0.116	0.540	0.1855 **0.0338**
% with uncontrolled diabetes	61.9% (91/147)	70.8% (63/89)	0.165	63.3% (95/150)	46.7% (35/75)	0.017	0.799	**0.0017**	0.6786 0.3105
Mean A1c	8.7 (±1.1) (*n* = 91)	9.1 (±1.5) (*n* = 63)	0.154	8.8 (±1.1) (*n* = 95)	8.9 (±1.3) (n = 35)	0.806	0.669	0.523	0.9187 0.1733
% with uncontrolled hyperlipidemia	35.4% (52/147)	40.5% (36/89)	0.435	48.7% (73/150)	42.7% (32/75)	0.395	0.020	0.774	0.1139 0.8094
Mean LDL (±SD)	145.0 (±35.2) (*n* = 52)	140.4 (±26.1) (*n* = 36)	0.502	144.8 (±26.9) (*n* = 73)	147.3 (±33.4) (*n* = 32)	0.686	0.970	0.342	0.7408 0.7349
% with uncontrolled hypertension	59.9% (88/147)	52.8% (47/89)	0.288	48.0% (72/150)	56.0% (42/75)	0.258	0.040	0.683	0.3855 0.8320
Mean Systolic BP (±SD)	152.1 (±15.9) (*n* = 88)	151.8 (±15.5) (*n* = 47)	0.918	149.0 (±10.7) (*n* = 72)	149.6 (±13.6) (*n* = 42)	0.818	0.146	0.470	0.3601 0.6283
Mean Diastolic BP (±SD)	83.4 (±11.8) (*n* = 88)	79.7 (±12.3) (*n* = 47)	0.084	84.2 (±11.1) (*n* = 72)	86.6 (±13.1) (*n* = 42)	0.297	0.689	**0.012**	0.2396 0.3901
% Current Smoker	29.3% (43/147)	20.2% (18/89)	0.125	22.7% (34/150)	25.3% (19/75)	0.657	0.195	0.436	0.4250 0.0805

^a^Mean values are shown as mean (± standard deviation). Percentage values are shown as percent of patients with characteristic out of total number of patients in subgroup

^b^Missing age information for three subjects

^c^Missing gender information for two subjects

^d^Missing age information for two subjects

Bolded value for females is the only significant value when controlling for random clinic effects

After adjusting for random cluster effects in the Genmod or Mixed model, however, there was no significant interaction of study group (control or intervention) and whether a patient consented or declined for uncontrolled diabetes or for uncontrolled diastolic blood pressure, nor was there a significant difference between the study groups (control and intervention) or between consented and declined for these two characteristics (*p* = 0.3105 and *p* = 0.3901 respectively). Thus, the initial differences appeared to be chance findings due to random variations among the sites. Gender was significant when the model was adjusted for random cluster effects (*p* = 0.0338, Table 1). Females were significantly more likely to consent to the study than males (odds ratio = 1.41;95% confidence interval = 1.03,1.92). However, because more males than females were approached for study recruitment, there was still a greater number of males who consented to the study overall. A distribution of each characteristic in Table 1 by individual sites, distinguished by control and intervention, are provided in Additional file 3.

## Discussion

This is the first study, to our knowledge, that evaluated the impact of selection bias in a cluster-randomized controlled trial using data from both participating subjects and those who declined participation. A search of Medline from years 2000 to 2016, using the terms “cluster-randomized trial” and “selection bias” resulted in 14 articles. After reviewing abstracts of these articles, none reported data on those who declined participation. Overall, selection bias in ICARE appeared to be minimal with no significant differences among the characteristics tested between intervention and control groups after adjusting for random cluster effects with the exception of gender. Women appeared to have been more likely to consent to the study than men across both control and intervention study groups.

It does not appear that sites selected certain patients for participation based on their knowledge of the allocation into intervention or control group. For example, if intervention sites had enrolled more poorly-controlled patients into the study knowing that clinical pharmacists would be helping with their care, we would have expected to see higher mean HbA1c, blood pressure, or LDL values for patients enrolled in the intervention sites compared to control sites. However, this was not the case. Additionally, it does not appear that a particular uncontrolled disease state found on the screening form influenced a patient’s decision to consent or decline participation. There was no indication of imbalances between intervention and control groups for patients with diabetes that was more difficult to manage [22–24] and thus the study’s external validity is maintained among this patient population.

Interestingly, there were no differences observed among the percentage of current smokers who consented or declined participation between control and intervention groups. This is in contrast to other health behavioral and preventative care intervention studies in the literature, which historically suggest a higher rate of non-participation and drop-out among smokers [20, 21, 25, 26]. In this case, we would have expected to see a higher decline rate compared to consent rate in the intervention sites. However, results were the opposite. In the intervention arm, there was a similar consent rate among smokers compared to refusals (29% vs. 20%; *p* = 0.125). This suggests that neither the intervention nor the recruitment approach caused bias in the recruitment of patients who smoked.

There are several limitations in this study related to the site screening and recruitment process. While we provided instructions for how to generate the patient screening lists, the site personnel decided for themselves how to best generate lists. We cannot exclude the possibility that study coordinators may have relied on their knowledge of a patient’s clinical characteristics, past experiences with the patient, future scheduled clinic visits, or input from clinic providers when deciding whether to approach the patient for recruitment. In addition, because the screening forms were completed by study coordinators and not by central research staff, we could not monitor all forms to ensure they were correctly and completely filled out. Although we instructed site coordinators not to record screening form values that were below eligibility thresholds, there is no way to determine whether missing data were for normal values, or if the site coordinator simply could not find or overlooked specific values. We also cannot exclude the possibility that study coordinators did not send all of the screening forms back to the research team, potentially leading to missing data. In addition, the values on the screening form were sometimes recorded several months prior to the patient’s baseline visit. This, along with the fact that values below eligibility criteria were not recorded on the screening form, means there may have been variation between results from the screening form data and results from information collected at the baseline visit.

Another limitation related to the study monitoring process. The protocol called for a review of screening forms after the first 3–5 patients had been enrolled at that site or three months following the enrollment date of the first patient enrolled, whichever came earlier. However, due to time constraints and delays in gaining electronic medical record access, the study coordinator was not able to conduct the review until more than 3–5 patients had already been enrolled for many of the sites. The number of participants whose forms were ultimately verified by the study monitor at each site ranged from four to fourteen. We cannot exclude the possibility that sites that had fewer forms reviewed and verified by the study monitor may have had a greater number of errors in their screening forms. We also cannot exclude the possibility that study coordinators of these sites may have relaxed their attentiveness to screening form completion if they knew that future enrolled patients would no longer be subject to review by the study monitor.

Furthermore, the standardized screening form was not implemented until shortly after study enrollment began. Prior to the forms, sites kept track of their screened patients on simple spreadsheet logs that did not mandate documentation of the outcome or of specific values such as HbA1c, LDL, and blood pressure. Thus, we were not able to use information from these forms in our analysis. As a consequence, some patient refusal data may have been missed. However, because patients screened using these spreadsheet logs constituted less than 5% of the total patients screened and there were an equal number of sites from control and intervention arms that used these earlier logs, study results were unlikely to have been impacted in the present analysis.

We were unable to include race as a patient characteristic due to the low number of minorities in this trial. Previous literature shows discrepancies in clinical trial participation among different races, particularly lower rates among African Americans [27, 28]. Although race and ethnicity were primary screening items on the screening form, only 50 screening forms recorded a race or ethnicity other than non-Hispanic white, making up less than 2% of total screened. This is mostly likely due to the demographics of rural Iowa (92% non-Hispanic, white) [29] where most of the participating clinics were located. In addition, previous literature shows differential rates of response for various methods of recruitment such as by phone, by mail, or in-person [30–32]. We were unable to draw conclusions from our data due to the small sample size of patients who declined via means other than the telephone. There were two patients who declined by mail (both in the intervention sites) and 19 patients who declined in-person (eight in the intervention sites and 11 in the control sites). In addition, we did not collect data on the method of recruitment used for patients who chose to enroll in the study. Thus, we cannot rule out differences in recruitment method among the 12 sites or assess the impact of such possible differences on participant refusal. Regardless of these limitations, the lack of any differences between groups suggested that systematic recruitment bias was unlikely.

### Lessons learned and suggestions

There are several ways in which our recruitment strategies could be improved upon for future cluster-randomized health science research studies to minimize potential recruitment bias. Characteristics and data on people who decline to participate in research trials is often limited and not well documented or reported [33, 34]. Because we implemented a standardized screening form, we were able to collect certain characteristics of patients not enrolled into the study, thus allowing us to keep track of the rates of study recruitment, declines and examine any differences in participation related to selection bias.

The instructions on how to generate screening lists of potentially eligible patients should be as clear and specific as possible for research personnel to follow. For example, each site could have been instructed to generate three different lists of all patients at least 50 years of age with at least one clinic visit in the last 24 months and having 1) HbA1c of ≥7.5% 2) LDL >140 mg/dL, and 3) SPB ≥150 and screen each patient from those three lists. This may help reduce subject variation between the clusters, especially if subjects are randomly selected from all lists. The implementation of a standardized screening list generation process will be dependent upon the technical abilities of each site’s medical record system to process more specific queries. However, at the very least, there should be a formal documentation system required by each site to report to central research staff the queries from which their screening lists were generated, including additional sources of patients for screening. These sources would include patients suggested to the study coordinator by a site provider. In addition, the method of recruitment should be collected for all patients, both those who consent to the study and those who refuse participation. This information will help study investigators determine if sites may be using very different methods of recruitment, thus introducing selection bias into the study. In addition, the reasons for participant refusal can also be collected for further information on patient groups that may not be represented in subject population as well as to improve recruitment strategies.

A standardized script should be drafted for study recruiters to ask the patient following a refusal, with options such as lack of perceived benefit, lack of time, lack of interest in the intervention, and more. Lastly, the standardized screening form used by the study recruiters should be as comprehensive as possible when collecting information, leaving as little as possible up to the recruiter’s discretion to record. For example, in our screening form, we only asked study coordinators to record the most recent laboratory value on the form if it was high enough to meet inclusion criteria for poor control for that particular disease state. However, it would have perhaps been better to record the most recent laboratory value regardless of whether it met inclusion criteria. Thus, we would have been able to differentiate between a patient truly not meeting uncontrolled criteria for that disease state or the study coordinator simply forgetting to record a lab value that may have been considered uncontrolled, resulting in missing data. Unfortunately, all of these procedures would take valuable time that many research personnel would find burdensome. In addition, subjects refusing to participate may not want to take the time to answer additional questions. Investigators need to balance the need to minimize selection bias without adding significant burdens to patients or study personnel.

## Conclusion

The impact of selection bias and subject refusal was minimal in the ICARE study. No significant differences were found when comparing characteristics between intervention and control groups for patients who either enrolled in the study or declined to participate in the study after adjusting for random cluster effects with the exception of gender. Because cluster-randomized controlled trials often require the randomization to precede subject recruitment, these trials are especially susceptible to selection bias that may be introduced by both the site recruiter and the participant. Selection bias is a potential, major threat to external validity in the cluster-randomized controlled trial design without rigorous methodology in screening and recruitment.

## Additional files

Additional file 1: Sample screening form. The standardized form that study coordinators completed for each patient screened for eligibility. (PDF 213 kb)

Additional file 2: ICARE study protocol. A document describing the full study protocol, including screening and recruitment procedures. (PDF 6937 kb)

Additional file 3: Distribution of patient characteristics and medical conditions among patients who consented and patients who declined by individual study site. (DOCX 48 kb)

## Abbreviations

DBP: Diastolic blood pressure
HbA1c: Hemoglobin A1c
ICARE: Improved cardiovascular risk reduction to enhance rural primary care
LDL: Low-density lipoprotein
mg/dL: milligrams per deciliter
mmHg: millimeters of mercury
SBP: Systolic blood pressure
