# Supplementary material for: Selection bias and subject refusal in a cluster-randomized controlled trial
Source: BMC Med Res Methodol. 2017 Jul 10;17:94. doi: 10.1186/s12874-017-0368-7 (PMC5504663; doi:10.1186/s12874-017-0368-7)
Supplement: Supplementary file 1 — Sample screening form. The standardized form that study coordinators completed for each patient screened for eligibility. (PDF 213 kb) [file 12874_2017_368_MOESM1_ESM.pdf]

Patient Name:	MRN:	Phone number:

Do not send information in the row above to the University of Iowa. *Please copy this form and redact or cut out this info before sending.*

SCREENING LOG AND VERIFICATION OF INCLUSION AND EXCLUSION CRITERIA

Screening Number: 601

INSTRUCTIONS

* = *Required for all screened patients*

- Please record screening data below for any patient whose chart you have accessed for the ICARE study.
- Patients that you identify as potentially eligible for the study should be sent a recruitment letter/consent/brochure.
- If you have questions on this form contact Brian at 319-353-3857 or brian-gryzlak@uiowa.edu

SAMPLE - DO NOT USE

Screening Number: 601		*Outcome:
*Gender: ❒ Male ❒ Female	*Race (Check all that apply):<br>❒ White<br>❒ Black/African-American.<br>❒ Asian<br>❒ American Indian/Alaska Native<br>❒ Native Hawaiian/Other Pacific Islander	❒ Patient enrolled in study<br>❒ Patient ineligible for study<br>❒ Patient declined via letter<br>❒ Patient declined via phone<br>❒ Patient declined in person<br>❒ Unable to reach via mail<br>❒ Unable to reach via phone<br>❒ Other outcome:
*Ethnicity: ❒ Hispanic ❒ Not Hispanic		
Date letter mailed: __ __/__ __/__ __ __ __ ❒ n/a		
Date letter returned: __ __/__ __/__ __ __ __ ❒ n/a		

Phone contact dates and notes (up to 3 attempts):

Section A: Demographic Criteria

1.	Has the patient been seen in your clinic or practice at least once in the past 24 months?	❒ YES ➔ **Continue to #2**<br>❒ NO ➔ **STOP – not eligible**
2.	Is the patient an English-speaking male or female?	❒ YES ➔ **Continue to #3**<br>❒ NO ➔ **STOP - not eligible**
3.	Age of patient in years:	AGE:_______ **IF >= 50, go to #4a. If less than 50, STOP – not eligible.**

Section B: Risk Factors (search back 24 months in the medical record)

Question	Response		Meets Criteria?
**4a**. Has a diagnosis of diabetes? *(ICD9 Code 250)*	❒ YES ➔ Continue to #4b<br>❒ NO ➔ Skip to #5a	If 4a is YES AND 4b is >= 7.5%, check box at right.	❒
**4b**. Enter most recent Hg A1c from chart **IF** ≥ 7.5%:	Hg A1c:______% Date: __ __ / __ __ / __ __ __ __		
**5a**. Has a diagnosis of high cholesterol? *(ICD9 Code 272)*	❒ YES ➔ Continue to #5b<br>❒ NO ➔ Skip to #6a	If 5a is YES AND (5b is >110mg/dl for patients with PAD, CAD, Stroke, TIA, or Diabetes <u>OR</u> 5b >140mg/dl), check box at right.	❒
**5b**. Enter most recent LDL from chart **IF**:<br>▪ >110mg/dl for patients with PAD, CAD, Stroke, TIA, or Diabetes **<u>OR</u>**<br>▪ >140mg/dl	LDL: ______mg/dl Date: __ __ / __ __ / __ __ __ __		
**6a**. Has a diagnosis of hypertension? *(ICD9 Codes 401, 402, 403, 404, 405)*	❒ YES ➔ Continue to #6b<br>❒ NO ➔ Skip to #7	If 6a is YES AND (6b ≥150 mm Hg SBP or > 90 mm Hg DBP for patients with uncomplicated hypertension <u>OR</u> 6b ≥ 140 mm Hg SBP for patients with diabetes or chronic kidney disease), check box at right.	❒
**6b**. Enter most recent blood pressure from chart **IF**:<br>▪ ≥150 mm Hg SBP or ≥ 90 mm Hg DBP for patients with uncomplicated hypertension **<u>OR</u>**<br>▪ ≥140 mm Hg SBP for patients with diabetes or chronic kidney disease	__________(SBP)<br>__________(DBP) Date: __ __ / __ __ / __ __ __ __		

7. **<u>Section B Total</u>**: In box at the right, add the number of checked boxes above for **Questions 4, 5 and 6.** ➔ ☐

If 1 or more, CONTINUE to Section C. If 0, STOP - not eligible.

**Section C: Cardiovascular Conditions** *(ICD9 Codes)*	**Check if meets inclusion criterion**
**8.** History of coronary artery disease (CAD) *(ICD9 Code 414)*	❑
**9.** History of previous MI (heart attack) *(ICD9 Codes 410, 411, 412)*	❑
**10.** History of stroke *(ICD9 Codes 430, 431, 432, 433, and 434)*	❑
**11.** History of TIA *(ICD9 Code 435)*	❑
**12.** History of atrial fibrillation (A. Fib) *(ICD9 Codes 427.31, 427.3)*	❑
**13.** History of peripheral vascular disease/claudication (PAD) *(ICD9 Codes 440.2, 440.3, 440.4, and 443.9)*	❑
**14.** History of carotid artery disease *(ICD9 Code 433.1)*	❑
**15.** Current smoker *(ICD9 Code 305.1)*	❑
**16.** Diagnosis of obesity (BMI ≥30) *(ICD9 Code 278.0)* Enter most recent BMI >= 30 from chart: _________ Date: __ __ / __ __ / __ __ __ __	❑
**17.** **<u>Section C Total</u>:** In box at the right, add the number of checked boxes for **Questions 8-16➔**	☐
**18.** Add the answers for #7 & #17. **If 3 or more, CONTINUE to # 19. If less than 3, STOP – not eligible ➔**	☐

**Section D: Exclusion Criteria** *(from Medical Record, Direct Observation, OR Self-Report)*	**Check if meets exclusion criterion**
**19.** Inability to give informed consent - *direct observation OK*	❑
**20.** Pregnant *(ICD9 Codes V22, V23, V24)*	❑
**21.** Diagnosis of pulmonary hypertension (*ICD9 Code 416; Note: secondary pulmonary hypertension is OK)*	❑
**22.** Cancer diagnosis with a life expectancy estimated less than 2 years	❑
**23.** Residence in a nursing home or diagnosis of dementia –*Self-report OK for N.H. residence*	❑
**24.** No telephone or have a hearing impairment not allowing them to use a phone – *Direct observation and self-report OK*	❑
**25.** Omron blood pressure cuff cannot be used on patient's arm for any reason – *direct observation OK* (e.g., patient is morbidly obese and requires use of a thigh cuff)	❑
**26.** **<u>Section D total:</u>** In box at the right, add the number of checked boxes for **Questions 19-25➔** **If 0, CONTINUE to Question 27. If 1 or more, STOP– not eligible**	☐
**27.** Subject is potentially eligible based on your chart review and can be sent a recruitment letter / consent / brochure or otherwise considered for enrollment. <u>At the right, enter the date that the chart review for Sections B, C, and D was completed.</u> Then continue to Question 28 in Section E.	Date: __ __ / __ __ / __ __ __ __

**Section E: Exclusion Criteria from Patient Self-Report** *(Ask during your contact to schedule the baseline visit)*	**Check if meets exclusion criterion**
**28.** Refusal to consider attempting to use the internet to access the PHRM	❑
**29.** Patient has plans to move from the area or transfer care to a different clinic in the next 12 months	❑
**30.** **<u>Section E total:</u>** In box at the right, add the number of checked boxes for Questions 28-29➔ **If 0, CONTINUE to Question 31. If 1 or more, STOP– not eligible**	☐

**31. Before the subject signs consent, verify that all are correct:**	❒ ANSWER TO QUESTION 18 IS 3 OR MORE.<br>❒ DATE IN QUESTION 27 IS LESS THAN 6 MONTHS FROM THE DATE THAT THE PATIENT WOULD SIGN THE CONSENT DOCUMENT (#32 BELOW). *If the date in Question 27 is MORE THAN 6 months from the date the consent would be signed, rescreen the patient for eligibility with a new screening log using current medical record data.*<br>❒ PATIENT HAS NONE OF THE EXCLUSION CRITERIA BASED ON MEDICAL RECORD, DIRECT OBSERVATION, OR PATIENT REPORT
**32.** Subject may sign consent. Enter date subject signed:	Date: __ __ / __ __ / __ __ __ __

SAMPLE - DO NOT USE
