# Supplementary material for: Selection bias and subject refusal in a cluster-randomized controlled trial
Source: BMC Med Res Methodol. 2017 Jul 10;17:94. doi: 10.1186/s12874-017-0368-7 (PMC5504663; doi:10.1186/s12874-017-0368-7)
Supplement: Supplementary file 2 — ICARE study protocol. A document describing the full study protocol, including screening and recruitment procedures. (PDF 6937 kb) [file 12874_2017_368_MOESM2_ESM.pdf]

ICARE
Improved Cardiovascular Risk Reduction to Enhance Rural Primary Care

Study Protocol

Date: [02/12/2015]

Version: 1.0

Principal Investigator
Barry L. Carter, Pharm.D., the Patrick E. Keefe Endowed Professor of Pharmacy, College of Pharmacy and Professor, Department of Family Medicine, College of Medicine.

Co-Principal Investigator
Barcey Levy, M.D., Ph.D., the Iowa Academy of Family Physicians Endowed Chair in Rural Medicine and Professor and Associate Head for Research, Department of Family Medicine and Professor, Department of Epidemiology, College of Public Health.

Grant Number: 5R01HL116311-02

Project/Grant Period: 08/08/2013 - 07/31/2018

Clinical Trials Number: NCT01983813

TABLE OF CONTENTS

TABLE OF CONTENTS 2

PROTOCOL APPROVAL PAGE 7

PROTOCOL VERSION AND AMENDMENT TRACKING 8

PROTOCOL SYNOPSIS 9

SITE LEAD PROVIDER STATEMENT 11

ABBREVIATIONS 12

1. INTRODUCTION 14

1.1. Background 14

1.2. Clinical Experience with the Study Intervention 15

1.3. Rationale 15

2. STUDY ORGANIZATION 17

2.1. Personnel 17

2.2. Regulatory and Billing Requirements 18

2.2.1. IRB Oversight 18

2.2.2. Subaward Agreement with the University of Iowa 19

3. OBJECTIVE, AIMS, AND HYPOTHESES 20

3.1. Objective 20

3.2. Aims and Hypotheses 20

4. SUBJECT SELECTION / ELIGIBILITY CRITERIA 21

4.1. Inclusion Criteria 21

4.2. Exclusion Criteria 21

5. STUDY DESIGN 23

5.1. Study Overview **23**

5.2. Site Randomization and Treatment Arms **23**

5.3. Schedule of Measurements **24**

5.4. Recruitment Procedures **24**
5.4.1. Screening Log and Verification of Inclusion and Exclusion Criteria (“Screening Log/Verification Form”) 25
5.4.2. Screening questions 25

6. STUDY VISITS AND DATA COLLECTION 26

6.1. Schedule for Completing Case Report Forms **26**
6.1.1. Pre-Baseline Visit Procedures 27

6.2. Enrollment and Steps for Obtaining Informed Consent **27**

6.3. Blood Pressure Measurement **29**

6.4. Laboratory Specimen Collection **29**

6.5. Subject Orientation to the Iowa Personal Health & Research Management (PHRM) System (Baseline visit only) **29**

6.6. Transfer of CRF Data to the CCC **30**

6.7. Data Entry **30**

6.8. Subject Reimbursement **31**

6.9. Procedures for Measuring Research Blood Pressures **31**
6.9.1. Preparing the Subject 31
6.9.2. Cuff Measurement 32
6.9.3. Cuff Placement 32
6.9.4. Blood Pressure Measurement 32

Figure 1: Handling Hypertensive Urgency **34**

6.10. 12-Month Visit **35**

6.11. Medical Record Data Abstraction at 30 months **35**

7. STUDY OUTCOMES 37

7.1. Primary Outcomes **37**
7.1.1. Adherence to Guideline Advantage Criteria in All Subjects 37

7.2. Secondary Outcomes **37**
7.2.1. Adherence to Guideline Advantage Criteria at 30 months 37
7.2.2. BP Control, Mean BP, LDL Cholesterol, HA1c 37
7.2.3. Measurement of Stages of Change 37
7.2.4. Intensity of Medication Management 37
7.2.5. Medical Home Index 38

7.2.6. Provider attitudes to deliver intervention, barriers and facilitators to implementation 38

8. STATISTICAL ANALYSIS PLAN 39

8.1. Statistical Design 39

8.2. Primary Analysis 39
8.2.1. Primary Hypotheses 1a: Adherence to guidelines for CVRS intervention vs. control group 39
8.2.2. Primary Hypothesis 1b: Control of SBP, LDL or HA1c will be significantly higher in subjects in the CVRS group compared to the control group. 40

8.3. Sample Size and Power Justification 40

8.4. Secondary Analysis 41
8.4.1. Secondary Hypothesis 1c: Adherence to guidelines following discontinuation of the intervention......................41
8.4.2. Secondary Hypothesis 3: To determine if the CVRS is cost-effective 42

8.5. Avoiding Missing Data 43

9. STUDY RESPONSIBILITIES 44

9.1. University of Iowa PI Responsibilities 44

9.2. Training 44

9.3. Communication with Sites 44

9.4. Documentation 44

9.5. Data Transmission and Record Retention 45

9.6. Use of the Iowa Personal Health and Research Management (PHRM) System 45

9.7. Study Closeout 46

9.8. Publication Policies 46

9.9. Invoicing the University of Iowa 46

10. ETHICAL CONSIDERATIONS 47

10.1. Role of University of Iowa 47

10.2. Informed Consent 47

10.3. Confidentiality of Subjects 47

10.4. Authorization for Use and Disclosure of Protected Health Information (HIPAA) 48

10.5. Human Subject Protections 48
10.5.1. Research Subject Selection 48

10.5.2. Compensation to Subjects 48
10.5.3. Risks/Discomforts of Study Participation 48

10.6. IRB Review 49

11. SITE TRAINING AND MONITORING 50

11.1. Initial Information and Training Sessions 50
11.1.1. Provider Information Session and Surveys 50

11.2. Study Coordinator Training 50

11.3. Teleconference Calls 50

11.4. Interim Monitoring Visits 51
11.4.1. Pre-Monitoring Procedures 51
11.4.2. On-site Monitoring 52
11.4.3. Post-Monitoring 52

11.5. Close-Out Visits 52

11.6. Organizing and Maintaining Study Files 52
11.6.1. Completed paper versions of case report forms (CRFs) 53
11.6.2. Regulatory Documents 53

11.7. Incorrect Subject Enrollment/Protocol Deviation 53

12. UNANTICIPATED PROBLEMS (UPS) 54

12.1. Key Definitions 54

12.2. Responsibilities of Involved Parties 54
12.2.1. Study site personnel 54
12.2.2. Local IRBs 55
12.2.3. University of Iowa IRB 55

Figure 2. Decision Tree for Reporting Unanticipated Problems 57

13. PROCEDURES FOR CVRS CLINICAL PHARMACISTS AND SITE PROVIDERS (INTERVENTION SITES ONLY) 58

13.1. Procedures for the CVRS Clinical Pharmacist 58
13.1.1. Medical Record Data Sources 58
13.2.1 Scheduled Subject Visits 58
13.2.1.1 Baseline Visit 58
13.2.1.2 Follow-Up Visits 59

14. SUBJECT TERMINATION 60

15. PROCEDURES FOR SITE PROVIDERS 61

15.1. Provider Surveys ... 61

15.2. Referring Subjects to the Study ... 61

15.3. Provider Interaction with the CVRS Pharmacist ... 61

APPENDIX I: PARTICIPATING SITES ... 62

APPENDIX II: QUICK GUIDE TO ICARE STUDY ELIGIBILITY CRITERIA ... 64

APPENDIX III: SCREENING LOG AND VERIFICATION OF INCLUSION AND EXCLUSION CRITERIA FORM ... 66

APPENDIX IV: BASELINE VISIT CHECKLIST ... 69

APPENDIX V: CASE REPORT FORMS ... 72

APPENDIX VI: PHARMACIST-PHYSICIAN COMMUNICATION FORM TEMPLATE ... 103

APPENDIX VII: PATIENT BROCHURE ... 105

APPENDIX VIII: STUDY DRUG CODES ... 108

REFERENCES ... 131

PROTOCOL APPROVAL PAGE

Study Title: Improved Cardiovascular Risk Reduction to Enhance Rural Primary Care (ICARE)

Version: 1.0

Date of Issue: [02/12/15]

Study Sponsor: National Heart, Lung and Blood Institute, National Institutes of Health

The signatures below constitute the approval of this protocol and the attachments and provides the necessary assurances that this trial will be conducted according to all stipulations of the protocol, including all statements regarding confidentiality, and according to local legal and regulatory requirements and applicable U.S. federal regulations.

We, the undersigned, have read and approve this protocol and agree on its content.

Barry L. Carter, PharmD		February 12, 2015
Principal Investigator (printed)	Principal Investigator (signature)	Date
Barcey T. Levy, MD, PhD		February 12, 2015
Co-Principal Investigator (printed)	Co-Principal Investigator (signature)	Date

PROTOCOL VERSION AND AMENDMENT TRACKING

Study Title: Improved Cardiovascular Risk Reduction to Enhance Rural Primary Care (ICARE)

Version Number/Amendment	Approval Date
Original Protocol, Version 1.0	[February 12, 2015]

PROTOCOL SYNOPSIS

Protocol Title	Improved Cardiovascular Risk Reduction to Enhance Rural Primary Care (ICARE)
Diagnoses and Main Criteria for Inclusion	Subjects who have at least one uncontrolled risk factor (diabetes, hypertension, or hypercholesterolemia) plus two additional cardiovascular (CV) risk factors.
Study Objective	To determine the extent to which a care delivery model utilizing a centralized Cardiovascular Risk Service (CVRS) will be implemented in private medical offices in relatively rural areas and which lack on-site clinical pharmacists.
Study Design	A 12 site, two-arm, cluster-randomized trial
Number of sites	12
Study arms	At the outset of the trial, randomization will assign each site in a 1:1 fashion to one of two arms: a) the CVRS group or b) the usual care group, with all subjects at a given site participating per the site's randomization.<br>Each site will consent 25 subjects to the site's study arm.
Total Number of Subjects	300
Duration of Study Participation	12 months active, with additional medical record abstraction at 30 months
Primary Outcome	The degree to which care adheres with the Guideline Advantage standards of care that apply for secondary prevention of CVD and other prevention strategies.
Secondary Outcomes	1. Adherence to Guideline Advantage standards of care 18 months after the intervention is discontinued, i.e., 30 months.<br>2. Blood pressure (BP) control, mean BP, low density lipoprotein (LDL) cholesterol, Hemoglobin A1c (HA1c)<br>3. Measurement of Stages of Change<br>4. Intensity of medication management

	5. Medical Home Index<br>6. Provider attitudes to deliver intervention, barriers and facilitators to implementation
Statistical Analysis Methods	Guideline Advantage Scoring: We will create a score for each subject at baseline, 12 and 30 months based on the number of applicable Guideline Advantage criteria that are met.<br>For each subject, the primary outcome will involve a determination of the percentage of applicable Guideline Advantage criteria met at the end of the twelve month active participation period. The analysis will use a mixed model, adjusted for adherence at baseline and the minority status grouping.<br>Percent of criteria adherence will also be calculated for the 30 month time point and analyzed using the same model.<br>To evaluate barriers and facilitators to implementing the intervention, the relationship between provider-level attitudes and beliefs and adherence scores will be calculated.<br>A cost-effectiveness analysis will assign a cost to CVRS pharmacist time (including record review, subject assessment, email time, telephone follow-up), clinic visits, emergency room visits, hospitalizations, and laboratory procedures. Incremental costs as a function of differences in guideline adherence, LDL cholesterol, blood pressure, or HA1c will be calculated at baseline, 12 months, and 30 months. These findings will be expressed as dollars per incremental improvement in guideline adherence or individual outcomes such as LDL cholesterol, blood pressure, or HA1c.
Interim Analyses	The Data and Safety Monitoring Board has determined that an interim analysis is not necessary.

SITE LEAD PROVIDER STATEMENT

I have read the protocol, and I agree that it contains all necessary details for me and my staff to conduct this study as described. I will personally oversee the conduct of this study as outlined herein and will make a reasonable effort to complete the study within the time designated.

I will provide all study personnel under my supervision with copies of the protocol and access to all information provided by the study sponsor (NHLBI) and the University of Iowa. I will discuss this material with them to ensure that they are fully informed about the efficacy and safety parameters and the conduct of the study in general. I am aware that, before beginning this study, the institutional review board responsible for such matters must approve this protocol in each clinical facility where it will be conducted. I agree to make all reasonable efforts to adhere to the attached protocol.

Site Lead Provider (printed name)	Site Lead Provider (signature)	Date

ABBREVIATIONS

ADA	American Diabetes Association
AHA	American Heart Association
AHRQ	Agency for Healthcare Research and Quality
BP	Blood pressure
CAD	Coronary artery disease
CCC	Clinical Coordinating Center
CCM	Chronic Care Model
CE	Cost-Effectiveness ratio
CRF	Case Report Form
CVD	Cardiovascular disease
CVRS	Cardiovascular Risk Service
DBP	Diastolic Blood Pressure
DMC	Data Management Center
DSMB	Data and Safety Monitoring Board
GCP	Guidelines for Good Clinic Practice
HA1c	Hemoglobin A1c
HIPAA	Health Insurance Portability and Accountability Act
ICARE	Improved Cardiovascular Risk Reduction to Enhance Rural Primary Care
PHRM	Iowa Personal Health and Research Management system
IRB	Institutional Review Board
IRENE	Iowa Research Network
LDL	Low density lipoprotein
MI	Myocardial infarction
MTM	Medication Therapy Management
NHLBI	National Heart, Lung and Blood Institute

NIH	National Institutes of Health
PAD	Peripheral Artery Disease
PBRN	Practice-based Research Network
PHR	Personal Health Record
PI	Principal Investigator
SBP	Systolic Blood Pressure
SC	Study Coordinator
SD	Standard Deviation
TIA	Transient Ischemic Attack
TPB	Theory of Planned Behavior
TTM	Transtheoretical Model
UP	Unanticipated Problem

1. Introduction

1.1. Background

An American Heart Association (AHA) report states that **"…more than 2,200 Americans die of CVD every day… 1 death every 39 seconds."**[1] The cost was $286 billion, or 15% of total healthcare expenditures.[2] Stroke is the third leading cause of death; someone died every 4 minutes[1], and this will cost over a trillion dollars from 2005-2020.[3] Only 21-47% of women with ischemic heart disease or diabetes received recommended therapy.[4] We have found major regional and age variations in guideline concordant therapy following myocardial infarction (MI).[5-11] Fewer than half of eligible Americans have been screened for some cancers.[11, 12] These gaps are often associated with busy primary care providers who often must address acute presenting complaints for subjects with multiple chronic conditions. Medication regimen complexity markedly reduces adherence.[13] The contribution of the present study will be: 1) the development of an effective strategy to improve the management of CVD and preventive health services, and 2) to achieve key performance improvement measures[14] using an efficient, centralized, web-based CVRS to support primary care providers. This study will meet important targets in the National Heart, Lung and Blood Institute (NHLBI) strategic plan, the Million Hearts Campaign, the American Cancer Society (ACS), the American Diabetes Association (ADA) and the AHA as outlined in the Guideline Advantage program.

The Patient Centered Medical Home is care in which the patient has an ongoing relationship with a personal physician who leads the medical team.[15-22] The model framework we use is the Chronic Care Model (CCM)[23-34] which includes pharmacists to manage complex conditions.[23, 24, 28, 30] Over 100 studies have demonstrated improved outcomes for risk factors for CVD following physician/pharmacist collaboration.[35-61] A systematic review of 30 trials found significant improvements in risk factor control with pharmacist management (Santschi et al 2011).[62] However, most of these studies involved single disease states or single clinics. It is not known if this intervention will be effective for multiple chronic conditions or if the model will advance adoption of key components of the Medical Home. Our innovations will be to include clinical pharmacists in our CVRS to promote preventive services and make recommendations on medication adherence and proper disease management. We will measure adoption of key components of the Medical Home by administering the Medical Home Index which is a validated, self-assessment tool to evaluate primary care practice.[63, 64]

The intervention will influence behaviors for patients and physicians. We will promote and evaluate inter-relationships to predict successful implementation.[65] We used the Theory of Planned Behavior (TPB) to evaluate **physician adoption** of our interventions in our ongoing trial.[58, 66-70] We have also pioneered strategies to measure the willingness of physicians to collaborate with pharmacists.[58, 60, 67] These approaches will be used in the training and evaluations in the present study. Our **patient intervention** is based on the **transtheoretical model (TTM)** to assess readiness for adherence, pros and cons associated with adherence and self-efficacy and behavioral activities that facilitate adherence (Prochaska et al).[71-74] We will use a structured motivational interviewing approach that facilitates behavior change by helping patients explore and resolve their ambivalence toward the change, thus affirming individuals' autonomy and self-determination while increasing their self-efficacy regarding

behavior change.[75] Patient stage of change for the most central health behaviors (e.g., medication adherence, dietary change, exercise, smoking cessation) will be evaluated via interview and determined utilizing a structured TTM-based assessment protocol for each enrolled patient.[76] Allocated stage of change will be included as an intermediate patient outcome.

1.2. Clinical Experience with the Study Intervention

Medicare Part D requires medication therapy management (MTM) for more complex patients; unfortunately, most MTM services focus on adherence to formulary guidelines. However, the major problems are that patients do not receive indicated medications and are not monitored for medication adherence.[10, 39, 85] Most MTM staff do not have access to medical record data. The Medicare Disease Management Pilot Program evaluated eight commercial programs that involved nurse-based call centers and found very little effect of the intervention.[86] The authors identified five possible reasons for these disappointing findings. The two most important were that the nurses could not address "chronic disease in real time" and that "the health coaches were not integrated into the beneficiary's primary health care team." <u>Our intervention model overcomes these and other critical deficiencies.</u>

The University of Iowa research team has extensive experience using team-based care to improve care management of patients with complex medical histories. Dr. Chris Parker, a pharmacist and member of the study team, has provided MTM by telephone for insurance companies for hundreds of patients throughout the U.S. <u>He has previous experience working in a pharmacy with access to electronic medical records for the majority of physician offices in Dubuque and Cascade, Iowa, demonstrating that integration is feasible.</u>

Our research team is a national leader in studying models of primary care delivery,[39, 41, 42, 48, 61, 85, 87-93] and we have pioneered strategies to evaluate team care implementation and guideline adherence using cluster-randomized trials.[70, 77] Drs. Carter (PI), Levy (Co-PI), Chrischilles and James have conducted 12 health services outcome studies funded by NHLBI, the American Cancer Society or AHRQ in the last 8 years that evaluated theoretical models and instruments for physician-pharmacist collaboration,[58, 59, 66] guideline adherence,[6, 91, 94-101] and cancer screening.[11, 12, 78-84] Dr. Carter has conducted nine health services outcome studies funded by National Heart, Lung and Blood Institute (NHLBI) and the Agency for Healthcare Research and Quality in the last six years. The current ICARE study will be conducted primarily within our practice-based research network (PBRN), led by Dr. Levy. The CVRS model will provide services by telephone, text messages or asynchronous web discussions. The CVRS intervention pharmacist will be integrated into the on-site primary care team and frequently have two-way communication with providers.

1.3. Rationale

Experts call for more research to evaluate pharmacists in the management of chronic medical conditions.[104-107] We are conducting a prospective cluster-randomized trial in 12 offices within the Iowa Research Network (IRENE), a PBRN. The objective of Aim 1 is to improve adherence to cancer screening, prevention and CVD guidelines by implementing a CVRS. To complete this objective we will test two <u>**primary hypotheses**</u>: "Adherence to the Guideline Advantage metrics will be significantly

greater in subjects from clinics randomized to the centralized CVRS group compared to the control group" and "Control of BP, LDL cholesterol, or HA1c will be significantly higher in subjects in the CVRS group compared to the control group." Our secondary hypothesis is that: once discontinued, guideline adherence will remain significantly higher in the intervention group than the control group. The **rationale for this work** is that completion of these aims will provide critical information about whether a model currently used in integrated health systems such as Kaiser will be implemented in rural, private medical offices. Adoption of the Medical Home is a process that includes six domains.[63] We expect our intervention will move offices to higher levels, or greater "Medical Homeness" in chronic care management, care coordination and quality improvement. However, we will assess all 6 domains to evaluate the overall adoption of the Medical Home. This information will be critical to the development of effective strategies to reduce morbidity and mortality in millions of patients with multiple chronic conditions who are not managed within integrated systems of care.

2. Study Organization

2.1. Personnel

The **Clinical Coordinating Center (CCC)** within the College of Pharmacy at the University of Iowa is responsible for the following key aspects of the trial: selection of participating sites; assisting sites in obtaining approval to conduct the study from their local Institutional Review Board (IRB); negotiating with sites the work that is to be completed and the compensation that sites will receive; oversight of data submission; training of site staff.

**Clinical Coordinating Center Team**		
Barry L. Carter, PI	barry-carter@uiowa.edu	319-335-8456
Brian Gryzlak, Project Coordinator	brian-gryzlak@uiowa.edu	319-353-3857<br>319-335-8218
Nick Rudzianski, Data Entry, Administrative Support	nicholas-rudzianski@uiowa.edu	319-335-9783
CCC Fax Number		319-335-9782
**Prevention Health & Cardiovascular Risk Service (CVRS) Pharmacists**		
Christopher Parker, Clinical Pharmacist	christopher-parker@uiowa.edu	866-227-9873
Rachel Finkelstein, Clinical Pharmacist	rachel-finkelstein@uiowa.edu	866-227-9873
Tyler Gums, Clinical Pharmacist	tyler-gums@uiowa.edu	866-227-9873

The **Data Management Center (DMC)** within the Department of Family Medicine at the University of Iowa is responsible for the following key aspects of the trial:monitoring procedures at research sites and data analyses.

**Data Management Center**		
Barcey T. Levy, Co-PI & Director, DMC	barcey-levy@uiowa.edu	319-384-7000
Carol Moss, Study Monitor	carol-moss@uiowa.edu	319-356-4486
Yinghui Xu, Data Manager	yinghui-xu@uiowa.edu	319-384-5497

The **Iowa Personal Health & Research Management System (PHRM)** is a web-based tool that simultaneously gives patients an opportunity to increase involvement in managing their health and serves as the study management system and data entry interface. The PHRM will be managed by the following team within the College of Public Health and the Institute for Clinical and Translational Science at the University of Iowa.

**Iowa Personal Health & Research Management (PHRM) System Team**		
Brian Gryzlak, Research Specialist	brian-gryzlak@uiowa.edu	319-335-8218<br>319-353-3857
Michael Mueller, Lead PHRM Database Administrator	michael-mueller@uiowa.edu	319-384-1547
Ryan Lorentzen, Application Analyst	ryan-lorentzen@uiowa.edu	319-353-8015

2.2. Regulatory and Billing Requirements

Each site will be required to complete the following study-related tasks and to store and transmit to the University of Iowa CCC the relevant documents listed for each task.

2.2.1. IRB Oversight

Each clinic must be overseen by an IRB of record. There are two options for the IRB of record:

1) Local IRB: The clinic may use a local IRB which is already in place to oversee research affiliated with the clinic. Information needed for a local IRB application will be provided by the CCC. The clinic will need to provide the following documents on an ongoing basis:
   - The IRB's letters of approval for the study
   - All stamped informed consent documents approved and dated by the local IRB
2) University of Iowa IRB: Clinics that do not have oversight by a local IRB may use the University of Iowa IRB as their IRB of record. The following documentation will need to be submitted:
   - A letter of agreement addressed to the University of Iowa PI
   - A Health Insurance Portability and Accountability Act (HIPAA) letter from the clinic's Privacy Officer
   - Documentation of Human Subjects Protection Training
   - The clinic's FWA number or an Individual Investigators Agreement to conduct the project in accordance with the University of Iowa's FWA
   - An IRB Authorization Agreement

The University of Iowa CCC will assist clinics in obtaining required documentation for either a local IRB or the UI IRB.

2.2.2. Subaward Agreement with the University of Iowa

Administrative personnel at each site must also negotiate and sign a Subaward document created by the University of Iowa's Department of Sponsored Programs. The agreement describes the terms and conditions for reimbursing sites for study-related costs. The agreement should be signed by an authorized individual at the clinic and returned to the University of Iowa. The subaward budget will be negotiated on an annual basis, with an authorized signature required each year on the amended award.

Please note that payments are based upon work that is completed. A clinic that does not complete expected tasks for a given year would not receive all the funds designated in the budget for that year. Questions regarding the subaward agreement should be directed to Brian Gryzlak at **brian-gryzlak@uiowa.edu**

3. Objective, Aims, and Hypotheses

3.1. Objective

The **objective of this study** is to conduct a multi-center, cluster-randomized trial utilizing a centralized CVRS to support private medical offices in more rural areas that lack large integrated systems. This study is titled **"Improved Cardiovascular Risk Reduction to Enhance Rural Primary Care: ICARE."** We will randomize 12 primary care offices in the Iowa Research Network (IRENE) to CVRS or usual care and enroll 300 subjects.

3.2. Aims and Hypotheses

Aim 1: To determine if a web-based CVRS will be implemented within private primary care offices.

Primary Hypothesis 1a: Adherence to the Guideline Advantage metrics will be significantly greater in subjects from clinics randomized to the centralized CVRS group compared to the control group.
Primary Hypothesis 1b: *Control of* blood pressure (BP), Low Density Lipoprotein (LDL) cholesterol *or* HA1c will be significantly *higher* in subjects in the CVRS group compared to the control group.
Secondary Hypothesis 1c: Adherence to the Guideline Advantage metrics will remain higher in the intervention group compared to the control group at 30 months (18 months after the intervention is discontinued).

Aim 2: To determine if the CVRS is cost-effective.

Secondary Hypothesis 3: The CVRS will have a favorable cost-effectiveness when compared to the control group.

We are confident that the intervention will achieve high rates of guideline adherence, risk factor control and cancer screening. Our approach is **innovative** because it will ask the questions: "Will the CVRS model be successfully implemented?" as well as "Is care management effective?" These questions have not been addressed for patients with multiple chronic conditions in private practice. This study design is novel because it will: 1) be the most robust study to test this model using a cluster randomized design in small private practice clinics, and 2) evaluate whether the effect can be sustained long-term.

4. Subject Selection / Eligibility Criteria

4.1. Inclusion Criteria

1) English-speaking males or females
2) ≥50 years of age
3) Patient seen at least once in clinic/practice in the previous 24 months
4) History of at least one of the following chronic medical conditions and associated uncontrolled risk factor:
    a. diabetes with HA1c > 7.5%
    b. hypertension, with:
        i. ≥150 mm Hg Systolic Blood Pressure (SBP) or ≥ 90 mm Hg Diastolic Blood Pressure (DBP) for patients with uncomplicated hypertension **<u>OR</u>**
        ii. ≥140 mm Hg SBP for patients with diabetes or chronic kidney disease with systolic BP > 150 mm Hg or diastolic BP > 90 mm Hg
    c. hypercholesterolemia, with
        i. >110mg/dl for patients with Peripheral Artery Disease (PAD), Coronary Artery Disease (CAD), Stroke, Transient Ischemic Attack (TIA), or Diabetes OR
        ii. >140mg/dl
5) A total of three or more of the sum of the conditions and risk factors above plus a history of any of the following:
    a. CAD
    b. MI
    c. stroke
    d. TIA
    e. atrial fibrillation
    f. peripheral vascular disease/claudication
    g. carotid artery disease
    h. current smoker
    i. obesity

4.2. Exclusion Criteria

1) cancer with a life expectancy less than 24 months
2) pregnancy
3) diagnosis of pulmonary hypertension (NOTE: *secondary pulmonary hypertension is OK)*
4) inability to give informed consent
5) nursing home residence or diagnosis of dementia
6) no telephone or have a hearing impairment that does not allow them to use the telephone
7) refusal to consider attempting to use the internet at home, community center, library, medical office or other source to access the study PHRM
8) Omron BP cuff cannot be used on subject's arm for any reason (e.g., need thigh cuff)

9) patient has plans to move from the area or transfer care to a different clinic in the next 12 months

Documentation of a medical condition in the patient's problem list is preferred, but explicit mention of a diagnosis in a provider note can be used if the problem list has not been regularly updated.

5. STUDY DESIGN

5.1. Study Overview

Dr. Dawson (biostatistician) will randomize 12 medical offices in a cluster-randomized design to avoid contamination within offices. All physicians are members of IRENE, have agreed to participate and have been invited only if they have staff (office nurse or medical assistant [MA]) that have completed (or will complete) human subjects training and have agreed to assume the role of Study Coordinator (SC), enrolling subjects and completing all study procedures.

5.2. Site Randomization and Treatment Arms

Dr. Dawson will randomize the 12 offices in a cluster-randomized design to one of two arms: 1) the CVRS intervention arm or 2) the usual care arm. All subjects enrolled at a given site will participate in the study arm to which the site was randomized.

All subjects will be able to track their medications, blood sugar readings, blood pressure measurements, and diagnosed conditions and will also receive links to related publications and news through the Iowa Patient Health and Research Management (PHRM) online portal.

Only subjects at intervention arm clinics will receive the CVRS intervention. A CVRS clinical pharmacist at the University of Iowa will work telephonically or through other agreed-upon modes of communication with each intervention arm subject and communicate with his/her providers to optimize subjects' pharmacological regimens and lifestyle patterns. Each subject at intervention arm clinics will receive the CVRS intervention for 12 months. Subjects at intervention arm clinics can also communicate with their Iowa clinical pharmacist using the PHRM. Subjects enrolled at usual care clinics will receive the clinic's usual medical care and will not have any exposure to the CVRS intervention or pharmacists.

The intervention will be evaluated on the degree to which the care provided to intervention arm subjects, when compared to control arm subjects, adheres to the Guideline Advantage measurement set criteria.

5.3. Schedule of Measurements

Table 1: Data Elements, Sources and Timing for Subjects				
**Data Element**	**Source of Information**	**Baseline**	**12 Mo.**	**30 Mo.***
**Primary Endpoints**				
▪ Aim 1a: Adherence to Guideline Advantage criteria (Prospective subjects, n=300)	Direct measurement and Medical records	X	X	X
▪ Aim 1b: BP control, mean BP, LDL cholesterol, HA1c, immunization, cancer screening	Direct measurement and Medical records	X	X	X
**Secondary Endpoints**				
▪ Aim 1c: Adherence to Guideline Advantage criteria (Retrospective data, n=300)	Direct measurement and Medical Records		X	X
▪ Intensity of medication management	Patient Interview/Medical records	X	X	X
**Control Variables and Process Measures**				
▪ Age, race, sex, weight, BMI, education level, economic status, marital status, insurance status	Patient Interview/medical records	X		
▪ Co-morbidity (chronic conditions)	Medical records	X	X	X
▪ Number and frequency of care contacts	Medical records	X	X	X
▪ Number of chronic medications	Medical records/interview	X	X	X
▪ Medication adherence	Patient interview	X	X	
▪ Smoking status	Medical records/Patient interview	X	X	X
▪ Evaluation of *Stages of Change*[76]	Patient interview	X	X	
*** The 30-month date will be *chart audit only.***				

5.4. Recruitment Procedures

When screening clinic patients for possible enrollment into the study, it is imperative to determine that they fully meet all of the inclusion criteria for the study and that they do not meet any of the exclusion criteria.

The SC completes a Screening Log/Verification form for each potentially eligible subject.

Three recruitment processes are available for sites to employ:

1) Approach in-person: Review the medical record to identify patients that have a visit scheduled at the participating office in the next week(s); SC approaches the patient in person to discuss the study.

2) Contact by phone: SC uses the approved phone script to call the patient and briefly describe the study. They may send the patient a copy of the informed consent document prior to scheduling a baseline visit if the patient requests.

3) Contact by mailed letter: SC sends an approved invitation letter to the patient.

To employ Options 2 and 3 above, the SC runs or requests from their Information Technology staff a list of all patients who are age 50 or older, were seen in the clinic during the past 24 months, and who have ICD-9 codes for or diagnoses of diabetes, high cholesterol, or hypertension. Clinic providers may also recommend patients to the SC.

Interested patients will be asked to set up a time for an initial study visit in the clinic with the SC.

5.4.1. Screening Log and Verification of Inclusion and Exclusion Criteria ("Screening Log/Verification Form")

For each subject screened, the SC completes a Screening Log/Verification form. Each subject who is screened for the study is tracked on this log to record their eligibility qualifications, willingness to participate and potential date of consent. All Screening Log/Verification forms are retained for the duration of the study, and a de-identified copy of these forms, i.e., one with the subject's name, MRN, and phone number marked out or otherwise removed, is sent to the CCC quarterly. In addition, a copy of the Screening Log/Verification form for all subjects who are enrolled in the study is faxed to the CCC after baseline visit procedures.

5.4.2. Screening questions

Prior to scheduling the study visit, the SC should ask the following questions, which correspond to four of the exclusion criteria, of all subjects:

1. *If you already have internet access at home, would you be willing to use the study website? Or, if you don't have access at home, would you be willing to try to access the internet at another location, whether at someone else's home, at the library or in the clinic, to use the study website?* ***(ANSWER MUST BE YES)***
2. *(Female subjects only): Are you currently pregnant?* ***(ANSWER MUST BE NO)***
3. *Do you have plans to move from the area or to transfer care to a different clinic in the next 12 months?* ***(ANSWER MUST BE NO)***
4. *Do you currently live in a nursing home?* ***(ANSWER MUST BE NO)***

Subjects answers should be recorded on the Screening Log/Verification form.

6. Study Visits and Data Collection

All subjects will have an initial visit with the SC and a second visit 12 months later. Each visit will include research BP measurement, phlebotomy for a lipid panel, HA1c, and completion of surveys by the SC.

6.1. Schedule for Completing Case Report Forms

All case report forms (CRFs) should be completed on paper. Paper forms are considered to be source documents and should be securely stored in the subject's research folder in a locked file cabinet in a locked office. SCs should fax the paper CRFs to the CCC within 48 hours of enrollment.

Scheduled visits beyond Baseline should be completed during the 60-day window surrounding the due date, that is, 30 days before the designated time point – 30 days after the designated time point.

Data Collected	Time Points /Study Visit						
	Pre-Enrollment	Baseline visit	4 months	8 months	12 month visit	30 months	Event Driven
Screening Log and Verification of Inclusion and Exclusion Criteria	X	X					
Enrollment		X					
Diagnosed Conditions and Care Management		X			X	X	
Medication Reconciliation		X			X		
Health Behavior Inventory		X					
Blood Pressure, Laboratory and Cancer Screening		X			X		
Clinic Visit Tracking					X	X	
Unanticipated Problem - 12 Month Screening					X		
Unanticipated Problem - 30 Month Screening						X	
30 Month Blood Pressure, Laboratory & Medications						X	
Site Report of Unanticipated Problem							X
Study Termination							X

*If a site has facilitated pharmacist access to their EMR, the SC does not need to collect these data.

6.1.1. Pre-Baseline Visit Procedures

Before meeting with the subject for the Baseline visit the SC will:

1) Confirm that the IRB-approved consent document is the current version being used and is still valid. NOTE: Consent documents are re-issued on a yearly basis; once a subject has been consented on one document, it is not necessary to re-consent them on the updated version. You should not have a subject sign a consent that has expired. Unsigned consents that have expired or are otherwise not current should be destroyed, with the exception of one copy that needs to be retained for the regulatory binder where all versions of the consent should be kept.
2) Complete the Screening Log/Verification form through Question 31.
3) Confirm that the date entered on Item 27 of the Screening Log/Verification form is less than 6 months from the date you expect the subject to sign the consent form.

6.2. Enrollment and Steps for Obtaining Informed Consent

Only the SC(s), trained and certified in use of the Omron BP monitor by ICARE study staff, may obtain informed consent from subjects and enroll subjects into the study. Other clinic staff may refer patients to the study but may not review the consent document with subjects. Staff members are welcome to take patient questions regarding the study and refer them to the SC as needed.

The SC must obtain signed informed consent from the subject before undertaking any baseline research procedures (e.g., taking BP). The steps for obtaining informed consent are as follows:

1) Review screening criteria in Section D of the Screening Log/Verification form. Confirm that each subject meets the inclusion criteria and has none of the exclusion criteria.

2) Provide the subject with a complete informed consent document, encourage the subject to read the entire document, and allow the subject sufficient time to read the document.

3) Explain the following aspects of the study:

    A. Purpose of the research study: To examine whether a pharmacist-managed cardiovascular risk service can work with clinic physicians to decrease risk of cardiovascular disease.

    B. Duration of study participation: 30 months

    C. Number of research visits:

        1. Two visits with the SC, including the initial visit and a visit roughly 12 months later.

    D. Experimental portion of the study: Having a clinical pharmacist at a central location work with subjects by phone and in collaboration with the subject’s primary care provider.

    E. The study procedures/requirements:

        1. Must be willing to get access to the internet; will not be required to use the internet

2. Women cannot be pregnant.

3. Two study visits with the SC

4. Multiple surveys

5. Measurement of height, weight and blood pressure

6. Blood draw

F. If subject is a patient in an intervention office, multiple contacts with a clinical pharmacist via telephone, text or email; the pharmacist will work with patient's physician to decrease their risk of developing cardiovascular disease.

G. Data is collected from subject's medical record for up to 30 months after subject signs consent.

H. The risks of the study

1. Drawing blood could cause discomfort or bruising.

2. It is possible that subject's physician might try harder to improve their health and might prescribe more medications because subject is in the study. Such decisions would be made by the subject and their physician and are not part of the study itself. The subject could have a medication reaction, though their doctor will approve all changes to their medications.

3. There is a very slight chance that information about the subject's health could accidentally reach someone who is not connected with the study, but members of the research team will store all paper documents about the study in a locked file cabinet, and the website for the PHRM is password protected and housed on a secure research server.

I. The voluntary nature of the study, i.e., the subject may stop the study at any time.

J. When a subject's participation in the study may be stopped, e.g., for reasons of safety, compliance or if the sponsor stops the study.

K. HIPAA section – the clinic staff must be allowed to have access to the subject's medical information and to create medical information in order for the subject to be in the study.

L. Contact information in case of a research-related Injury.

4) Ask the subject what questions s/he has and provide answers. If SC is unable to provide answer to any question, tell subject that they will obtain answer from CCC at that time or otherwise as soon as possible.

5) If desired, the subject may take the unsigned consent document, wait up to 10 days to make a final decision and re-schedule the baseline visit if s/he decides to participate.

6) If after reading the consent document and having their questions answered a subject agrees to participate in the study, the subject will sign and date the consent document.

7) SC immediately signs and dates the consent document.

8) SC makes two COMPLETE copies of the consent document:

A. One copy is placed in the chart or medical record, unless the clinic does not permit this. (If clinic policy explicitly prohibits placing a copy of the signed consent in the subject's

medical record AND the local IRB does not require placing a copy in the medical record, then clinic policy should be followed.)

B. The subject will be given a copy, and

C. The original is placed in the subject's individual study file.

Note: The signed consent forms will be reviewed by study monitors from the University of Iowa during interim monitoring visits to ensure compliance with the informed consent process.

6.3. Blood Pressure Measurement

At each study visit, the SC will measure each of the following parameters and enter on the BP-Lab-Cancer Screening form:

1) Two pulse and 5-6 BP readings using the automated Omron HEM 907-XL (see *Procedures for Monitoring Research Blood Pressure,* Section 6.8)
2) Height and weight

6.4. Laboratory Specimen Collection

At each study visit, the SC (or designated laboratory technician) will draw blood required for the following tests:

- Lipid profile (preferably fasting)
- HA1c
-

Typically, a total of two 5 ml. vials of blood will be required for these tests. A fasting lipid profile is optimal. If a subject has documented triglyceride levels $\leq$ 100, then non-fasting lipids are acceptable.

If, based on prior subject triglyceride levels it seems probably or likely that triglyceride would be > 400, the SC should order a direct measure LDL. However, it is not always possible to anticipate this (e.g., subject may not be fasting); thus, in the scenario where it is not possible to calculate LDL, the SC should contact and schedule the subject to come in for second blood draw as soon as possible to obtain a direct measure LDL.

6.5. Subject Orientation to the Iowa Personal Health & Research Management (PHRM) System (Baseline visit only)

The Iowa Personal Health and Management System (PHRM) is an online record of patient health data that may be accessed by the subject, the SC at the subject's clinic, the study pharmacist, ICARE research staff in the University of Iowa Department of Family Medicine and the College of Pharmacy, and database managers in the College of Public Health.

Subjects should be given a written instruction sheet that will describe:

(1) the potential benefits of using the PHRM

(2) how to log onto the PHRM using a unique User ID and password and how to populate the fields

(3) if the subject is from an intervention office, how they can interact with the study pharmacist through the PHRM

This instruction sheet is found in the subject's study folder.

All subjects will have access to the Iowa Personal Health and Research Management (PHRM) online personal health record. The PHRM will enable subjects to keep track of medications, record health-related data (e.g. BP, blood sugar), and enter allergies and health conditions. It supports printing reports, including wallet-sized cards to facilitate communication with health professionals, and provides information for medications subjects have entered.

For intervention subjects, CVRS pharmacists will have the ability to update subject PHRM accounts with health conditions and medications. The PHRM will facilitate implementation and measure many of the aspects of the Medical Home. This strategy will provide a more efficient approach for managing large populations of subjects with multiple chronic conditions.

6.6. Transfer of CRF Data to the CCC

Once a subject is fully screened, consented and enrolled and the Baseline visit is completed, the SC should fax the five baseline forms and the Screening Log/Verification form to the CCC **within two working days**. This is important for timely review by the pharmacists for the intervention offices. Reference below to the CVRS pharmacist is only related to offices randomized to the intervention group.

- **The most important document to fax as soon as possible is the Enrollment form.**
    - Once this document is entered in the PHRM (if site has been randomized to the intervention arm), the CVRS pharmacist will be notified that a new subject has been enrolled.
- Laboratory results should be faxed to the CCC as soon as they become available. The CVRS pharmacist will use these results to guide the intervention.
- Similarly, CRFs should be faxed within 2 days after the completion of the 12-month follow up visit. Forms will be provided for the 30 month abstractions.

6.7. Data Entry

CRFs that are faxed to the CCC will be logged within 48 hours and data will be entered in the PHRM as soon as possible after receipt. All CRFs will be scanned to a secure research drive. If changes are communicated to the CCC after initial submission to the CCC by the SC, the CCC will implement changes both in the PHRM as well as the hard copy version of the CRF. The latter will then be re-scanned and will serve as the valid version subjects.

6.8. Subject Reimbursement

Subjects will be compensated through the University of Iowa eVoucher system. A member of the University of Iowa research team will initiate up to two payments based on completion of study visits. It is imperative that visit forms be faxed to the CCC promptly so that subjects receive timely reimbursement.

Total possible compensation = $150.00, to be distributed as follows:

$75.00 will be paid for each subject who completes the first (baseline) visit.

Subjects who fail any portion of screening at the first visit will not be compensated.

$75.00 will be paid for each subject who completes the 2nd visit (roughly 12 months later).

Any subject who does not complete the 2nd visit will not receive the 2nd payment.

The total payment amount is designed to compensate subjects for their year-long active participation in the study, including two phlebotomy draws.

Payment should arrive in the form of a check from the University of Iowa within four weeks of the completed visit. Should a subject not receive a check, the SC should contact Brian Gryzlak at 319-353-3857 or brian-gryzlak@uiowa.edu.

6.9. Procedures for Measuring Research Blood Pressures

Note: The manufacturer of the Omron HEM 907-XL gives a method for checking accuracy (compared to a calibrated mercury manometer) on page 26 of the Omron Instruction Manual and recommends doing this check if you get suspicious readings or if you drop the device. The manufacturer recommends re-calibration per the manufacturer every 5 years for light use (< 5 times daily), more often for heavy use.

6.9.1. Preparing the Subject

The subject ideally should refrain from smoking or ingesting caffeine for 20-30 minutes prior to the BP measurement.

Have the subject remove all clothing that covers the location of cuff placement.

The subject should be comfortably seated in a chair, with:

- the back supported
- legs uncrossed and flat on the floor
- the arm supported, ideally at heart level on a desk
- the palm of the hand facing upward

Have the subject sit for **at least 5 minutes**. Instruct the subject to relax as much as possible.

6.9.2. Cuff Measurement

The subject's arm (right is preferred) circumference should be measured at the baseline visit. This does not need to be repeated at follow-up visits unless the subject exhibits a marked change in weight.

The ideal cuff should have a bladder length that is 80% of arm circumference and a width that is at least 40% of arm circumference. The **INDEX ↑** that is marked on the edge of the cuff should fall within the range bar on the cuff. Recommended cuff sizes are:

- Arm circumference 17-22 cm: use "small adult" cuff
- Arm circumference 22-32 cm: use "adult" cuff
- Arm circumference 32-42 cm: use "large adult" cuff
- Arm circumference 42-50 cm: use "extra large adult" cuff

Subjects who require use of a thigh cuff cannot continue in the study.

Note midpoint circumference, which arm is cuffed and size of cuff used on the **BP-Lab-Cancer Screening** form.

6.9.3. Cuff Placement

Do not allow a sleeve to form a tourniquet on the arm.

Palpate the brachial artery in the antecubital fossa and place the **ART ↓** that is marked on the midline of the bladder of the cuff so that it is over the arterial pulsation of the subject's bare upper arm.

The lower end of the cuff should be ½ to 1 inch above the inner side of the elbow joint.

The middle of the cuff should be at the level of the right atrium (the mid-point of the sternum).

Pull the cuff snugly around the bare upper arm so that you can insert only one finger between the cuff and the arm.

6.9.4. Blood Pressure Measurement

Have the BP-Lab-Cancer Screening form and the Omron monitor beside you on the desk. Record time that first BP is taken.

Tell the subject that you will be taking a series of at least 3 sitting BP readings and that neither the subject nor the SC should talk during the measurements.

Push the ON/OFF button on the monitor to turn on the power.

<u>Take a single BP reading</u>:

- Set the MODE selector to "SINGLE."
- Set the P-Set knob to "AUTO."

- Push the START button.
- Record the displayed BP on line 2 of the **BP-Lab-Cancer Screening** form.
- Record seated pulse on line 1 of the **BP-Lab-Cancer Screening** form.

Wait 60 seconds before taking the next BP.

Take a double BP reading:

- Set the MODE selector to AVG.
- Push the START button.
- The machine will show you TWO individual BP readings – be ready to record the second one as the machine goes on quickly to display the average, automatically counting down from 60 seconds between readings. (If you miss either of the two readings, they can be recovered by pressing the "deflation" button which displays the first, second and average readings each time it is pressed.)
- Record both readings on lines 3 and 4 of the BP-Lab-Cancer Screening form.

If *either* systolic or diastolic readings of the second and third (double) BPs differ by > 4 mm:

- Set the MODE selector to "SINGLE." Wait 60 seconds.
- Push the START button.
- Record the displayed fourth reading on line 5 of the BP-Lab-Cancer Screening form.

Have the subject stand for one minute and then:

- Take another single BP reading according to the instructions, above, and record on line 7 of the BP-Lab-Cancer Screening form. Record pulse on line 6 of the BP-Lab-Cancer Screening form.

IF YOU GET AN ERROR MESSAGE AT ANY POINT, START THE SEQUENCE OVER. If subject appears ill or in distress or has SBP > 200 or DBP > 115, see "Handling Hypertensive Urgency", below.

If you continue to get an error message and the subject does not appear in distress, attempt 1-2 measurements on the subject's other arm. If the error message persists, take the BP manually.

Figure 1: Handling Hypertensive Urgency

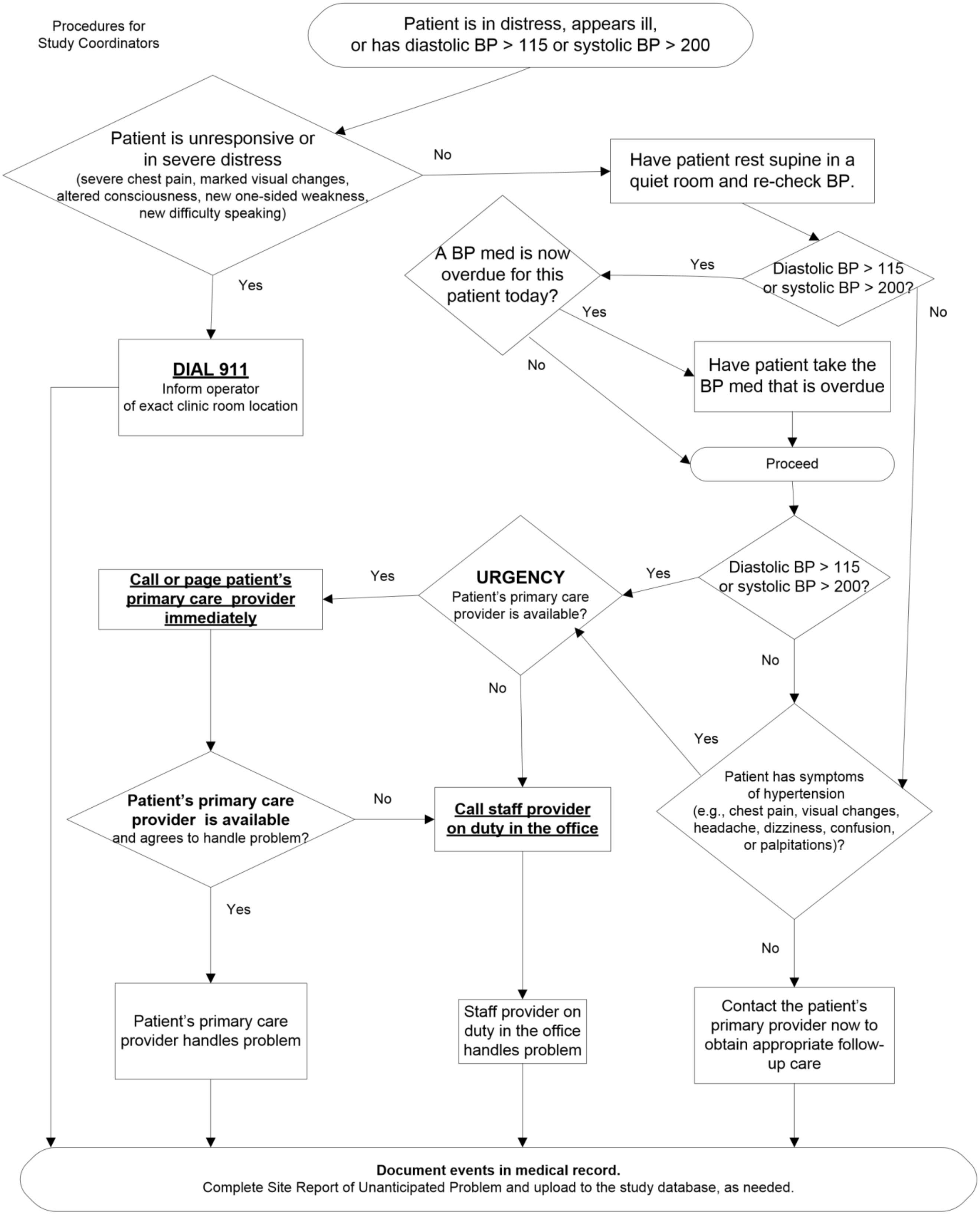

6.10. 12-Month Visit

The 12-month visit should be scheduled so that it occurs no sooner than 11 months and no later than 13 months after the baseline visit.

At this visit, the SC will:

1) Measure (BP-Lab-Cancer Screening form, check “12 month” box):
    a. Height and weight
    b. Pulse
    c. 3-4 BP readings using the automated Omron HEM 907-XL
2) Collect from subjects:
    a. Diagnosed conditions (Diagnosed Conditions-Care Management form, check “12 month” box)
    b. List of chronic medications and responses to the medication adherence survey questions (Medication Reconciliation form, check “12 month” box)
    c. Ask whether the subject has been hospitalized or visited the emergency room and screen the medical record for unanticipated problems (UP Event-Driven form)
3) Draw blood required for a lipid profile and HA1c, requiring a total of two vials of blood (results recorded on BP-Lab-Cancer Screening form)
4) Verify or obtain the following data from the subject’s medical record for the time period that spans the 12 month period following the initial baseline visit. This review of the medical record should be completed after the subject visit.
    a. Chronic medications (Medication Reconciliation form)
    b. Co-morbid conditions (Diagnosed Conditions-Care Management form)
    c. Most recent chart recorded BP (Diagnosed Conditions-Care Management form)
    d. Dates and results of labs and screening related to lipid management and diabetes management (LDL and HA1c) (BP-Lab-Cancer Screening and Diagnosed Conditions-Care Management forms)
    e. Evidence from the MR, i.e., from notes or Problem List, of unanticipated problems (UNANTICIPATED PROBLEM (UP) EVENT-DRIVEN FORM)
    f. Evidence from the MR of any UP documented on the 12-month follow up case report forms.

6.11. Medical Record Data Abstraction at 30 months

Each site SC will also collect medical record data that covers the time period extending from the 12 month study visit until 30 months following enrollment.

Abstracted data should not extend past the 30-month time point, and it should be submitted by 31 months following enrollment.

The following data will be collected for all subjects:

1) Diagnosed conditions (Diagnosed Conditions-Care Management form)
2) Clinic BP (30 Month Blood Pressure, Laboratory and Medication form)
3) Clinic visits (Clinic Tracking Form)
4) Laboratory tests (30 Month Blood Pressure, Laboratory and Medication form):
    a. HA1c
    b. Total Cholesterol
    c. HDL Cholesterol
    d. LDL Cholesterol
    e. Triglycerides
5) Prescribed medications related to cardiac and circulatory functioning (30 Month Blood Pressure, Laboratory and Medication Form)
6) Any evidence of an unanticipated problem (Unanticipated Problem (UP) Event-Driven Form)

7. STUDY OUTCOMES

7.1. Primary Outcomes

7.1.1. Adherence to Guideline Advantage Criteria in All Subjects

The primary outcome will be adherence to the Guideline Advantage criteria that apply for secondary prevention of CVD. (See link below to the Guideline Advantage Fact Sheet.) The criteria reflect drug therapies, meeting guideline goals for specific disease conditions, and screening and prevention measures.

http://www.guidelineadvantage.org/idc/groups/tga-public/@wcm/@tga/documents/downloadable/ucm_458200.pdf

Each eligible criterion will be scored based on whether or not it was met at each of the index dates (baseline, 12 months, and 30 months). An algorithm will take the number of Guideline Advantage criteria that apply to each subject and then calculate the percent of those applicable criteria that are met at each time point. The resulting single numeric value will be used as a surrogate for quality of care. This algorithm will be applied to all subjects, with CVRS intervention subjects compared to usual care subjects.

7.2. Secondary Outcomes

7.2.1. Adherence to Guideline Advantage Criteria at 30 months

The same algorithm will also be used to compare adherence between CVRS intervention subjects and usual care subjects at 30 months based on the chart audited values at this time point.

7.2.2. BP Control, Mean BP, LDL Cholesterol, HA1c

Direct measurement (at baseline and 12 months) and medical record abstraction (at 30 months) will yield values for BP control, Mean BP, LDL cholesterol and HA1c. Values will be compared for CVRS intervention subjects and usual care subjects.

7.2.3. Measurement of Stages of Change

Scores on the Stages of Change instrument will be compared for CVRS intervention subjects and usual care subjects at baseline and 12 months.

7.2.4. Intensity of Medication Management

The number of recommended medication changes per subject and the percent of recommended medication changes that were accepted/implemented will be compared.

7.2.5. Medical Home Index

The lead medical provider at each site will complete the Medical Home Index, a validated, self-assessment tool for evaluating primary care practice. Providers will complete this tool at the beginning of the project and again at the beginning of year 4 of the UI grant funding period to determine how each office has improved on adoption of the Medical Home and if there is greater adoption in intervention offices compared to usual care offices.

7.2.6. Provider attitudes to deliver intervention, barriers and facilitators to implementation

All clinic providers will be asked to complete two questionnaires at the beginning of the project and again at the beginning of year 4 of the UI grant funding period. The first validated instrument measures physician-pharmacist collaboration and will be used to evaluate the level and type of communication and any increases in the level of communication in the intervention group. The second validated questionnaire based on the Theory of Planned Behavior (TPB) will be used to evaluate physician adoption of the study intervention, with scores compared between CVRS intervention clinic providers and usual care clinic providers.

8. Statistical Analysis Plan

8.1. Statistical Design

This study will utilize a two-arm, randomized, cluster design. Twelve offices will be randomized in a 1:1 fashion into either the CVRS or usual care groups and 300 subjects will be enrolled (Aim 1). Each subject will be followed for 12 months, with an additional chart abstraction performed at 30 months to assess the extent to which increased guideline adherence is sustained after the intervention is discontinued.

8.2. Primary Analysis

8.2.1. Primary Hypotheses 1a: Adherence to guidelines for CVRS intervention vs. control group

The primary outcome will be adherence to the Guideline Advantage criteria and involve a determination of the percentage of applicable criteria met at the end of the twelve month period. This primary hypothesis will be assessed using a mixed model, adjusted for guideline adherence at baseline. This model will also use an exchangeable correlation structure to adjust for the correlation among subjects treated in the same clinic. For example, the following model will be fit to these data:

$$Y_{ij} = \beta_0 + \beta_1 X_{1i} + \beta_2 X_{2i} + \gamma_i + \varepsilon_{ij}$$

where Y_{ij} represents the adherence at twelve months for the jth subject in the ith cluster, X_{1i} is an indicator variable for whether the ith cluster was randomized to the CVRS or usual care group, X_{2ij} is the baseline guideline adherence score for the jth subject in the ith cluster, γ_i is the cluster i 'random effect' with variance σ_C^2, and ε_{ij} is the usual random measurement error term with variance σ^2. Hence, this model corresponds to the 'compound symmetry' assumption that implies that all members of a cluster are equally correlated with each other – and that members in different clusters are independent of each other. Correspondingly, the hypothesis of interest can be assessed by performing the following hypothesis test:

$$H_0: \beta_2 = 0 \quad vs. \quad H_A: \beta_2 \neq 0 .$$

This test will be implemented using an appropriate contrast statement with the model specified above. Furthermore, our analyses will use the intention-to-treat principle, e.g., all subjects will be analyzed according to their randomized group, even if there is inadvertent crossover.

Independent (Predictor) Variables. We will control for baseline age, gender, race, ethnicity, co-morbidity, number of medications, medication adherence, smoking status, education level, insurance status, economic status and marital status. We also control for encounter frequency since this alone may influence outcomes.[77, 108] Because randomization is performed at the site level, it is possible that some of these covariates may be imbalanced in this study. Thus, we will carefully monitor for any important imbalances among covariates. Should imbalances occur, we will control for these covariates in the linear regression model above. We will also assess the normality assumption involved in the model. If this assumption is violated, an appropriate transformation will be employed, or a nonparametric model will be fit.

8.2.2. Primary Hypothesis 1b: Control of SBP, LDL or HA1c will be significantly higher in subjects in the CVRS group compared to the control group.

Control of SBP, LDL cholesterol or HA1c will be the main outcomes of interest. The primary outcome is the dichotomous variable of at least one controlled disease. The primary analysis will use General Estimating Equations with the logit link function. This analysis accounts for the correlation among subjects from the same primary care office.

8.3. Sample Size and Power Justification

We used several studies that involved interventions for multiple risk factors to predict baseline and follow-up outcomes.[61, 77, 112-117] Subjects must have at least one of three uncontrolled risks (BP, lipids, HA1c). We conservatively estimate that 35% and 60% will achieve control of at least one risk factor in the control group and intervention group, respectively. Mean values for BP, LDL cholesterol and HA1c will be secondary outcomes and we estimate outcomes at baseline and 12 months to be (mean and SD) for BP, LDL and HA1c and the number of subjects and (percent) for controlled risk factors in the Table 1:

Table 1. Sample size and expected outcomes

Variable (Total Expected Sample)	Baseline	Control Group	Intervention Group
Systolic BP (mm Hg) (n=200)	148 ± 19	142 ± 19	134 ± 19
LDL Cholesterol (mg/dL) (n=180)	120 ± 40	110 ± 30	90 ± 30
HA1c (%) (n=150)	7.6 ± 1.7	7.6 ± 1.7	7.2 ± 1.5
**At least one controlled risk factor (n=300)**	**300**	**53 (35%)**	**90 (60%)**

The previous study found an intra-class correlation coefficient was 0.004 (Jeff Dawson, personal correspondence).[77] We expect 5% dropouts that will require imputation but we inflated this to 15% to be very conservative. Thus, the following assumptions were made: 1) approximate absolute 10% difference in guideline adherence at twelve months for subjects enrolled at CVRS sites versus subjects at usual care sites, 2) SD is expected to be 20% for both groups, 3) intraclass correlation coefficient is conservatively assumed to be less than or equal to 0.005, 4) both primary hypotheses will be tested at the 0.05 significance level, 5) drop-out rate was inflated to 15%. The approach used for determining the sample size is to first compute the number of subjects (not clinics) required in each group in a usual clinical trial setting (denoted by m—assuming independence of observations; this may also be termed the effective sample size). This sample size then needs to be inflated in order to account for: 1) the correlation between subjects at the same clinic, and 2) dropouts. The final sample size calculation is:

$$n^* = m\,[1/(1-d)]\,[1 + (n-1)\,\kappa],$$

where n is the number of subjects in each cluster (n= 25), κ is the estimated intra-class correlation coefficient (assumed to be 0.005), d is the assumed dropout rate, and n is the adjusted sample size. Based on power calculations for Hypothesis 1a, we have chosen a total sample size of n*=300, with n* ranging from 150 to 300 for Hypotheses 1b-1c. Although our analyses will involve extensive mixed model analysis based on n* subjects, as described above, the statistical power can be approximated by considering the power for two-sample t-tests based on m subjects (the effective sample size for

independent observations). Table 2 provides power levels for our primary hypotheses, based on the assumptions above. We have very good power for our primary outcome (Hypotheses 1a & 1c). The primary outcome for Hypothesis 1b is at least one controlled risk factor and power for this combined endpoint will be 0.967. Mean BP, lipids or HA1c are secondary outcomes. We expect very good power for LDL and reasonable power for SBP. While conservative, our power for HA1c may be low. However, based on numbers of subjects identified in these offices, we may have far more subjects with hypertension, hyperlipidemia and diabetes than expected so power could be much higher.

Table 2. Approximate Power and Effective Sample Sizes for Primary Hypotheses

Primary Outcome (Hypothesis)	n* (total sample size)	m (effective sample size)	Power
**Guidelines: 1a & 1c**	**300**	**226**	**.964**
**1 controlled risk factor: 1b**	**300**	**226**	**.967**
Secondary Outcomes (Hypothesis)			
LDL: 1b	180	136	.971
SBP: 1b	200	150	.726
HA1c: 1b	150	112	.258

Data will be collected at baseline and 12 months and medical records will be abstracted for 30 months to evaluate sustainability once the intervention is discontinued using our validated approach.[70, 77, 110, 111] In one study, 40% of applicable criteria were adhered to and Dr. Levy's study demonstrated only 18% of eligible patients in usual care received CRC screening.[77] In a retrospective evaluation of a pharmacist-led cardiac risk model, an average of 34% of the LDL, BP and HA1c guideline criteria were met.[112] In our evaluation of Medicare patients who suffered an acute MI, only 34% received all guideline concordant medications. Therefore, we expect baseline guideline scores (percent of applicable criteria met) to be 30-35% ± 20% (mean ± SD) but we have assumed they will be 40% ± 20% for sample size calculations so that our sample size estimate will be conservative. We expect scores to increase to 50% ± 20 in the control group and 60% ± 20 in the intervention group at 12 months. Further, we expect that guideline adherence scores will deteriorate in both groups after the intervention is discontinued but scores in the intervention group (50% ± 20) will remain significantly higher than the control group (40% ± 20) at 30 months (18 months after the intervention is discontinued).

8.4. Secondary Analysis

8.4.1. Secondary Hypothesis 1c: Adherence to guidelines following discontinuation of the intervention

This hypothesis will be assessed in the same manner as the first primary hypotheses, except the guideline adherence at 30 months will be used in place of the 12 month adherence outcome variable.

8.4.2. Secondary Hypothesis 3: To determine if the CVRS is cost-effective

Few studies of team-based care provided rigorous cost-effectiveness analyses. The cost to reduce CVD risk factors was $371 per year if a registered nurse delivered the intervention but no cost-effectiveness was provided.[115] We estimated the cost difference of a pharmacist intervention for hypertension compared to a control group to be $290 ($p < 0.001$; sensitivity analysis ranged from $223 to $512).[118] We will examine all the costs associated with the CVRS intervention, communication and overhead using our previous methods.[118] Dr. Linnea Polgreen has expertise as a labor economist. She will analyze the resource input costs to conduct the cost-effectiveness analyses. All CVRS pharmacist time (record review, patient assessment, email time, telephone follow-up), physician communication, clinic visits, emergency room visits, hospitalizations and laboratory procedures will have costs assigned.[118] Incremental costs as a function of differences in guideline adherence, BP, LDL cholesterol, or HA1C will be calculated at baseline, 12 months, and 30 months expressed as dollars per incremental improvement in guideline adherence or individual risk factors. A cost-effectiveness (CE) ratio is a standard method that directly clarifies the choices for decision makers and will be computed from the payer's perspective:

$$\mathrm{CE} = \frac{\text{Average Intervention Implementation Cost (AIIC)} + \text{Average Treatment Change Costs (ATTC)}}{\text{Outcome Improvement Resulting from the Intervention (OI)}}$$

The AIIC is the average cost/patient to implement the intervention and training costs calculated by summing the implementation costs and dividing by the total number of subjects in the intervention. The **ATCC** equals the average change in patient treatment costs that result from the intervention:

$$\mathrm{ATCC} = \mathrm{PC} + \mathrm{RC} + \mathrm{MC} + \mathrm{LC} \quad \text{, where}$$

PC = per patient change in physician, clinic and hospital costs for subjects affected by the intervention; RC = per patient change in pharmacist cost for subjects affected by the intervention; MC = per patient change in medication cost for those subjects whose treatments were affected by the intervention; and LC = per patient change in laboratory cost for those subjects affected by the intervention. To compute PC, RC, MC, and LC we carefully measure all patient contacts with healthcare providers, inter-provider contacts specific to patient care, and healthcare utilization (medication, lab tests). Patient-specific costs will vary with the number of units of each activity. These units will be multiplied by average provider times per activity, average provider wage rates, and average retail costs for medications and labs to estimate costs for each patient. PC, RC, MC, and LC will be estimated for the intervention. The calculation of this CE ratio involves the use of several parameter assumptions (e.g. activity times, wage rates, unit costs). We will assess the sensitivity of our estimates to plausible ranges of these assumed parameters from the payer's perspective. This value will aid payers to assess whether the intervention should be adopted in their organization and will require the payer to value guideline adherence more than the costs to reach adherence through the intervention. These valuations will likely be organization specific. However, as a rule, we will compare the CE ratio estimates from this intervention to the assumed guideline adherence valuations employed in the literature.

8.5. Avoiding Missing Data

It will be critically important to minimize missing data to prevent bias.[109] Unless subjects withdraw consent, we will collect their medical record data including laboratory values that are missed at the 12 and 30 month periods. After 4 years, the CAPTION trial had 7.5% missing data. Most of these data were from one terminated clinic. The present study will minimize missing data by requiring only two visits for data collection (baseline and 12 months) and using only intention-to-treat analyses. The only acceptable reasons for missing data will be subject withdrawal of consent or subject lost to follow-up. We will collect missing BP, LDL and HA1c from medical records which will be better than missing data. All other planned data for this study will be collected by chart audit, again to minimize missing data. Nevertheless, there is likely to be a small percentage of missing data that could bias the results. We will use a multiple imputation method with five separate imputations, and will average the parameters across all five imputations for the final analysis. While these methods rely on untestable methods, we purposely will avoid the "naively inadequate" methods listed by Fleming such as "last value carried forward" or completed-case analyses.[109] We are confident our imputations will minimize potential bias.

9. STUDY RESPONSIBILITIES

9.1. University of Iowa PI Responsibilities

By signing this protocol, the study's two PIs agree to be responsible for implementing and maintaining quality control and quality assurance systems to ensure that all work incidental to this protocol is conducted and data are generated, documented, and reported in compliance with the protocol, with accepted standards of Guidelines for Good Clinical Practice (GCP), and with all applicable federal, state, and local laws, rules, and regulations relating to the conduct of the clinical study.

The CCC PI will provide current copies of the study protocol to all investigators and site personnel responsible for study conduct.

The CCC PI will provide NIH with copies of all institutional review board (IRB) actions regarding the study.

9.2. Training

The CCC PI will hold a live or remote webinar/Skype session with site providers and research team members at the beginning of the study to review the study and the roles of key personnel. The CVRS pharmacists will participate in these sessions for sites randomized to the CVRS intervention group.

All SCs will attend an onsite training session, to be conducted by study team members in the SC's office to familiarize them with study procedures and to certify them in use of the Omron BP monitor.

9.3. Communication with Sites

The CCC PI and CVRS pharmacists will hold teleconference calls with the lead provider at intervention sites as needed during the first year of the study to develop strategies to optimize communication, improve implementation of the intervention, if necessary, and ensure fidelity to the intervention.

The CCC PI will hold teleconference calls on an as needed basis with the lead provider and SC at any site, intervention or usual care ("control"), that does not meet expectations for recruitment or data collection.

9.4. Documentation

Study documentation includes all electronic and paper forms ("CRFs"), data correction forms, source documents, monitoring logs and appointment schedules, sponsor-investigator correspondence, and regulatory documents (signed protocol and amendments, IRB correspondence and approval, clinical supplies receipts and distribution records).

By signing the protocol, the Lead Provider acknowledges that, within legal and regulatory restrictions and institutional and ethical considerations, study documentation will be promptly and fully disclosed to UI appropriate parties upon request. It will also be made available upon request for inspection, copying, review, and audit at reasonable times by representatives of UI or responsible government agencies as required by law.

9.5. Data Transmission and Record Retention

The SC will enter required data onto hard copy CRFs and fax these to the CCC as soon as possible after a data collection activity (study visit or medical record abstraction time point). If there are questions or missing data, a research team member will contact the SC to clarify. The data will then be uploaded by the UI research team into the study database through the PHRM. Timestamps will identify the occurrence of data entries and changes. Edit checks, electronic queries, and audit trails are built into the data collection system to ensure accurate and complete data collection.

SCs are responsible for maintaining paper copies of originally collected data on CRFs. If errors are identified on any CRF after the form was submitted to the CCC, the SC can either:

(1) make the correction(s) on the form and re-fax it to the CCC; the SC should indicate any corrections or errors by drawing a single line through the errant data (if applicable) and initial and date as per GCP standards, or
(2) Communicate the revision to the CCC via phone or email; in this scenario, the CCC will manually make the changes onto the CRF.

The CCC will maintain the current version of all CRFs. Thus in either case described above, the CCC will make the respective change to the study database via the PHRM and will re-scan the current paper version of the CRF containing the edits.

Sites will keep hard copy CRFs until the following criteria for destroying data are met: 1) the CCC informs the site that data may be destroyed and 2) the timeframe meets the policy of the local IRB (if applicable).

9.6. Use of the Iowa Personal Health and Research Management (PHRM) System

Subjects will be able to enter their own health information into the separate PHRM website application.

The PHRM has automated features that subjects may use to reset usernames and passwords, assuming that the subject has an active email address. Persons who do not have email should call the CCC and request a reset. The CCC will validate subject identity by requiring that subject responses match information in the database for (1) first and last name, (2) year of birth, (3) full address (current or previous if recently moved) and (4) at least one phone number documented for the subject.

9.7. Study Closeout

Once all study data have been entered and all queries have been resolved, the DMC will conduct closeout activities with the site, at the site and/or remotely.

9.8. Publication Policies

The PI will be primarily responsible for creation, review, and submission of publications and presentations relating to the major aspects of the study and approved ancillary analyses within a timely fashion after completion of the study.

The manuscript containing the overall study results will be distributed to all study investigators at the University of Iowa for review and comment before submission to a peer-reviewed journal with a reasonable period for review, but the final content will be at the discretion of the PIs. Any other manuscripts containing these data, including abstracts, will be distributed to all relevant study investigators who are participating on such publications before submission, with a reasonable period for review. Submitted publications will conform to international standards for biomedical manuscripts, including those regarding authorship.

9.9. Invoicing the University of Iowa

The Grant Accounting Department at the University of Iowa requests quarterly invoicing for project expenses. The following procedures will be maintained for submitting invoices:

1) Brian Gryzlak at the CCC will email a quarterly draft invoice to the clinic staff member who is designated as responsible for invoicing. Mr. Gryzlak should be notified immediately of any change in the person responsible for invoicing.
2) Each draft quarterly invoice will detail all costs that are reimbursable during that quarter; only tasks completed during the quarter will be included on the invoice.
3) Should a clinic's invoice not agree with any items on the draft quarterly invoice or contain items not included in the draft invoice, please notify Mr. Gryzlak at brian-gryzlak@uiowa.edu.
4) A completed invoice should be pasted onto clinic billing stationery and signed by an authorized individual in the clinic.
5) The signed invoice should be mailed to the address noted in the subaward contract. Contact Brian Gryzlak for guidance on this.
6) Payment typically occurs within 30 days of the completed invoice receipt.

10. ETHICAL CONSIDERATIONS

By signing this protocol, the PI agrees to conduct the study in compliance with the protocol, the Declaration of Helsinki, and all applicable federal, state, and local laws, rules, and regulations relating to the conduct of the clinical study.

10.1. Role of University of Iowa

The University of Iowa has overall responsibility for the conduct of the study, including assurance that the study meets the sponsor's regulatory requirements.

10.2. Informed Consent

The site Lead Provider has both ethical and legal responsibility to ensure that each subject being considered for inclusion in this study is given a full explanation of the study. Informed consent will be obtained from all subjects before any data are collected and before any study-related procedures are performed. University of Iowa investigators will help facilitate site submission of IRB applications.

Before and after subject provision of informed consent, research team members will be available via email, PHRM, or phone to answer questions or concerns regarding procedures and risks. Research team contact information will be included on all study materials and the study website.

10.3. Confidentiality of Subjects

Subject confidentiality will be maintained throughout the clinical study. A unique subject ID code will be used to identify all data reported for each subject. Names and other direct identifiers will only be visible to members of the research team who need access to such information.

Subject information collected in this study will comply with the standards for protection of privacy of individually identifiable health information as promulgated by HIPAA and as mandated in Title 45 CFR Parts 160 and 164. All records will be kept confidential, and the subject's name will not be released to non-authorized persons or entities at any time. Subject records will not be released to anyone other than members of the research team at each site who have a need for such information, and responsible regulatory authorities when requested. In all cases, caution will be exercised to assure the data are treated confidentially and that the subject's privacy is guaranteed.

Hard copy records containing subject data collected at sites (e.g., CRFs, informed consent documents) will be stored in a locked cabinet in a locked office at each respective site. Identification numbers will be used in place of names on CRFs. All electronic study data will be stored on encrypted, password-protected servers located within security firewalls, such that only members of the research team who need access will be allowed access to study files. Subject data will not be reused or disclosed to any other person or entity, except as required by law, for authorized oversight of the study, or for other research for which disclosure of the requested data would be permitted by the HIPAA Privacy Rule. If needed, any transport of paper files will occur in a manner that obscures data from non-research team members and will always remain in the physical possession of the research team member; electronic data transfer will occur via a password-protected disk or secure transfer protocol.

10.4. Authorization for Use and Disclosure of Protected Health Information (HIPAA)

All subjects will consent, through their IRB-approved informed consent document or HIPAA release, to release of protected health information to the University of Iowa research team as part of the consent process.

10.5. Human Subject Protections

10.5.1. Research Subject Selection

Before implementation, the UI Investigators will review the study protocol with providers in participating clinics. These provider engagement activities will ensure that the involved clinical services approve of the study protocol.

Subjects will have multiple opportunities, both before and during the first visit, to ask questions and read information about the study. After an initial review of the informed consent document, subjects will be given up to 10 days to decide whether they want to participate. No coercion or undue influence on this decision will be used.

There will be no exclusion from participation in the study on the basis of gender, ethnicity, or race. Subjects younger than 50 years of age at the time of screening will be excluded from the study.

10.5.2. Compensation to Subjects

Subjects will be compensated $75 for completion of the baseline visit and $75 for completion of the 12 month visit for their time and inconvenience related to the blood draws and surveys. No compensation will be provided for a subject who does not complete all requirements of a visit. Compensation will be provided by the University of Iowa.

10.5.3. Risks/Discomforts of Study Participation

Subjects might experience one or more of the risks indicated below from being in this study:

- Some of the questions that the SC asks might cause a subject to feel uneasy or anxious, but subjects may choose not to answer any questions.
- Drawing blood for laboratory tests could cause mild and temporary discomfort and might also result in bruising, fainting/lightheadedness and/or infection.
- There is a risk of loss of data confidentiality. To help protect confidentiality, all subject forms completed during study visits will be kept in locked file cabinets in locked offices. The SC will fax data to study researchers at the University of Iowa. Only individuals working on the study will have access to these records. Data will be entered electronically using a unique study ID for each subject. All research data collected will be stored in password-protected computer files that can be accessed only by the subject and the research team. The blood samples that are drawn may be analyzed in the clinic office,

hospital or outside laboratory and therefore viewable by those entities. If study investigators write a report or article about this study or share the study data set with others, individual subjects will not be directly identified.

10.6. IRB Review

If a site relies on their local IRB as the IRB of record, it will approve the protocol and informed consent documents, agree to monitor the conduct of the study, and agree to review study progress periodically, at intervals consistent with applicable federal regulations. The University of Iowa IRB will approve the protocol and informed consent documents for study sites that do not have their own IRBs. The PI or Lead Provider (if relying on local IRB oversight) will be responsible for submitting any and all revisions to the appropriate IRB before implementation of any deviation from the approved protocol. The Lead Provider must provide the CCC with the IRB annual re-approval of the protocol and with all approved versions and revisions to the informed consent documents and recruitment letters or any amendments to the protocol.

The CCC will obtain IRB approval from the University of Iowa IRB (UI IRB) and submit modifications (when needed) and annual continuing review applications. The CCC will notify the UI IRB when an event occurs that is both unanticipated and deemed by the medical monitor to be possibly, probably or definitely related to participation in the study.

The CCC will also track modifications and annual renewals for each participating site.

11. SITE TRAINING AND MONITORING

11.1. Initial Information and Training Sessions

11.1.1. Provider Information Session and Surveys

University of Iowa study investigators will visit each participating study site early in the study and before subject enrollment begins. Investigators will provide clinic providers with a detailed explanation of the study, preliminary activities and study expectations. The information session will be scheduled at a time preferred by the clinic's providers.

The lead provider at each clinic will read the accompanying letter of consent and, if willing, complete the Medical Home Index, a validated, self-assessment tool for evaluating primary care practice. Lead providers will complete this tool before training and again ~30 months later to determine how each office has improved on adoption of the Medical Home and if there is greater adoption in intervention offices compared to controls. We have also pioneered a validated instrument to measure physician-pharmacist collaboration; we will administer this tool to providers in all participating offices, whether intervention or control, at baseline and at the beginning of year 4 of the UI grant funding period to evaluate the level and type of communication between providers and pharmacists.

11.2. Study Coordinator Training

Each clinic will identify 1-2 staff members to serve as the SC. This person(s) will 1) identify and consent subjects, 2) instruct subjects in use of the PHRM, used to provide subjects with a way of documenting their self-monitoring and self-management activities, 3) conduct study procedures at baseline, i.e., at the time of consent, and 12 months later, 4) abstract limited subject medical record data covering the time period that spans two years prior to consent through 30 months following consent and 5) report any serious events such as hospitalizations or emergency department visits as soon as possible once becoming aware of the event. The SC(s) will be trained in study procedures, including completion of CRFs and standardized BP measurement using the Omron monitor, as well as in the use of the PHRM. This training will be conducted during an in-person visit by University of Iowa study team members (preferred mode) or, if necessary, remotely through a webinar. The SC(s) will also receive regular contacts by CCC staff to reinforce the initial training.

11.3. Teleconference Calls

Iowa investigators will schedule periodic conference calls with the Lead Provider and SC on an as needed basis during the first year of the study. In intervention sites, these calls may be scheduled as often as quarterly and then at least once a year thereafter. The purpose of calls to intervention sites will be to solicit input from providers on strategies to optimize communication and to improve the intervention, if necessary. These calls will also be used to ensure fidelity to the intervention. he CCC PI will hold teleconference calls on an as needed basis with the lead provider and SC at any site, intervention or usual care ("control"), that does not meet expectations for recruitment or data collection.

It may be necessary to conduct additional conference calls between the CVRS pharmacists and providers in intervention offices to maintain good communication and team-care. These calls will solicit feedback from providers on how the intervention and communication is working and if they have suggestions for improvement.

11.4. Interim Monitoring Visits

All sites will be monitored by study personnel ("monitors") from the Data Management Center. The purpose of the monitoring visit is to ensure that the protocol is being followed, that subjects' rights and safety are being protected, and to confirm data integrity and quality. The first monitoring visit will occur after 3-5 subjects have been enrolled at a site or approximately three months after the first subject is enrolled, depending on the progress a site is making and/or the challenges they are experiencing. All centers will have a visit approximately 12 months after the first subject is enrolled, a close-out visit after all data are submitted and additional visits as needed for problems.

Study monitors will need access to medical records but will have NO contact with subject subjects. Once study sites have enrolled their first subject, the CCC will send an email to the Lead Provider and SC requesting such access to the office's medical record system ahead of the first monitoring visit.

11.4.1. Pre-Monitoring Procedures

Approximately two weeks ahead of the initial anticipated visit, if possible, and 4-6 weeks ahead of each subsequent monitoring visit, the monitor will email the SC to negotiate and finalize a date for the visit. The SC and Lead Provider should be available to meet with the monitor during the visit.

The monitor will send a letter to the site approximately one week ahead of the initial baseline visit date and 2 weeks ahead of subsequent monitoring visit dates, explaining objectives of the visit and necessary materials. The monitor will need a reserved space in which to work and access to a photocopy machine and electronic records, if applicable. The following items should be available for review:

- Screening Log/Verification of Inclusion and Exclusion Criteria forms
- Patient Clinic Medical records
- Paper copy CRFs and any other study-related source documents and records
- Regulatory Documents
  - IRB approvals (either UI or local IRB)
  - Approved informed consent documents
  - Approved recruitment materials
  - IRB correspondence
  - BP measurement certifications

11.4.2. On-site Monitoring

An initial meeting (approximately 30 minutes) will occur between the SC and the monitor to orient the monitor to clinic/medical records, answer study questions, and review protocol procedures. The SC should be available periodically throughout the visit to answer questions or to make data corrections, if necessary.

In addition to review of the items listed above, the monitor will re-certify the SC in BP measurement procedures on an annual basis.

At the end of the monitoring visit, the monitor will meet briefly with the SC and Lead Provider to discuss findings and a plan of action.

11.4.3. Post-Monitoring

The monitor will send the site a formal report containing feedback and a detailed listing of all findings within approximately four weeks of concluding the monitoring visit.

The monitor will contact the SC to discuss pending items until all items are resolved. The SC will respond to pending items in a timely manner and inform the monitor of any issues delaying resolution of the item. SCs are responsible for maintaining CRFs that reflect accurate data and should indicate any corrections or errors by drawing a single line through the data and initialing and dating as per GCP standards. Corrected CRFs need to be faxed into the CCC after all pending items, identified by the monitor, have been resolved.

11.5. Close-Out Visits

Once all data have been collected from a clinic, a study close out visit will occur. A study monitor from the University of Iowa visit or evaluate remote electronic medical record data at each site at the end of the study to ensure all data are complete. Once all data have been collected, the study monitor will close out that site's participation in the study.

11.6. Organizing and Maintaining Study Files

Each subject should have a study file containing the signed informed consent document, completed CRFs and other source documentation. Subject files should be arranged in order of subject study ID. Within a given subject file, forms should be arranged in the order of study visits.

SCs should retain the Screening Log/Verification Form (see Appendix III) for all patients initially screened, keeping them together in one of two folders: if patient does not meet screening criteria, file in "Eligibility Failure" folder; if patient meets screening criteria, file in "Meets Eligibility" folder. Later, if subject agrees to participate in the study, the SC should transfer Screening Log/Verification Form to subject's individual folder. Remaining Screening Log/Verification Forms, i.e., screening failures (those who were ineligible or others who met criteria but declined participation), should be faxed to the CCC in batch form on a quarterly basis.

11.6.1. Completed paper versions of case report forms (CRFs)

The SC is responsible for completion of all required fields in ink on the CRFs. Do not leave any field blank. If subject refuses a question, the SC should write “refused” on CRF next to item. If an item has been intentionally left blank, i.e., is not applicable, the SC should indicate this by writing a zero with a line through it to show it has not been missed. **If corrections to paper copies are needed, the SC should draw a single line through the incorrect response, write the correct response, and initial and date the correction.** White-Out or other similar products that obscure the original response may not be used on source documents.

11.6.2. Regulatory Documents

All regulatory documents should be kept together in hard-copy form, in a separate binder or folder. These documents include:

1) IRB of record documents
    - All approval letters, modification/amendment submissions, approved and stamped copies of documents (e.g., recruitment materials and informed consent documents), and any correspondence with the IRB.
    - Some IRBs have separate templates for sites to report serious adverse events and protocol deviations. If a site relies on local IRB oversight and it requires such reporting, please include these reports under this tab.

    For sites with local IRB oversight, each IRB document should be stored electronically on a secure computer and emailed to brian-gryzlak@uiowa.edu.

2) ICARE Study tab: Letters and reports related to study monitoring and BP training certifications should be filed here.

11.7. Incorrect Subject Enrollment/Protocol Deviation

If it is determined that an enrolled subject does *not* qualify for the study, the SC must notify Brian Gryzlak at brian-gryzlak@uiowa.edu as soon as possible or within 2 working days after the SC becomes aware of the error.

12. UNANTICIPATED PROBLEMS (UPs)

12.1. Key Definitions

Unanticipated Problems (**UPs**) are considered to be any event or problem that is:

- Unexpected

AND

- Possibly, probably, or definitely related to study participation

AND ONE OR BOTH OF THE FOLLOWING:

- Is fatal, life-threatening or serious

OR

- Suggests greater risk of harm to study subject(s) than was previously known or recognized, including a breach of confidentiality, a subject complaint that cannot be resolved by study investigators, or identification of a new risk related to the study.

Note: *If there is any question concerning classification of an event as a UP, Lead Providers or SCs must contact UI Investigators for a clarification or recommendation.*

12.2. Responsibilities of Involved Parties

The monitoring and reporting of UPs is an important process for ensuring the safety of subjects participating in clinical research.

A decision tree for these problems/events can be found at the end of this section (see Figure 2).

12.2.1. Study site personnel

Lead Providers and research personnel at study sites have primary responsibility for discovery and preliminary evaluation of UPs, and for the submission of UP reports to the University of Iowa (UI) Investigators via fax.

A Lead Provider Investigator who is uncertain about the need to report a specific problem/event should contact UI Investigators for a recommendation.

Detailed instructions for reporting UPs are given later in this section.

Reporting of UPs to IRBs depends on which IRB is overseeing the study at each site:

1. Local site personnel who are overseen by the UI IRB will have their reportable UPs submitted by UI staff members. Local personnel at these sites do not need to contact the UI IRB.
2. Local site personnel who are overseen by a Local IRB should submit reports of UPs to their IRB per their IRB's policy.

Please note that a study monitor from the team of UI investigators will review the medical records of

enrolled subjects to ensure that all UPs have been reported.

12.2.2. Local IRBs

Local IRBs who are overseeing a study site have two responsibilities related to UPs:

1. Review of individual UP cases
    a. The Local IRB receives and reviews reports from Lead Providers regarding on-site UPs involving risks to subjects or others.
    b. The Local IRB has the prerogative to halt any activities at their site that have been associated with unanticipated serious harm to subjects and/or to mandate that new information be added to the informed consent document at their site.
    c. The Local IRB should provide Lead Providers with a formal decision regarding their review of the problem/event and what actions are determined to be necessary.
    d. Lead Providers should then forward the Local IRB decision to University of Iowa Investigators.
2. Annual review of research at the Local Site
    a. Either the UI IRB or the Local IRB will conduct an annual continuing review of research at the site that includes consideration of UPs, interim findings, and any recent literature that may be relevant to the research.
    b. A Local IRB may rely on a current statement from the study's Data and Safety Monitoring Board (DSMB) or sponsor indicating that it has reviewed study-wide UPs, interim findings, and any recent literature that may be relevant to the research, in lieu of requiring that this information be submitted directly to the IRB. Institutions and IRBs may require additional information for continuing review, at their discretion.

12.2.3. University of Iowa IRB

If a site is using the University of Iowa's IRB as their IRB of record, UI research team members will observe the following procedures regarding UP reporting:

1. If UI Investigators receive submitted reports of UPs from Lead Providers, they will forward them to the study's Physician Monitor for review.

2. Members of the research staff will also forward all reportable UPs to the UI IRB per IRB policy.

3. The study Physician Monitor will contact UI Investigators if an expedited UP report to the study funder is required. In these cases:

a. UI Investigators will develop a plan to address the event or problem.

   i. Should UI Investigators determine that a site UP resulted from either improper or deficient procedures or unjustified disease management practices at the site, UI Investigators will request that the site develop a Corrective Action Preventive Action (CAPA) plan.

   ii. UI Investigators, in collaboration with Lead Providers, will revise the plan as needed to optimally decrease the likelihood that the UP will recur.

   iii. UI Investigators and UI Study Monitors will monitor both implementation of the plan and resolution of the identified deficiency through site teleconferences.

b. UI Investigators will submit the expedited UP report to the study funder's Project Officer and send a copy of the report to Lead Providers, to the IRB that is responsible for study oversight at the site and to the study's DSMB.

c. If the event or problem is of a nature that it would affect the entire study, UI Investigators will send the report to all Lead Providers for transmission to their Local IRBs.

4. Should UI Investigators learn of information that is relevant to the ICARE Study or that may impact subjects (e.g. results from another clinical trial), they will communicate an advisory to Lead Providers and SCs through an email listserv. The listserv mechanism is in place to relay any critical information immediately, if necessary.

Figure 2. Decision Tree for Reporting Unanticipated Problems

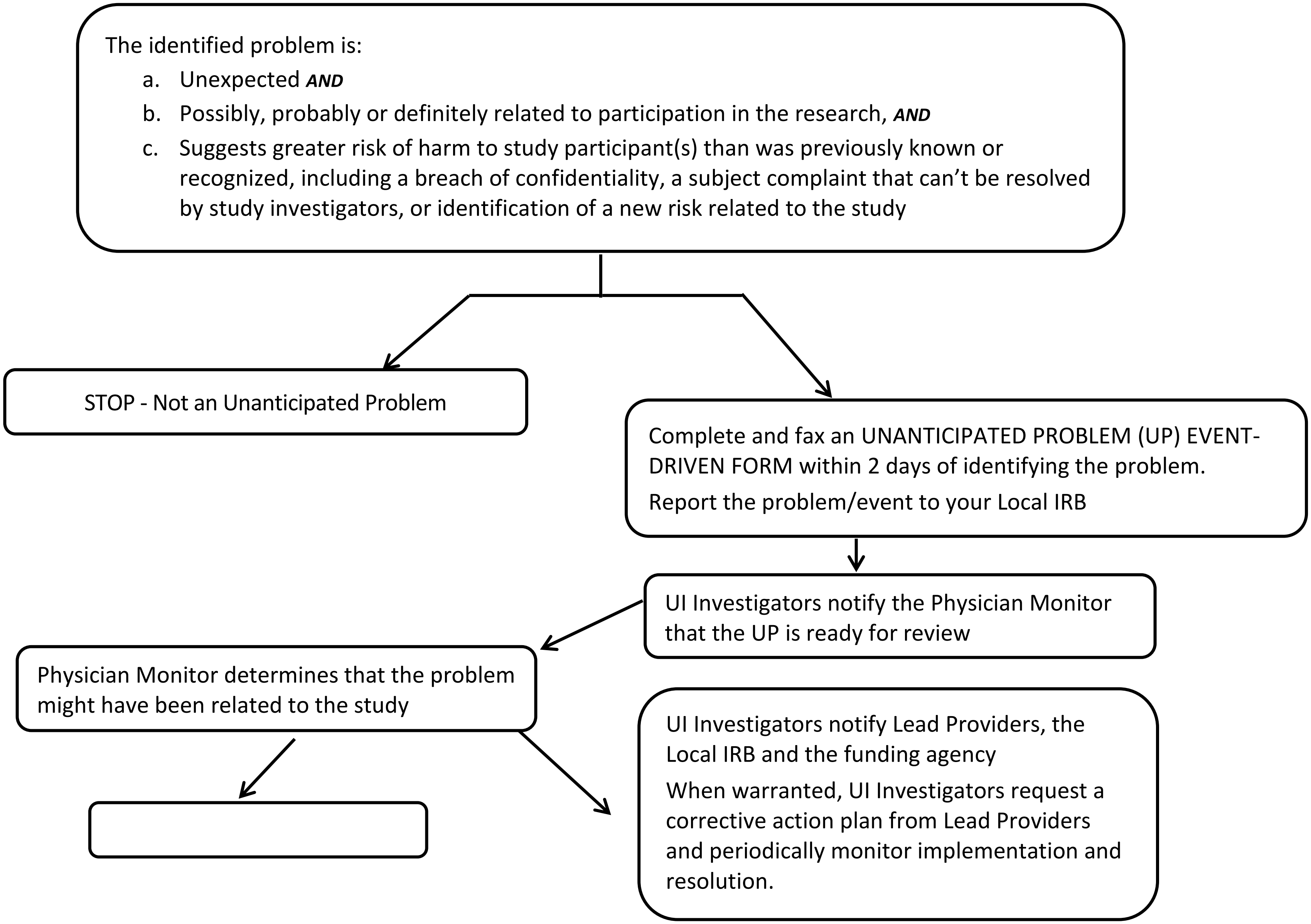

13. PROCEDURES FOR CVRS CLINICAL PHARMACISTS AND SITE PROVIDERS (Intervention sites only)

13.1. Procedures for the CVRS Clinical Pharmacist

13.1.1. Medical Record Data Sources

The CVRS clinical pharmacists will have three main sources of data to support their evaluation of patients and their recommendations to patients and physicians. The primary source of data will be the baseline data collected by the SC and uploaded into the PHRM. Some intervention offices have given the CVRS pharmacists approval to log into their electronic medical records (EMR) remotely. In these offices, the primary source of data for the pharmacists will be the EMR. The second source of data will be the PHRM data entered by patients who utilize the PHRM. The third source of data will be from patient interviews over the telephone. If there are discrepancies in these data, the medical record will be assumed to be the correct source. However, if there are serious discrepancies, the pharmacist will work to resolve these discrepancies whenever possible.

13.2.1 Scheduled Subject Visits

The clinical pharmacist will follow each subject for approximately 12 months following enrollment in the study. Recommended visit activities and frequencies are outlined below:

13.2.1.1 Baseline Visit

The pharmacist may elect to conduct any/all of the following activities at the initial visit, requiring 30-45 minutes:

1. Review the subject's medical record and perform a structured interview, including:
    a. A detailed medication history of all prescription, nonprescription, and herbal therapies
    b. An assessment of subject knowledge of medications, purpose of each medication, goals of therapy, medication dosages and timing, and potential medication side effects
    c. Potential contraindications to specific pharmacologic agents (e.g., renal insufficiency for thiazide diuretics, severe obstructive lung disease for beta blockers)
    d. Expectations that there will be future dosage changes and monitoring and that the pharmacist will discuss issues that might become future barriers to lowering cardiovascular risk (e.g., side effects, non-adherence, subject self-efficacy)
    e. Expectations that, since cardiovascular risk is higher at the initial visit (by definition), medications and/or dosages must be intensified unless there is a strong justification not to intensify them.
2. Provide subjects with lifestyle educational materials (e.g. "Finding Your Way to a Healthier You" brochure and NHLBI's "The Dash Diet" and "Heart Healthy Recipes Cookbook").

3. Utilize motivational interviewing techniques to assess Stages of Change for key issues such as exercise, diet, weight, tobacco use, immunizations and cancer screenings.
4. Encourage the subject to access the PHRM and download a wallet card listing their medications and doses, contact information, and disease states for subjects with memory problems or unintentional non-adherence.
5. Create a care plan with treatment recommendations for the physician or other provider. The care plan will make specific recommendations to improve medication management to achieve lower cardiovascular risk. All recommendations will be informed by the most recent national guidelines for a given condition or drug therapy and based on scientific evidence. If necessary, the pharmacists will present the provider with the primary literature or guideline to support a recommendation.
6. Document all visits, recommendations made to the physician or other provider and recommendations accepted by the physician in the medical record or the pharmacy record, depending on the policies and procedures in the office.
7. Present the care plan via written, verbal, or electronic communication to the provider. Some offices have given the pharmacist authority to pen a drug change or other recommendation in the EMR that can then be quickly approved by the provider and implemented by the pharmacist.
8. Implement the care plan after obtaining provider agreement or provider modifications.

13.2.1.2 Follow-Up Visits

The CVRS model *recommends* structured follow-up visits with the subject at the following time points after the baseline visit. However, the pharmacist may tailor subject visit schedules to meet the individual subject's needs.

- 2 weeks
- 4 weeks
- 6 weeks
- 8 weeks
- Monthly thereafter

Each follow-up visit is estimated to take 30-45 minutes and will include assessment and documentation of:

1. Current medications
2. Side effects and adverse events
3. Subject medication adherence
4. Modification of the care plan as needed, with changes also documented in the subject's medical record
5. Medication changes made or recommend medication changes depending on strategy preferred by the provider
6. Communication with the subject's physician or other provider as needed

14. Subject Termination

Subjects will be terminated:

1) When all study time points have been completed through the 30 month medical record data abstraction

2) When a subject is not able to complete all study time points (early termination). Early termination may occur for several reasons:

    i) Subject's eligibility status changes

        (a) Although pregnancy is not anticipated in study subjects, any subject who becomes pregnant must immediately be terminated

        (2) Subjects who develop a baseline exclusion criterion subsequent to enrollment such as a stroke or advanced cancer will be permitted to stay in the study and should NOT be terminated early

    ii) Subject chooses to withdraw

    iii) Subject is lost to follow-up

    iv) Subject transfers care to another clinic that is not participating in the study

    v) Subject withdraws or is terminated due to an adverse event

    vi) Subject dies

Complete the subject termination form and fax to the CCC for any of the above events.

15. PROCEDURES FOR SITE PROVIDERS

15.1. Provider Surveys

The following surveys will be distributed to providers. These surveys will be distributed at the site training session and should be completed **prior to the beginning of subject enrollment**. The surveys will be administered again at the beginning of year 4 of the UI grant funding period.

<u>Lead Provider only</u>:

- "Medical Home Index – Adult"

<u>All Providers</u>:

- "Managing Cardiovascular Disease States"
- "Physician Collaboration Survey"

As site providers are also considered research subjects in relation to the provider survey, survey packets will include a letter detailing the elements of consent. Return of the survey will constitute consent. Providers who choose to participate should return the survey to the SC sealed in the envelope provided who will then forward it to the CCC via the Study Monitor or other agreed-upon mode.

Providers will be invited to take the same survey at the end of the study.

Providers will not be compensated for completing the study surveys.

15.2. Referring Subjects to the Study

All clinic providers are encouraged to refer subjects who might qualify for the study to the clinic's SC. The SC will review records for each subject referred to make sure that the subject indeed meets the study's complex eligibility criteria.

15.3. Provider Interaction with the CVRS Pharmacist

The CVRS pharmacist will develop an action plan that addresses gaps in preventive health screening or guideline-concordant therapy. The pharmacist will communicate recommended changes in treatment to the patient's primary care provider via fax or other communication method preferred by the individual provider. Communication will occur on the study's Pharmacist-Physician Communication form (see APPENDIX VI). This form is initiated by the study pharmacists, and providers are asked to either agree with the proposed changes to the treatment regimen or modify the pharmacist's proposal and then return the form to the study pharmacist by fax as soon as possible. Pharmacist communication to the provider will occur every 3 months or more frequently if urgent issues are identified.

APPENDIX I: PARTICIPATING SITES

Participating Study Sites	
**Clinic or Practice**	**City, State**
**Akron Mercy Medical Clinic**	Akron, IA
**Burlington Area Family Practice Center**	West Burlington, IA
**Des Moines University Family Medicine Clinic**	Des Moines, IA
**Employee Health Clinic, Mercy Cedar Rapids**	Cedar Rapids, IA
**Great River Medical Group**	Davenport, IA
**Grinnell Regional Family Practice**	Grinnell, IA
**Iowa Specialty Hospital-Belmond Clinic**	Belmond, IA
**Iowa Specialty Hospital-Clarion Clinic**	Clarion, IA
**Knoxville Hospital Clinic**	Knoxville, IA
**Newton Clinic, P.C.**	Newton, IA
**Siouxland Community Health Center**	Sioux City, IA
**University of Iowa Health Care - River Crossing**	Riverside, IA

APPENDIX II: QUICK GUIDE TO ICARE STUDY ELIGIBILITY CRITERIA

ICARE Study Quick Guide to Inclusion and Exclusion Criteria

Section A: Demographic Criteria

1. Patient was seen in your clinic or practice at least once in the past 24 months
2. English-speaking male or female
3. Age is 50 or older at medical record screening

Section B: Risk Factors

4. Has ONE OR MORE of the following:
   - ☐ Diagnosis of diabetes *(ICD9 Code 250)* AND most recent:
     - Hg A1C ≥ 7.5%
   - ☐ Diagnosis of high cholesterol *(ICD9 Code 272)* AND most recent:
     - LDL >110mg/dl for patients with PAD, CAD, Stroke, TIA, or Diabetes OR
     - LDL >140mg/dl
   - ☐ Diagnosis of hypertension *(ICD9 Codes 401, 402, 403, 404, 405)* AND most recent blood pressure:
     - ≥ 150 mm Hg SBP or ≥ 90 mm Hg DBP for patients with uncomplicated hypertension OR
     - ≥ 140 mm Hg SBP for patients with diabetes or chronic kidney disease.

Section C: Cardiovascular Conditions

5. Total number of risk factors in Section B (above) plus number of conditions Section C (below) is THREE OR MORE:
   - ☐ History of coronary artery disease (CAD) *(ICD9 Code 414)*
   - ☐ History of previous MI (heart attack) *(ICD9 Codes 410, 411, 412)*
   - ☐ History of stroke *(ICD9 Codes 430, 431, 432, 433, and 434)*
   - ☐ History of TIA *(ICD9 Code 435)*
   - ☐ History of atrial fibrillation (A. Fib) *(ICD9 Codes 427.31, 427.3)*
   - ☐ History of peripheral vascular disease/claudication (PAD) *(ICD9 Codes 440.2, 440.3, and 440.4)*
   - ☐ History of carotid artery disease *(ICD9 Code 433.1)*
   - ☐ Current smoker *(ICD9 Code 305.1)*
   - ☐ Diagnosis of obesity (BMI>30) *(ICD9 Code 278.0)*

☐ Medical Record screening date: Patient is enrolled WITHIN 6 MONTHS of the date that Sections B, C and C were completed.

Sections D and E: Exclusion Criteria Prior to Consent

6. Has NONE of the following:
   - ☐ Inability to give informed consent
   - ☐ Pregnant
   - ☐ Diagnosis of pulmonary hypertension (Note: secondary pulmonary hypertension is OK)
   - ☐ Cancer diagnosis with a life expectancy estimated less than 2 years
   - ☐ Residence in a nursing home or diagnosis of dementia
   - ☐ No telephone or a hearing impairment not allowing them to use a phone
   - ☐ Refusal to consider attempting to use the internet at home, community center, library, medical office or other source to access the PHRM
   - ☐ Patient has plans to move from the area or transfer care to a different clinic in the next 12 months
   - ☐ Omron blood pressure cuff cannot be used on patient's arm for any reason

APPENDIX III: SCREENING LOG AND VERIFICATION OF INCLUSION AND EXCLUSION CRITERIA FORM

Patient Name:	MRN:	Phone number:

Do not send information in the row above to the University of Iowa. Please copy this form and redact or cut out this info before sending.

SCREENING LOG AND VERIFICATION OF INCLUSION AND EXCLUSION CRITERIA

INSTRUCTIONS *** = Required for all screened patients*

- Please record screening data below for any patient whose chart you have accessed for the ICARE study.
- Patients that you identify as potentially eligible for the study should be sent a recruitment letter and postcard.
- If you have questions on this form contact Brian at 319-335-8218 or brian-gryzlak@uiowa.edu

Screening Number: *(Write on postcard)*

Screening Number (Write number on postcard):

*Gender	❒Male ❒Female	*Race (Check all that apply): ❒ White ❒ Black/African-American. ❒ Asian ❒ American Indian/Alaska Native ❒ Native Hawaiian/Other Pacific Islander	*Outcome: ❒ Patient enrolled in study ❒ Patient ineligible for study ❒ Patient declined via postcard ❒ Patient declined via phone ❒ Patient declined in person ❒ Unable to reach via mail ❒ Unable to reach via phone ❒ Other outcome:
*Ethnicity	❒Hispanic ❒Not Hispanic		
Date letter and postcard mailed:	__ __/__ __/__ __ __ __ ❒n/a		
Date postcard returned:	__ __/__ __/__ __ __ __ ❒n/a		

Phone contact dates and notes (up to 3 attempts):

Section A: Demographic Criteria

**1.**	Has the patient been seen in your clinic or practice at least once in the past 24 months?	❒ YES ➔ **Continue to #2**<br>❒ NO ➔ **STOP – not eligible**
**2.**	Is the patient an English-speaking male or female?	❒ YES ➔ **Continue to #3**<br>❒ NO ➔ **STOP - not eligible**
**3.**	Age of patient in years:	AGE:_______ **IF >= 50, go to #4a. If less than 50, STOP – not eligible.**

**Section B: Risk Factors**			**Meets Criteria ?**
**4a**. Has a diagnosis of diabetes? *(ICD9 Code 250)*	❒ YES ➔ **Continue to #4b**<br>❒ NO ➔ **Skip to #5a**	If 4a is YES AND 4b is >= 7.5%, check box at right.	❒
**4b**. Enter most recent Hg A1c from chart **IF** ≥ 7.5%:	Hg A1c:______% Date: __ __ / __ __ / __ __ __ __		
**5a**. Has a diagnosis of high cholesterol? *(ICD9 Code 272)*	❒ YES ➔ **Continue to #5b**<br>❒ NO ➔ **Skip to #6a**	If 5a is YES AND (5b is >110mg/dl for patients with PAD, CAD, Stroke, TIA, or Diabetes **OR** 5b >140mg/dl), check box at right.	❒
**5b**. Enter most recent LDL from chart **IF**:<br>▪ >110mg/dl for patients with PAD, CAD, Stroke, TIA, or Diabetes **OR**<br>▪ >140mg/dl	LDL: _______mg/dl Date: __ __ / __ __ / __ __ __ __		
**6a**. Has a diagnosis of hypertension? *(ICD9 Codes 401, 402, 403, 404, 405)*	❒ YES ➔ **Continue to #6b**<br>❒ NO ➔ **Skip to #7**	If 6a is YES AND (6b ≥150 mm Hg SBP or > 90 mm Hg DBP for patients with uncomplicated hypertension **OR** 6b ≥ 140 mm Hg SBP for patients with diabetes or chronic kidney disease), check box at right.	❒
**6b**. Enter most recent blood pressure from chart **IF**:<br>▪ ≥150 mm Hg SBP or ≥ 90 mm Hg DBP for patients with uncomplicated hypertension **OR**<br>▪ ≥140 mm Hg SBP for patients with diabetes or chronic kidney disease	__________(SBP)<br>__________(DBP) Date: __ __ / __ __ / __ __ __ __		
**7. Section B Total:** In box at the right, add the number of checked boxes above for **Questions 4, 5 and 6.** ➔<br>**If 1 or more, CONTINUE to Section C. If 0, STOP - not eligible.**			☐

IF PATIENT WAS ENROLLED in the study (signed consent), file a copy of this form in the patient's study folder. ***v.9/19/14*** ***Site:*** ***Screening Number***
IF PATIENT WAS NOT ELIGIBLE or NOT ENROLLED IN THE STUDY, send a copy of this form via fax, mail or email to the University of Iowa.

Section C: Cardiovascular Conditions *(ICD9 Codes)*	Check if meets inclusion criterion
**8.** History of coronary artery disease (CAD) *(ICD9 Code 414)*	❑
**9.** History of previous MI (heart attack) *(ICD9 Codes 410, 411, 412)*	❑
**10.** History of stroke *(ICD9 Codes 430, 431, 432, 433, and 434)*	❑
**11.** History of TIA *(ICD9 Code 435)*	❑
**12.** History of atrial fibrillation (A. Fib) *(ICD9 Codes 427.31, 427.3)*	❑
**13.** History of peripheral vascular disease/claudication (PAD) *(ICD9 Codes 440.2, 440.3, and 440.4)*	❑
**14.** History of carotid artery disease *(ICD9 Code 433.1)*	❑
**15.** Current smoker *(ICD9 Code 305.1)*	❑
**16.** Diagnosis of obesity (BMI≥30) *(ICD9 Code 278.0)* Enter most recent BMI >= 30 from chart: _________ Date: ___ ___ / ___ ___ / ___ ___ ___ ___	❑
**17.** **Section C Total**: In box at the right, add the number of checked boxes for **Questions 8-16➔**	☐
**18.** Add the answers for #7 & #17. **If 3 or more, CONTINUE to # 19. If less than 3, STOP – not eligible ➔**	☐

Section D: Exclusion Criteria *(from Medical Record, Direct Observation, OR Self-Report)*	Check if meets exclusion criterion
**19.** Inability to give informed consent - *direct observation OK*	❑
**20.** Pregnant *(ICD9 Codes V22, V23, V24)*	❑
**21.** Diagnosis of pulmonary hypertension (*ICD9 Code 416; Note: secondary pulmonary hypertension is OK)*	❑
**22.** Cancer diagnosis with a life expectancy estimated less than 2 years	❑
**23.** Residence in a nursing home or diagnosis of dementia –*Self-report OK for N.H. residence*	❑
**24.** No telephone or have a hearing impairment not allowing them to use a phone – *Direct observation and self-report OK*	❑
**25.** Omron blood pressure cuff cannot be used on patient's arm for any reason – *direct observation OK* (e.g., patient is morbidly obese and requires use of a thigh cuff)	❑
**26.** **Section D total:** In box at the right, add the number of checked boxes for **Questions 19-25➔** **If 0, CONTINUE to Question 27. If 1 or more, STOP– not eligible**	☐

27. Subject is potentially eligible based on your chart review and can be sent a recruitment letter and postcard or otherwise considered for enrollment.
At the right, enter the date that the chart review for Sections B, C, and D was completed. Then continue to Question 28 in Section E.
Date: __ __ / __ __ / __ __ __ __

Section E: Exclusion Criteria from Patient Self-Report *(Ask during your contact to schedule the baseline visit)*	Check if meets exclusion criterion
**28.** Refusal to consider attempting to use the internet to access the PHRM	❑
**29.** Patient has plans to move from the area or transfer care to a different clinic in the next 12 months	❑
**30.** **Section E total:** In box at the right, add the number of checked boxes for Questions 28-29➔ **If 0, CONTINUE to Question 31. If 1 or more, STOP– not eligible**	☐

**31. Before the subject signs consent, verify that all are correct:**	❒ ANSWER TO QUESTION 18 IS 3 OR MORE.<br>❒ DATE IN QUESTION 27 IS LESS THAN 6 MONTHS FROM THE DATE THAT THE PATIENT WOULD SIGN THE CONSENT DOCUMENT (#32 BELOW). *If the date in Question 27 is MORE THAN 6 months from the date the consent would be signed, rescreen the patient for eligibility with a new screening log using current medical record data.*<br>❒ PATIENT HAS NONE OF THE EXCLUSION CRITERIA BASED ON MEDICAL RECORD, DIRECT OBSERVATION, OR PATIENT REPORT
**32.** Subject may sign consent. Enter date subject signed:	Date: ___ ___ / ___ ___ / ___ ___ ___ ___

IF PATIENT WAS ENROLLED in the study (signed consent), file a copy of this form in the patient's study folder. ***v.9/19/14*** ***Site:*** ***Screening Number***
IF PATIENT WAS NOT ELIGIBLE or NOT ENROLLED IN THE STUDY, send a copy of this form via fax, mail or email to the University of Iowa and file in "Eligibility Failure" folder.

APPENDIX IV: BASELINE VISIT CHECKLIST

ICARE
Improved Cardiovascular Risk Reduction to Enhance Rural Primary Care

ICARE Study Subject Enrollment and Baseline Visit Checklist

(Complete for eligible patients only – check each activity below when completed)

Before Visit

- ❒ Gender, race, and ethnicity questions of the *Screening Log and Verification of Inclusion and Exclusion Criteria ("Screening Log / Verification")* form completed
- ❒ Sections A, B, C, D and E of the *Screening Log / Verification* form completed

During Visit

- ❒ Sections D and E of *Screening Log / Verification* are reviewed with patient to confirm that patient has none of the exclusion criteria, and *Screening Log / Verification* form is completed
- ❒ Current, stamped Informed Consent Document reviewed with patient and questions answered
- ❒ Patient signed Informed Consent Document
- ❒ Copies made of all pages of signed informed consent document
    - ❒ Original signed and dated Informed Consent Document (all pages) filed in patient's folder
    - ❒ Copy of signed and dated Informed Consent Document given to patient
    - ❒ Copy of signed and dated Informed Consent Document filed in the patient's medical record *(If applicable per your site's policies)*
- ❒ Labs (HbA1c; lipids, preferably fasting) drawn
- ❒ Blood pressure measured using Omron machine (3 or 4 measurements as needed)
- ❒ Patient instructed on PHRM
- ❒ Patient given PHRM login sheet

All 5 baseline forms administered and completed:

- ❒ *Enrollment*
- ❒ *Diagnosed Conditions and Care Management (patient-reported questions)*
- ❒ *Medication Reconciliation (patient-reported questions)*
- ❒ *Blood Pressure, Laboratory And Cancer Screening Form*
- ❒ *Health Behavior Inventory*

During Visit or Directly After Visit

- ❒ Remaining questions on *Diagnosed Conditions and Care Management* and *Medication Reconciliation* forms that require the medical record to answer are completed.
- ❒ All 5 baseline forms faxed to the Clinical Coordinating Center (CCC) within 48 hours of the visit.
- ❒ *Screening Log / Verification* form faxed to the CCC
- ❒ *Screening Log / Verification* form filed in patient's study folder.
- ❒ All 5 baseline forms are filed in the patient's study folder.

APPENDIX V: CASE REPORT FORMS

Study ID: _ _-_ _

I-CARE
Improved Cardiovascular Risk Reduction
to Enhance Rural Primary Care

Enrollment Form

Baseline Only

Patient's Name: ______________________________ *(last)* ____________________________ *(first)*

Date Consented: _ _ / _ _ / _ _ _ _ (mm/dd/yyyy)

Date Administered: _ _ / _ _ / _ _ _ _ (mm/dd/yyyy)

Primary Provider: ____

Primary Provider Phone: _______ - _______ - ___________

Primary Provider Fax: _______ - _______ - ___________

<u>Section I: Contact Information</u>

Address:

_______________________________________ *(street)*

_______________________________________ *(city, state)*

_____________ *(zip code)*

Phone Numbers:

- Home: _______ - _______ - ___________
- Cell: _______ - _______ - ___________
    - Text Messaging: **Yes** **No**
- Work: _______ - _______ - ___________

Email: ____

Preferred Contact *(circle all that apply)*: **Home** **Cell** **Work** **Email**

Study ID: _ _-_ _

Section I: Contact Information *(continued)*

Alternative Contact:

- Name: ______________________________________
- Relationship: _________________________________
- Phone: ______ - ______ - __________

Section II: Demographics

INSTRUCTIONS (to be read to the subject):

"The first questions ask for some basic information about you."
(Study Coordinator is to check the box corresponding to the subject's answers.)

Birthdate: "What is your date of birth?" _ _ / _ _ / _ _ _ _ (mm/dd/yyyy)

Gender: ☐M ☐F

I. Patient Race "Please tell me which of the following racial groups best represent you." (check all that apply)

☐ Black or African American
☐ American Indian or Alaska native
☐ Native Hawaiian or Other Pacific Islander
☐ Asian
☐ White or Caucasian
☐ Unknown/Not Reported

II. Patient Ethnicity "Please tell me which of the following ethnic groups best represent you."

☐ Hispanic/Latino
☐ Non-Hispanic/Non-Latino
☐ Unknown/Not Reported

III. Education "Please tell me the highest grade you completed or the highest degree you have received." (Check only one):

☐$_1$ 1- 5
☐$_2$ 6- 8
☐$_3$ 9- 12
☐$_4$ 2-year technical or associate degree
☐$_5$ 4-year BA or BS degree
☐$_6$ Masters degree
☐$_7$ Doctoral degree

Study ID: _ _-_

Section II: Demographics *(continued)*

IV. Insurance Status "Please tell me what kind of insurance is the primary payer for your healthcare." (Please check only one, the primary insurer.)

☐1 Private insurance (Employer/Group)
☐2 Private insurance (Self-insured)
☐3 Medicare
☐4 Medicaid
☐5 None/Self-pay
☐6 Free care

V. Insurance Coverage for Prescriptions "Do you have insurance coverage for prescriptions?"

☐1 Yes
☐0 No

VI. Annual Household Income "Can you please tell me which category best represents your total annual household income?"

☐1 <$10,000
☐2 $10, 000-$24, 999
☐3 $25,000- $39,999
☐4 $40,000-$54,999
☐5 $55,000-$79,999
☐6 $80,000-$99,999
☐7 $100,000 or greater
☐8 Refused to answer

VII. Marital Status "Can you please tell me which category best represents your current marital status?"

☐1 Never married
☐2 Married
☐3 Divorced or separated
☐4 Widowed

VIII. Smoking Status "Have you ever smoked? If so, are you currently smoking or are you an ex-smoker?"

☐0 Never smoked

☐1 Current smoker

"If you are currently smoking, please tell me the total number of years you have smoked and the approximate number of cigarettes that you smoke each day."

Number of years smoked: __ __

Number of cigarettes smoked per day: __ __

Study ID: _ _-_

<u>Section II: Demographics</u> *(continued)*

☐$_2$ Ex-smoker "If you are an ex-smoker, how many years ago did you quit? Also, how many years did you smoke and approximately how many cigarettes did you smoke each day?"

Years since quit:

- ☐$_1$ < 5 years
- ☐$_2$ 5-14 years
- ☐$_3$ $\geq$ 15 years

Number of years smoked: __ __

Number of cigarettes smoked per day: __ __

Study ID: «StudyIDs»

Diagnosed Conditions and Care Management
Baseline Visit

Section A. Patient-Reported Conditions and Care Management

1. Date administered: ___ ___ / ___ ___ / ___ ___ ___ ___ (MM/DD/YYYY)

Ask the subject whether they have each of the following conditions.

**"Please tell me if you have ever had any of the following medical conditions. Have you ever had …."** ***Answers to all questions are required.***	**Patient Response**	
	**YES**	**NO**
**2.** Hypertension or high blood pressure?	❒	❒
**3.** Hyperlipidemia or high cholesterol?	❒	❒
**4.** Congestive heart failure?	❒	❒
**5.** Coronary artery disease?	❒	❒
**6.** Atrial fibrillation or A.Fib?	❒	❒
**7.** Heart attack?	❒	❒
**8.** Stroke or TIA?	❒	❒
**9.** Peripheral artery disease?	❒	❒
**10.** Asthma? (excluding: exercise induced asthma)	❒	❒
**11.** COPD?	❒	❒
**12.** Diabetes?	❒	❒
**13.** Chronic kidney disease?	❒	❒
**14.** Seizures or other neurological disorder?	❒	❒
**15.** Liver disease?	❒	❒
**16.** Depression?	❒	❒
**17.** Anxiety?	❒	❒
**18.** Arthritis, Degenerative joint disease, or chronic pain?	❒	❒

Study ID: «StudyIDs»

**Ask the patient the following questions:**	**YES**	**NO**
**19.** "Are you free of chest pain?"	❒	❒
**20.** "Have you ever experienced an acute myocardial infarction (heart attack), coronary artery bypass graft (CABG) surgery, a percutaneous coronary intervention (PCI), cardiac valve surgery, or cardiac transplantation in the past 12 months? **OR** do you have chronic stable angina?"	❒	❒
**21.** "Have you participated in a cardiac rehabilitation program?"	❒	❒
**22.** "Have you ever been referred to such a program?	❒	❒
**23.** "Have you received a dilated eye exam in the past 12 months?	❒	❒
**24.** "Have you received a foot examination in the past 12 months?"	❒	❒
**25.** "Have you received a pneumonia immunization?"	❒	❒
**26.** "Do you use tobacco?"	❒	❒
**27.** "Are you currently using nicotine replacement (patch, gum, lozenge, inhaler), buproprion, or Chantix® (varenicline)?"	❒	❒
**28.** "Have you received an influenza immunization during the most recent flu season (September-February)?"	❒	❒

**Study coordinator should answer these questions. Review the patient-reported medications from the Medication Reconciliation form you completed and answer the following questions:**	**YES**	**NO**
**29.** "Is the patient prescribed at least two anti-anginal medications (Drug Codes: 200s, 400s, 900s, or Ranolazine)?"	❒	❒
**30.** Is the patient currently on anticoagulation (Drug Codes: 5001, 5003, 5004, 5201, 5202, 5301, 5401)?	❒	❒

Study ID: «StudyIDs»

<u>Section B. Medical Record-Reported Conditions and Care Management</u>

For each question below, check "YES" if the condition is documented in the patient's medical record and "NO / NOT PRESENT" if it is not. *Answers to all main questions (e.g., 1, 2, 3, …) are required.*	Answer from the Medical Record	
	YES	NO / NOT PRESENT
1. HYPERTENSION?	❒	❒
2. HYPERLIPIDEMIA?	❒	❒
3. CONGESTIVE HEART FAILURE?	❒	❒
4. CORONARY ARTERY DISEASE?	❒	❒
5. ATRIAL FIBRILLATION?	❒	❒
6. HEART ATTACK (myocardial infarction)?	❒	❒
7. STROKE OR TIA?	❒	❒
8. PERIPHERAL ARTERY DISEASE?	❒	❒
9. ASTHMA? *(Excluding exercise induced asthma)*	❒	❒
10. DIABETES?	❒	❒
11. CHRONIC KIDNEY DISEASE?	❒	❒
12. SEIZURES/OTHER NEUROLOGICAL DISORDER?	❒	❒
13. LIVER DISEASE?	❒	❒
14. DEPRESSION?	❒	❒
15. ANXIETY?	❒	❒
16. ARTHRITIS/DJD/CHRONIC PAIN?	❒	❒

Study ID: «StudyIDs»

**For each question below, answer the question using the medical record or check "YES" if the answer is documented in the patient's medical record and "NO / NOT PRESENT" if it is not.** *Answers to all main questions (e.g., 1, 2, 3, …) are required.*	**Answer from the Medical Record**
**17.** Most recent chart recorded blood pressure	___ ___ ___ / ___ ___ ___ mmHg
**18.** Date of most recent chart recorded blood pressure	___ ___ / ___ ___ / ___ ___ ___ ___ *(MM/DD/YYYY)*
**19.** Is there a documented Ejection Fraction (EF) in the chart?	❒ YES ➔ ***Go to 19a***<br>❒ NO / NOT PRESENT ➔ ***Skip to 20***
**19a.** Most recent chart recorded EF:	____ ____ %
**19b.** EF date:	___ ___ / ___ ___ / ___ ___ ___ ___ *(MM/DD/YYYY)*
**20.** Is there documentation the provider asked the patient about dyspnea (shortness of breath)?	❒ YES<br>❒ NO / NOT PRESENT
**21.** Is there documentation the provider asked the patient about chest pain?	❒ YES<br>❒ NO / NOT PRESENT
**22.** Is the patient angina-free (free of chest pain)?	❒ YES<br>❒ NO / NOT PRESENT
**23.** Is the patient prescribed at least two anti-anginal medications (Drug Codes: 200s, 400s, 900s, or Ranolazine)?	❒ YES<br>❒ NO / NOT PRESENT
**24.** Has the patient experienced an acute myocardial infarction (MI), coronary artery bypass graft (CABG) surgery, a percutaneous coronary intervention (PCI), cardiac valve surgery, or cardiac transplantation in the past 12 months?<br>**OR**<br>does the patient have chronic stable angina (CSA)?	❒ YES<br>❒ NO / NOT PRESENT
**25.** Has the patient participated in an early outpatient cardiac rehabilitation/secondary prevention (CR) program for the qualifying event/diagnosis?	❒ YES<br>❒ NO / NOT PRESENT
**26.** Has the patient been referred to such a program?	❒ YES<br>❒ NO / NOT PRESENT

Study ID: «StudyIDs»

For each question below, answer the question using the medical record or check "YES" if the answer <u>is documented in the patient's medical record</u> and "NO / NOT PRESENT" if it is not. *Answers to all main questions (e.g., 1, 2, 3, …) are required.*	Answer from the Medical Record
**27.** Is the patient currently on anticoagulation (Drug Codes: 5001, 5003, 5004, 5201, 5202, 5301, 5401) **OR** has the patient been assessed for the need of anticoagulation?	❒ YES<br>❒ NO / NOT PRESENT
**28.** If the patient is on warfarin, has an INR been recorded an average of at least every 2 months (6 times) for the last year?	❒ YES<br>❒ NO / NOT PRESENT<br>❒ NOT ON WARFARIN
**29.** Has the patient received a dilated eye exam in the past 12 months?	❒ YES<br>❒ NO / NOT PRESENT
**30.** Has the patient received urine protein screening (microalbumin laboratory value) in the past 12 months?	❒ YES ➔ ***Go to 30a***<br>❒ NO / NOT PRESENT ➔ ***Skip to 31***
**30a.** Microalbumin value:	________ mg/g
**31.** Have they received an HbA1c test in the past 12 months?	❒ YES ➔ ***Go to 31a***<br>❒ NO / NOT PRESENT ➔ ***Skip to 32***
**31a.** HbA1c value:	_____ . _____ %
**32.** Have they received an LDL cholesterol test in the past 12 months?	❒ YES ➔ ***Go to 32a***<br>❒ NO / NOT PRESENT ➔ ***Go to 33***
**32a.** LDL value:	__________ mg/dL
**33.** Have they received a foot examination in the past 12 months?	❒ YES<br>❒ NO / NOT PRESENT
**34.** Has the patient received a pneumonia immunization?	❒ YES<br>❒ NO / NOT PRESENT
**35.** Is a plan on how to achieve or maintain ideal body weight within the past 6 months documented in the patient's MR? (Can include 'lifestyle modification' plans, including diet and exercise)	❒ YES<br>❒ NO / NOT PRESENT
**36.** Does the patient use tobacco?	❒ YES<br>❒ NO / NOT PRESENT

Study ID: «StudyIDs»

**For each question below, answer the question using the medical record or check "YES" if the answer is documented in the patient's medical record and "NO / NOT PRESENT" if it is not.** *Answers to all main questions (e.g., 1, 2, 3, …) are required.*	**Answer from the Medical Record**
**37.** Is there documentation in the MR of assessing tobacco use?	❒ YES<br>❒ NO / NOT PRESENT
**38.** Is there documentation in the MR of advising on the risk of tobacco use?	❒ YES<br>❒ NO / NOT PRESENT
**39.** Is there documentation in the MR of assessing the willingness to quit?	❒ YES<br>❒ NO / NOT PRESENT
**40.** Is the patient currently using nicotine replacement (patch, gum, lozenge, inhaler), buproprion, or Chantix® (varenicline)?	❒ YES<br>❒ NO / NOT PRESENT
**41.** Is there documentation of tobacco screening in the MR?	❒ YES ➔ ***Go to 41a***<br>❒ NO / NOT PRESENT ➔ ***Skip to 42***
**41a.** Date of most recent screening	___ ___ / ___ ___ / ___ ___ ___ ___<br>*(MM/DD/YYYY)*
**42.** Is there documentation in the Medical Record that the patient has been asked how much alcohol they drink at least once in the previous 24 months?	❒ YES<br>❒ NO / NOT PRESENT
**43.** Has the patient received an influenza immunization during the most recent flu season (September-February)?	❒ YES<br>❒ NO / NOT PRESENT
**44.** Has the patient received a pneumonia immunization?	❒ YES<br>❒ NO / NOT PRESENT

Study ID: _ _-_ _

I-CARE Improved Cardiovascular Risk Reduction to Enhance Rural Primary Care

Medication Reconciliation

Study Visit: ○ Baseline ○ 12 months **Date Administered:** _ _ / _ _ / _ _ _ _ (mm/dd/yyyy)

List all drug allergies: ______________________________ or check ☐ No allergies

1. List medications that are either a) documented in the **patient's medical record** prior to patient visit AND/OR b) reported by the patient during the study visit. ONLY include **antihypertensive agents**, **hyperglycemic agents**, **cholesterol agents**, **asthma agents** and **anticoagulants/antiplatelet agents**

INSTRUCTIONS (to be read to the subject): "Please tell me what medications you are taking for **high blood pressure, high cholesterol, high blood sugars, asthma or thinning your blood**." Ask about missed doses in the past week and how well it works.

	Medication Name & Code	In the EMR	EMR Strength	EMR Directions for Use	Reported by Patient	Patient Reported Strength	Patient Reported Directions for Use	# Doses Missed in Past Week	How well does it work?
1.	____________<br>**Code:** __ __ __ __	☐ Yes<br>☐ No			☐ Yes<br>☐ No				**Well Okay Not Well**
2.	____________<br>**Code:** __ __ __ __	☐ Yes<br>☐ No			☐ Yes<br>☐ No				**Well Okay Not Well**
3.	____________<br>**Code:** __ __ __ __	☐ Yes<br>☐ No			☐ Yes<br>☐ No				**Well Okay Not Well**
4.	____________<br>**Code:** __ __ __ __	☐ Yes<br>☐ No			☐ Yes<br>☐ No				**Well Okay Not Well**
5.	____________<br>**Code:** __ __ __ __	☐ Yes<br>☐ No			☐ Yes<br>☐ No				**Well Okay Not Well**
6.	____________<br>**Code:** __ __ __ __	☐ Yes<br>☐ No			☐ Yes<br>☐ No				**Well Okay Not Well**

Study ID: _ _-_ _

	Medication Name & Code	In the EMR	EMR Strength	EMR Directions for Use	Reported by Patient	Patient Reported Strength	Patient Reported Directions for Use	# Doses Missed in Past Week	How well does it work?
7.	___________________<br>**Code:** __ __ __ __	☐ Yes<br>☐ No			☐ Yes<br>☐ No				**Well Okay Not Well**
8.	___________________<br>**Code:** __ __ __ __	☐ Yes<br>☐ No			☐ Yes<br>☐ No				**Well Okay Not Well**
9.	___________________<br>**Code:** __ __ __ __	☐ Yes<br>☐ No			☐ Yes<br>☐ No				**Well Okay Not Well**
10.	___________________<br>**Code:** __ __ __ __	☐ Yes<br>☐ No			☐ Yes<br>☐ No				**Well Okay Not Well**
11.	___________________<br>**Code:** __ __ __ __	☐ Yes<br>☐ No			☐ Yes<br>☐ No				**Well Okay Not Well**
12.	___________________<br>**Code:** __ __ __ __	☐ Yes<br>☐ No			☐ Yes<br>☐ No				**Well Okay Not Well**
13.	___________________<br>**Code:** __ __ __ __	☐ Yes<br>☐ No			☐ Yes<br>☐ No				**Well Okay Not Well**
14.	___________________<br>**Code:** __ __ __ __	☐ Yes<br>☐ No			☐ Yes<br>☐ No				**Well Okay Not Well**
15.	___________________<br>**Code:** __ __ __ __	☐ Yes<br>☐ No			☐ Yes<br>☐ No				**Well Okay Not Well**
16.	___________________<br>**Code:** __ __ __ __	☐ Yes<br>☐ No			☐ Yes<br>☐ No				**Well Okay Not Well**

Study ID: _ _-_ _

2. INSTRUCTIONS (to be read to the subject): "Do any of these medications **bother you** in any way?" **YES** _____ **NO** _____

i) If YES, please fill out the following for each bothersome medication, asking the subject "**how much** does it bother you?"

Medication Name	A Lot	Some	A Little	In what way does it bother you?
	☐	☐	☐	
	☐	☐	☐	
	☐	☐	☐	
	☐	☐	☐	
	☐	☐	☐	
	☐	☐	☐	
	☐	☐	☐	

3. INSTRUCTIONS (to be read to the subject): "I have a list of problems that people sometimes have with their medications. Please tell me **how hard** it is for you to do each of the following."

Problems	Very Hard	Somewhat Hard	Not Hard at all	Which Medication? ("All" or specify)
Open or close the medicine bottle	☐	☐	☐	
Read the print on the bottle	☐	☐	☐	
Remember to take all of the pills	☐	☐	☐	
Get your refills on time	☐	☐	☐	
Take so many pills at the same time	☐	☐	☐	

*Adapted from: Svarstad BL, Chewning BA, Sleath BL, Claesson C. The Brief Medication Questionnaire: a tool for screening patient adherence and barriers to adherence. *Patient Educ Couns.* Jun 1999;37(2):113-124

Study ID: _ _-_ _

ICARE
Improved Cardiovascular Risk Reduction to Enhance Rural Primary Care

HEALTH BEHAVIOR INVENTORY

(baseline only)

Date Administered: _ _ / _ _ / _ _ _ _ (mm/dd/yyyy)

	**Strongly Disagree**	**Moderately Disagree**	**Agree Somewhat**	**Moderately Agree**	**Strongly Agree**	**Refused to Answer**
If I get sick, it is my own behavior which determines how soon I get well again.	Strongly Disagree	Moderately Disagree	Agree Somewhat	Moderately Agree	Strongly Agree	Refused to Answer
I am in control of my health.	Strongly Disagree	Moderately Disagree	Agree Somewhat	Moderately Agree	Strongly Agree	Refused to Answer
When I get sick I am to blame.	Strongly Disagree	Moderately Disagree	Agree Somewhat	Moderately Agree	Strongly Agree	Refused to Answer
The main thing which affects my health is what I myself do.	Strongly Disagree	Moderately Disagree	Agree Somewhat	Moderately Agree	Strongly Agree	Refused to Answer
If I take care of myself, I can avoid illness.	Strongly Disagree	Moderately Disagree	Agree Somewhat	Moderately Agree	Strongly Agree	Refused to Answer
If I take the right actions, I can stay healthy.	Strongly Disagree	Moderately Disagree	Agree Somewhat	Moderately Agree	Strongly Agree	Refused to Answer

Adopted from: Wallston, K. A., Wallston, B. S. & DeVellis, R. (1978). Development of the multidimensional health locus of control (MHLC) scales. Health Education Monographs, 6, 160-17

ICARE
Improved Cardiovascular Risk Reduction to Enhance Rural Primary Care

Blood Pressure, Laboratory and Cancer Screening Form

**Study ID: 23-01**	**Visit Date:** *(mm/dd/yyyy)* __ __ / __ __ / __ __ __ __
**Height:** __ feet __ __ inches *or* __ __ __ cm	**Weight:** (lbs or kg) __ __ __ lbs *or* __ __ __ kg
**Visit** *(check one)*: ☐ **Baseline** ☐ **12 months**	

Has the patient smoked in the past 24 months? ☐ Yes ☐ No
If yes, patient's last cigarette was smoked: ☐ > 20 minutes ago ☐ ≤ 20 minutes ago
Delay BP measurement until > 20 minutes has elapsed since patient last smoked.

Time of day of BP recording .... __ __ : __ __ ☐ am ☐ pm

Midpoint circumference of arm being used (right is preferred)................ __ __ cm

Size of cuff used (check one): ☐$_1$ Adult (22-32 cm) ☐$_2$ Small adult (17-22 cm) ☐$_3$ Large adult (32-42 cm) ☐$_4$ Extra Large (42-50 cm)

1. **Seated pulse** (count beats per minute for 30 seconds and multiply by 2)	__ __ __ BPM

	a. Systolic BP (mm Hg)	b. Diastolic BP (mm Hg)
2. **First sitting BP measurement**	___ ___ ___	___ ___ ___
3. **Second sitting BP measurement**	___ ___ ___	___ ___ ___
4. **Third sitting BP measurement**	___ ___ ___	___ ___ ___
**Important➔**	*If the 2nd OR 3rd systolic OR diastolic BPs above differ by more than 4 mm, then take a fourth reading and enter the values below.*	
5. **Fourth sitting BP measurement**	___ ___ ___	___ ___ ___

Have the patient stand quietly for 1 minute and measure the following:

6. **Standing pulse** (count beats per minute for 30 seconds and multiply by 2)	__ __ __ BPM

	a. Systolic BP (mm Hg)	b. Diastolic BP (mm Hg)
7. **Standing BP measurement**	___ ___ ___	___ ___ ___

2014-09-23

Study ID: 23-01

Draw blood and record cholesterol and HA1c values as soon as results are obtained:

8. **Total Cholesterol**	___ ___ ___ mg/dl
9. **High-density lipoproteins (HDL)**	___ ___ ___ mg/dl
10. **Low-density lipoproteins (LDL)**	___ ___ ___ mg/dl
11. **Triglycerides**	___ ___ ___ mg/dl

12. **Hemoglobin A1c (HA1c)**	___ ___ . ___ %

The following should be obtained from both the Medical Record and patient:

**Screening or Test**	**Response from Medical Record**		**Response from Patient**	
	**Date of Last Screening** *MM/YYYY*	**Not Found**	**Date of Last Screening** *MM/YYYY*	**No/Not Performed**
13. **Most recent mammogram – Women age 40-69 only**	___ ___/___ ___ ___ ___	❑	___ ___/___ ___ ___ ___	❑
14. **Most recent cervical cancer screening (Pap test) – Women age 21-63 only**	___ ___/___ ___ ___ ___	❑	___ ___/___ ___ ___ ___	❑
**Most recent colorectal cancer screening – Age 50-75 only**				
15. **Colonoscopy (flexible fiberoptic/optical)**	___ ___/___ ___ ___ ___	❑	___ ___/___ ___ ___ ___	❑
16. **3 Card FOBT (guaiac)**	___ ___/___ ___ ___ ___	❑	___ ___/___ ___ ___ ___	❑
17. **3 Card Fecal Immunochemical Test (FIT)**	___ ___/___ ___ ___ ___	❑	___ ___/___ ___ ___ ___	❑
18. **2 Card Fecal Immunochemical Test (FIT)**	___ ___/___ ___ ___ ___	❑	___ ___/___ ___ ___ ___	❑
19. **Flexible Sigmoidoscopy**	___ ___/___ ___ ___ ___	❑	___ ___/___ ___ ___ ___	❑
20. **CT colonoscopy/CT colonography**	___ ___/___ ___ ___ ___	❑	___ ___/___ ___ ___ ___	❑
21. **Digital rectal exam in office (guaiac)**	___ ___/___ ___ ___ ___	❑	___ ___/___ ___ ___ ___	❑

2014-09-23

Study ID: __ __ - __ __

Four and Eight Month Data Collection

(Intervention Sites Only)

**Time Since Enrollment** *(check one)*:	☐ **4 months**	☐ **8 months**

For every "Diagnosis" row listed below, complete only ***ONE*** of the following options:

- If the diagnosis is confirmed for the patient, either:
    a. Provide the date the test was performed and the value obtained ***IF*** it was completed since the review of laboratory tests at baseline enrollment or 4 months **OR**
    b. Check N/F if no new tests have been performed since baseline
- If the patient does NOT have the diagnosis, check No

Diagnosis	Has Diagnosis	Test	Test Date	Value	Not Found
Diabetes	☐ Yes ☐ No	HgA1c	__ __ / __ __ / __ __	__ __ . __ %	☐ N/F
Hypertension	☐ Yes ☐ No	Blood Pressure	__ __ / __ __ / __ __	______ / ______ mmHg	☐ N/F
Atrial Fibrillation	☐ Yes ☐ No	INR (if on Warfarin)	__ __ / __ __ / __ __	__ . __	☐ N/F
Hyperlipidemia	☐ Yes ☐ No	Total Cholesterol	__ __ / __ __ / __ __	__ __ __ mg/dl	☐ N/F
		LDL	__ __ / __ __ / __ __	__ __ __ mg/dl	☐ N/F
		HDL	__ __ / __ __ / __ __	__ __ __ mg/dl	☐ N/F
		Triglycerides	__ __ / __ __ / __ __	__ __ __ mg/dl	☐ N/F

I CARE
Improved Cardiovascular Risk Reduction to Enhance Rural Primary Care

Clinic Visit Tracking Form

Study ID: __ __- __ __

Study Time Point: ○ 12 month ○ 30 month

Baseline Study Visit: _ _ / _ _ / _ _ _ _ (mm/dd/yyyy)

Data Collection Date: _ _ / _ _ / _ _ _ _ (mm/dd/yyyy)

<u>Instructions</u>:

Review the patient's medical record. Complete a row for every patient visit to the clinic at which the patient saw a physician, nurse practitioner, physician's assistant, pharmacist or health coach.

- At the 12 month visit collect <u>ONLY</u> visits including baseline, 12 month and all visits in between.
- At month 30 collect <u>ONLY</u> visits occurring AFTER the 12 month visit and through 30 months.

Date of Clinic Visit	Chronic Conditions Addressed at This Visit (check all that apply)	Was this an annual physical?
_ _ / _ _ / _ _ _ _<br>Provider Type(s)*:<br>___ ___ ___	☐ Diabetes ☐ Coronary Artery Disease<br>☐ Cholesterol ☐ Peripheral Vascular Disease<br>☐ Hypertension ☐ Carotid Artery Disease<br>☐ COPD ☐ None of the above<br>☐ Atrial fibrillation	☐ Yes<br>☐ No
_ _ / _ _ / _ _ _ _<br>Provider Type(s)*:<br>___ ___ ___	☐ Diabetes ☐ Coronary Artery Disease<br>☐ Cholesterol ☐ Peripheral Vascular Disease<br>☐ Hypertension ☐ Carotid Artery Disease<br>☐ COPD ☐ None of the above<br>☐ Atrial fibrillation	☐ Yes<br>☐ No
_ _ / _ _ / _ _ _ _<br>Provider Type(s)*:<br>___ ___ ___	☐ Diabetes ☐ Coronary Artery Disease<br>☐ Cholesterol ☐ Peripheral Vascular Disease<br>☐ Hypertension ☐ Carotid Artery Disease<br>☐ COPD ☐ None of the above<br>☐ Atrial fibrillation	☐ Yes<br>☐ No
_ _ / _ _ / _ _ _ _<br>Provider Type(s)*:<br>___ ___ ___	☐ Diabetes ☐ Coronary Artery Disease<br>☐ Cholesterol ☐ Peripheral Vascular Disease<br>☐ Hypertension ☐ Carotid Artery Disease<br>☐ COPD ☐ None of the above<br>☐ Atrial fibrillation	☐ Yes<br>☐ No

* 1=MD, DO, PA, ARNP; 2= clinic pharmacist; 3=health coach *(fill in all that apply)*

Study ID: __ __- __ __

Date of Clinic Visit	Chronic Conditions Addressed at This Visit (check all that apply)	Was this an annual physical?
_ _ / _ _ / _ _ _ _<br>Provider Type(s)*:<br>___ ___ ___	☐ Diabetes ☐ Coronary Artery Disease<br>☐ Cholesterol ☐ Peripheral Vascular Disease<br>☐ Hypertension ☐ Carotid Artery Disease<br>☐ COPD ☐ None of the above<br>☐ Atrial fibrillation	☐ Yes<br>☐ No
_ _ / _ _ / _ _ _ _<br>Provider Type(s)*:<br>___ ___ ___	☐ Diabetes ☐ Coronary Artery Disease<br>☐ Cholesterol ☐ Peripheral Vascular Disease<br>☐ Hypertension ☐ Carotid Artery Disease<br>☐ COPD ☐ None of the above<br>☐ Atrial fibrillation	☐ Yes<br>☐ No
_ _ / _ _ / _ _ _ _<br>Provider Type(s)*:<br>___ ___ ___	☐ Diabetes ☐ Coronary Artery Disease<br>☐ Cholesterol ☐ Peripheral Vascular Disease<br>☐ Hypertension ☐ Carotid Artery Disease<br>☐ COPD ☐ None of the above<br>☐ Atrial fibrillation	☐ Yes<br>☐ No
_ _ / _ _ / _ _ _ _<br>Provider Type(s)*:<br>___ ___ ___	☐ Diabetes ☐ Coronary Artery Disease<br>☐ Cholesterol ☐ Peripheral Vascular Disease<br>☐ Hypertension ☐ Carotid Artery Disease<br>☐ COPD ☐ None of the above<br>☐ Atrial fibrillation	☐ Yes<br>☐ No
_ _ / _ _ / _ _ _ _<br>Provider Type(s)*:<br>___ ___ ___	☐ Diabetes ☐ Coronary Artery Disease<br>☐ Cholesterol ☐ Peripheral Vascular Disease<br>☐ Hypertension ☐ Carotid Artery Disease<br>☐ COPD ☐ None of the above<br>☐ Atrial fibrillation	☐ Yes<br>☐ No
_ _ / _ _ / _ _ _ _<br>Provider Type(s)*:<br>___ ___ ___	☐ Diabetes ☐ Coronary Artery Disease<br>☐ Cholesterol ☐ Peripheral Vascular Disease<br>☐ Hypertension ☐ Carotid Artery Disease<br>☐ COPD ☐ None of the above<br>☐ Atrial fibrillation	☐ Yes<br>☐ No

* 1=MD, DO, PA, ARNP; 2= clinic pharmacist; 3=health coach *(fill in all that apply)*

ICARE
Improved Cardiovascular Risk Reduction to Enhance Rural Primary Care

30 Month Blood Pressure, Laboratory and Medication Form

This form should be completed and faxed no later than 37 months following enrollment

**Study ID:** _ _ _ _ _	**Today's Date:** *(mm/dd/yyyy)* _ _ / _ _ / _ _ _ _
**Enrollment date:**	_ _ / _ _ / _ _ _ _
**Date 24 months after enrollment:**	_ _ / _ _ / _ _ _ _
**Date 30 months after enrollment:**	_ _ / _ _ / _ _ _ _

A. Record values documented in the patient's medical record no earlier than 24 months after enrollment and no later than 30 months following enrollment:

- If multiple values are found, select the value that is closest to 30 months following enrollment
- If there are no values found between 24-30 months after enrollment, mark "no entry in time frame"

	Date (mm/dd/yyy)	a. Systolic BP (mm Hg)	b. Diastolic BP (mm Hg)	No Entry in Time Frame
1. **Blood Pressure**	_ _ / _ _ / _ _ _ _	___ ___ ___	___ ___ ___	☐
2. **Total Cholesterol**	_ _ / _ _ / _ _ _ _	___ ___ ___ mg/dl		☐
3. **High-density lipoproteins (HDL)**	_ _ / _ _ / _ _ _ _	___ ___ ___ mg/dl		☐
4. **Low-density lipoproteins (LDL)**	_ _ / _ _ / _ _ _ _	___ ___ ___ mg/dl		☐
5. **Triglycerides**	_ _ / _ _ / _ _ _ _	___ ___ ___ mg/dl		☐
6. **Hemoglobin A1c (HA1c)**	_ _ / _ _ / _ _ _ _	___ ___ . ___ %		☐

Study ID: _ _-_ _

B. Record medications documented in the patient's medical record no earlier than 24 months after enrollment and no later than 30 months following enrollment:

- **If multiple lists are found, select the list that is closest to 30 months following enrollment.**
- ONLY include **antihypertensive agents**, **hyperglycemic agents**, **cholesterol agents**, **asthma agents** and **anticoagulants/antiplatelet agents**

Medication Name & Code	Strength	Directions for Use
**1.** ___________________________<br>**Code:** __ __ __ __		
**2.** ___________________________<br>**Code:** __ __ __ __		
**3.** ___________________________<br>**Code:** __ __ __ __		
**4.** ___________________________<br>**Code:** __ __ __ __		
**5.** ___________________________<br>**Code:** __ __ __ __		
**6.** ___________________________<br>**Code:** __ __ __ __		
**7.** ___________________________<br>**Code:** __ __ __ __		
**8.** ___________________________<br>**Code:** __ __ __ __		

Medication Name & Code	Strength	Directions for Use
**9.** ___________________________<br>**Code:** __ __ __ __		
**10.** ___________________________<br>**Code:** __ __ __ __		
**11.** ___________________________<br>**Code:** __ __ __ __		
**12.** ___________________________<br>**Code:** __ __ __ __		
**13.** ___________________________<br>**Code:** __ __ __ __		
**14.** ___________________________<br>**Code:** __ __ __ __		
**15.** ___________________________<br>**Code:** __ __ __ __		
**16.** ___________________________<br>**Code:** __ __ __ __		

UNANTICIPATED PROBLEM (UP) SCREENING FORM
12-MONTH STUDY VISIT

DEFINITION

An **Unanticipated Problem (UP)** is any event or problem that is:

A. Unexpected, **AND**
B. Possibly, probably, or definitely related to study participation, **AND**
C. Suggests greater risk of harm to study participant(s) than was previously known or recognized, including a breach of confidentiality, a subject complaint that can't be resolved by study investigators, or identification of a new risk related to the study

INSTRUCTIONS

The purpose of this form is to help ensure that all UPs have been identified and reported for each study subject during the first 12 months of the subject's participation in the study.

After meeting with the subject for the 12-month study visit, please review the subject's medical record for the previous 12 months and answer the questions that follow.

The following questions guide you through each of the three criteria above for an Unanticipated Problem (UP). Please follow the prompts in the questions that follow and fax the completed form to Nick Rudzianski at the University of Iowa at 319-335-9782, and your local IRB if applicable.*

**PLEASE NOTE: If you are approved to conduct this study by a local Institutional Review Board (IRB) instead of the University of Iowa IRB, all site communication related to UPs reported to your local should also be forwarded to the University of Iowa research team.*

START HERE

1. 12-month study visit date: ____ ____/ ____ ____ / ____ ____ ____ ____ (MM/DD/YYYY)

2. After meeting with the subject and reviewing the subject's medical record, have you identified one or more **incidents, experiences, or outcomes that are unexpected in terms of nature, severity or frequency that occurred** between the time the subject enrolled in the study (signed consent) and the 12-month study visit date?
   ❒ NO ➔ ***Skip to Question 4***
   ❒ YES ➔ ***Continue to Question 3 below***

3. If YES, please describe the unexpected nature of each occurence:_________________
____
____
____

4. After meeting with the subject and reviewing the subject's medical record, have you identified one or more **incidents, experiences, or outcomes that are <u>related or possibly related to this subject's participation in the research study</u>** between the time the subject enrolled in the study (signed consent) and the 12-month study visit date?
   - ❒ NO ➔ ***Skip to Question 6***
   - ❒ YES➔ ***Continue to Question 5 below***

5. If YES, please describe how each occurence was related to the subject's participation in the research study:____

   ____

   ____

6. After meeting with the subject and reviewing the subject's medical record, have you identified one or more **incidents, experiences, or outcomes that are <u>which suggest that the research places subjects or others at a greater risk of harm (including physical, psychological, economic, or social harm) than was previously known or recognized</u>** between the time the subject enrolled in the study (signed consent) through the 12-month study visit date?
   - ❒ NO ➔ ***Skip to the box below ("!!! READ BEFORE CONTINUING !!!")***
   - ❒ YES➔ ***Continue to Question 7 below***

7. If YES, please describe how each occurence placed the subject or others at increased risk of harm: ____

   ____

   ____

!!! READ BEFORE CONTINUING !!!

⇨ If you answered "**YES**" **to Questions 2, 4, AND 6, a UP was identified – please continue with Question 8 on the next page.**

⇨ If you answered "**NO" to Question 2, 4 OR 6:**

- STOP here. Your responses indicate that no Unanticipated Problem involving this subject occurred between the baseline visit and the 12-month study visit.
- Do not complete the remaining section.
- Fax this form to Nick Rudzianski at 319-335-9782.
- Date faxed: __ __/ __ __ / __ __ __ __ (MM/DD/YYYY)
- File this hard copy form in the subject's study folder.

8. Have **ALL** of the Unanticipated Problems that you identified above in Questions 2-7 been previously reported to the University of Iowa? That is, have you completed and faxed "UNANTICIPATED PROBLEM (UP) EVENT-DRIVEN" forms for each of these?

YES	NO
❑	❑
↓	↓
You indicated that ALL occurrences of Unanticipated Problems involving this subject have been reported to the University of Iowa.<br><br>Please take the following actions:<br>• Fax this form to Nick Rudzianski at 319-335-9782.<br>• Date faxed: ___ ___/ ___ ___ / ___ ___ ___ ___ (MM/DD/YYYY)<br>• File this hard copy form in the subject's study folder.	You indicated that one or more Unanticipated Problems involving this subject **have not been reported** to the University of Iowa.<br><br>**It is important that you submit all Unantipcated Problems as soon as possible**.<br><br>Please take the following actions:<br>• Submit an "UNANTICIPATED PROBLEM (UP) EVENT-DRIVEN" form for each event you identified in Questions 2-7 above.<br>• Fax this form to Nick Rudzianski at 319-335-9782.<br><br>If you have any questions about Unanticipated Problems or the process of reporting them to the University of Iowa, please contact Brian Gryzlak at 319-335-8218 or email brian-gryzlak@uiowa.edu

UNANTICIPATED PROBLEM (UP) EVENT-DRIVEN FORM

DEFINITION

An **Unanticipated Problem (UP)** is any event or problem that is:

A. Unexpected, **AND**
B. Possibly, probably, or definitely related to study participation, **AND**
C. Suggests greater risk of harm to study participant(s) than was previously known or recognized, including a breach of confidentiality, a subject complaint that can't be resolved by study investigators, or identification of a new risk related to the study.

INSTRUCTIONS
The purpose of this form is to document and report Unanticipated Problems that have been identified.

The following questions guide you through each of the three criteria above for an Unanticipated Problem (UP). Unless prompted otherwise below, please complete and fax this form Nick Rudzianski at the University of Iowa at 319-335-9782, and your local IRB if applicable.*

PLEASE NOTE: If you are approved to conduct this study by a local Institutional Review Board (IRB) instead of the University of Iowa IRB, all site communication related to UPs reported to your local should also be forwarded to the University of Iowa research team.

START HERE

1. How many subjects did the Unanticipated Problem (UP) you are reporting affect or impact?

- ❒ The UP affected **more than one subject** ➔ ***Skip to Question 3***
- ❒ The UP affected **only one subject** ➔ ***Continue to Question 2 below***

2. Enter the study ID for the affected subject: ____ ____ - ____ ____

3. Have you identified an **incident, experience, or outcome that is unexpected in terms of nature, severity or frequency for this subject(s)?**

- ❒ NO ➔ ***Skip to Question 5***
- ❒ YES ➔ ***Continue to Question 4 below***

4. If YES, please describe how the event was unexpected: ________________________
____
____

5. Have you identified an **incident, experience, or outcome that is <u>related or possibly related to this subject's participation in the research study</u>?**

❒ NO ➔ ***Skip to Question 7***
❒ YES➔ ***Continue to Question 6 below***

6. If YES, please describe how the event was related to the subject's participation in the study: ____

____

____

7. Have you identified an **incident, experience, or outcome that <u>suggests that the research places subjects or others at a greater risk of harm (including physical, psychological, economic, or social harm) than was previously known or recognized</u>?**

❒ NO ➔ ***Skip to the box below ("!!! READ BEFORE CONTINUING !!!")***
❒ YES➔ ***Continue to Question 8 below***

8. If YES, please describe how the event places subjects or others at greater risk of harm:

____

____

!!! READ BEFORE CONTINUING !!!

⇨ If you answered "**YES" to Questions 3, 5, AND 7**, an Unanticipated Problem (UP) was identified – please continue with Question 9 below. <u>After you complete this form, please fax it to Nick Rudzianski at 319-335-9782 and file this hard copy form in the appropriate study folder.</u>

⇨ If you answered **"NO" to Question 3, 5 OR 7:**

- STOP HERE. Your responses indicate that the event you are trying to report is not an Unanticipated Problem.
- Do not complete the remaining sections.
- File this hard copy form in the appropriate study folder.

⇨ If you think that the event you are reporting should still be considered an Unanticipated Problem, please contact the ICARE study coordinator, Brian Gryzlak at 319-335-8218 or brian-gryzlak@uiowa.edu

9. Briefly describe the UP, including any harm or potential harm that occurred to subject(s), whether and how the incident was resolved. (If you have supplementary information related to this UP that you think needs to be reviewed by the study team please fax it to Nick Rudzianski at the University of Iowa at 319-335-9782.)

____

____

10. On what date did this UP occur?
__ __/__ __/__ __ __ __ (MM/DD/YYYY)

11. On what date was this UP identified by your clinic?
__ __/__ __/__ __ __ __ (MM/DD/YYYY)

For each of the questions below, please indicate whether you feel the outcome described is attributed to the UP you are reporting. Answers to Questions 11-19 are required.

12. Did any subject **die** as a result of the UP?

- ❒ NO ➔ ***Skip to Question 13***
- ❒ YES ➔ ***Continue to Question 12 below***

13. Death date: __ __/__ __/__ __ __ __ (MM/DD/YYYY)

14. Did any subject experience a **life-threatening condition** as a result of the UP?

- ❒ YES
- ❒ NO

15. Was any subject **hospitalized** as a result of the UP?

- ❒ YES
- ❒ NO

16. Did any subject experience **disability** as a result of the UP?

- ❒ YES
- ❒ NO

17. Did the UP result in **congenital abnormality**?

- ❒ YES
- ❒ NO

18. Did the UP **require intervention in order to prevent permanent impairment or damage** to any subject?

- ❒ YES
- ❒ NO

NOTE: A "yes" answer to Question 17 should only be used for an event that does not result in death, a life-threatening condition, hospitalization, disability or congenital deformity, but that did jeopardize the subject(s) and that required a specific medical intervention to prevent one or more of outcomes in Questions 12-17 from occurring.

19. Did the UP **result in an important medical event** as determined by a physician or designee at your clinic?

❒ YES
❒ NO

NOTE: A "yes" answer to Question 19 should only be used when a site judges the event to represent significant hazard or harm to a research subject(s).

20. Was the UP an **exacerbation of a pre-existing condition** (that is, it existed prior to the subject's enrollment in the study)?

❒ YES
❒ NO

21. Describe any details about the UP that might help us evaluate its relationship to the study.

____

____

____

22. Describe other relevant history, including pre-existing medical conditions (e.g. allergies, race, pregnancy, smoking and alcohol use, hepatic/renal dysfunction).

____

____

____

23. Describe relevant scans/tests/laboratory data related to the UP, including dates.

____

____

____

24. Describe any action that you have taken as a result of the problem, whether to moderate the impact on the subject(s) or to decease the likelihood that the problem will recur:

____

____

____

Please fax this form to Nick Rudzianski at 319-335-9782 after it is completed and file this hard copy in the appropriate study folder.

Study Termination

(Complete after the 30 month BP-Labs-Medication Form is faxed
OR when the patient is terminated early)

1. Did the subject complete all study time points:
   - ☐ Yes
     - a. Date of 12 month visit: _ _/_ _/_ _ _ _
     - b. Date of 30 month data submission: _ _/_ _/_ _ _ _
   - ☐ No
     - b. Date of early termination: _ _/_ _/_ _ _ _
2. If the subject terminated the study early, please indicate the reason:
   - ☐ Subject eligibility status changed
     - a. Reason: ___________________________________
   - ☐ Subject chose to withdraw
     - a. Reason: ___________________________________
   - ☐ Subject lost to follow-up (unable to contact)
   - ☐ Subject is no longer a patient at this clinic
   - ☐ Research team chose to discontinue subject
     - a. Reason: ___________________________________
   - ☐ Subject withdrew/terminated due to an adverse event
     - a. Reason: ___________________________________
   - ☐ Subject death (enter death date for question 1.b)
   - ☐ Other
     - a. Specify: ___________________________________
3. Comments:

____

____

APPENDIX VI: PHARMACIST-PHYSICIAN COMMUNICATION FORM TEMPLATE

ICARE
Improved Cardiovascular Risk Reduction to Enhance Rural Primary Care

Communication Form

Primary Provider:	Dr. Who	Fax:	(111) - 222 - 3333
Patient Name:	Harold Maudelin	Date of Birth:	05/05/1988
Pharmacist:	Demo Pharmacist 01	Date:	11/10/2014
Return Fax:	(319) - 384 - 1728		

Communication Type
☒ Initial ☐ Follow-Up ☐ New Problem ☐ Preventive ☐ Other

This is the message.

This is recommendation #1.

This is recommendation #2.

Thank you for your time,
Demo Pharmacist 01
ICARE CVRS Pharmacist

Recommended Pharmacist Follow-up Assessment - 2 Weeks

☐ I agree with the above recommendations.

☐ Proposed modified plan:

Primary Provider Signature: ______________________________
Date: ____________

This form will serve as a prescription if recommendations are accepted by the physician.
Information on this fax is confidential. If you have received this fax in error please destroy and call 866-227-9873.

APPENDIX VII: PATIENT BROCHURE

Additional information about this study

- Participation is completely voluntary and you may choose to stop at any time.
- We encourage you to talk about your participation with your family and friends.
- You will receive a free online Personal Health Record account as part of this study.
- This study is funded by the National Heart, Lung, and Blood Institute (NHLBI) and carried out by the University of Iowa.
- You will **not** be asked to travel to the University of Iowa for any part of this study.
- Twelve physician offices across Iowa, including [site name], have agreed to participate in this study as a research site.
- We have randomly selected half of these sites to have a University of Iowa pharmacist join their care team to monitor and communicate with their patients.
- Study site locations:

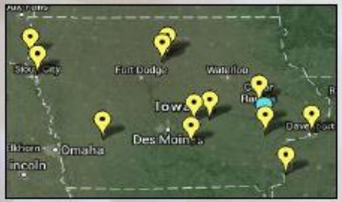

If you are interested in learning more about this study, or to see if you qualify, please contact:

[site , staff , and contact info]

Improved Cardiovascular Risk Reduction to Enhance Primary Care ("ICARE") Study

Information for Patients

[site name]

In collaboration with the University of Iowa

ICARE
Improved Cardiovascular Risk Reduction to Enhance Rural Primary Care

What is this study about?

This study is designed to test whether adding pharmacists to doctor office care teams can help improve the cardiovascular (heart) health of patients with certain risk factors. [site name] was randomly selected to {"**not**" if control site} have a pharmacist added to their team. This means that you would {"**not**" if control site} communicate with a study pharmacist during the study if you decide to join.

Am I eligible to participate?

You **may be eligible** to participate if you meet the following criteria:

- ✓ Are age 50 or older
- ✓ Can communicate in English over the phone
- ✓ AND have **three or more** of the following conditions:
  - Diabetes
  - High cholesterol
  - High blood pressure
  - Coronary artery disease
  - Previous heart attack
  - Previous stroke/TIA
  - Atrial fibrillation
  - Peripheral vascular disease
  - Carotid artery disease
  - Current smoker
  - Obesity

You are **not eligible** to participate if any of the following are true for you:

- Have pulmonary hypertension
- Are pregnant
- Have certain kinds of cancer
- Live in a nursing home or have dementia
- Cannot have your blood pressure taken using an arm cuff

The study coordinator in your clinic (see back of brochure) can tell you whether you are eligible for participation.

What will I be asked to do?

- You would be asked to come in to your doctor's office for two study visits, about 12 months apart.
- During these visits, you would have your blood pressure taken, get a blood test, and answer questions about your health.
- You would also be asked to grant the research team permission to record certain information about conditions and medications from your medical record.
- {if control site, omit this line] You would be contacted by a study pharmacist during the first 12 months of the study .
- You would be paid $75.00 for each of the two study visits you complete for a total of $150.

APPENDIX VIII: STUDY DRUG CODES

Code	Generic Name	Brand Names	Strengths Available
2101	acarbose	Precose	25 mg, 50 mg, 100 mg
201	acebutolol	Sectral	200 mg, 400 mg
1101	aliskiren	Tekturna	150 mg, 300 mg
1102	aliskiren-hydrochlorothiazide	Tekturna HCT	150/12.5 mg, 150/25 mg, 300/12.5 mg, 300/25 mg
1103	aliskiren-valsartan	Valturna	150/160 mg, 300/320 mg
101	amiloride	Midamor	5 mg
102	amiloride/hydrochlorothiazide*	Moduretic	5/50 mg
401	amlodipine	Norvasc	2.5 mg, 5 mg, 10 mg
410	amlodipine/benazepril*	Lotrel	2.5/10 mg, 5/10 mg, 5/20 mg, 5/40 mg, 10/20 mg, 10/40 mg
422	amlodipine/olmesartan	AZOR	5/20 mg, 5/40 mg, 10/20 mg, 10/40 mg
420	amlodipine/valsartan*	Exforge	5/160 mg, 10/160 mg, 5/320 mg, 10/320 mg
421	amlodipine / valsartan / hydrochlorothiazide	Exforge HCT	5/160/12.5 mg, 10/160/12.5 mg, 5/160/25 mg, 10/160/25 mg, 10/320/25 mg
5202	apixaban	Eliquis	2.5mg, 5mg
6201	arformoterol tartrate	Brovana	15 mcg
2001	aspart	Novolog	100U/ml
2002	aspart protamine	Novolog Mix	70/30 – 100U/ml
5401	aspirin	Ecotrin, Bufferin, Aspergum	81mg, 227mg, 325mg, 500mg, 650mg
202	atenolol	Tenormin	25 mg, 50 mg, 100 mg
213	atenolol-chlorthalidone	Tenoretic	50/25 mg, 100/25 mg
4001	atorvastatin	Lipitor	10mg, 20mg, 40mg, 80mg
4301	atorvastatin/amlodipine*	Caduet	2.5/10mg, 2.5/20mg, 2.5/40mg, 5/10mg, 5/20mg, 5/40mg, 5/80mg, 10/10mg, 10/20mg, 10/40mg, 10/80mg
615	azilsartan medoxomil	Edarbi	40 mg, 80 mg
6101	beclomethasone	Beclovent, QVAR	42 mcg, 40 mcg, 80 mcg
301	benazepril	Lotensin	5 mg, 10 mg, 20 mg, 40 mg
311	benazepril-hydrochlorothiazide*	Lotensin HCT	5/6.25 mg, 10/12.5 mg, 20/12.5 mg, 20/25 mg
203	betaxolol	Kerlone	10 mg, 20 mg
204	bisoprolol	Zebeta	5 mg, 10 mg
214	bisoprolol-hydrochlorothiazide*	Ziac	2.5/6.25 mg, 5/6.25 mg, 10/6.25 mg
6102	budesonide	Pulmicort Flexhaler	90 mcg, 180 mcg

Code	Generic Name	Brand Names	Strengths Available
6504	budesonide/ formoterol*	Symbicort	80 mcg-4.5 mcg, 160 mcg-4.5 mcg
103	bumetanide	Bumex	0.5 mg, 1 mg, 2 mg
3401	canagliflozin	Invokana	100mg, 300mg
601	candesartan	Atacand	4 mg, 8 mg, 16 mg, 32 mg
608	candesartan-hydrochlorothiazide	Atacand HCT	16/12.5 mg, 32/12.5 mg, 32/25 mg
302	captopril	Capoten	12.5 mg, 25 mg, 50 mg, 100 mg
312	captopril-hydrochlorothiazide*	Capozide	25/15 mg, 25/25 mg, 50/15 mg, 50/25 mg
220	carvedilol	Coreg	3.125 mg, 6.25 mg, 12.5 mg, 25 mg
222	carvedilol extended release	Coreg CR	10 mg, 20 mg, 40 mg, 80 mg
104	chlorothiazide	Diuril	250 mg, 500 mg
3101	chlorpropamide	Diabinese	100mg, 250mg
105	chlorthalidone	Hygroton and others	25 mg, 50 mg, 100 mg
4802	cholesteramine light	Questran Light, Prevalite	4 gr
4801	cholestyramine	Questran	4 gr
6103	ciclesonide	Alvesco	80 mcg, 160 mcg
701	clonidine	Catapres	0.1 mg, 0.2 mg, 0.3 mg
702	clonidine topical patch	Catapres TTS	0.1 mg, 0.2 mg, 0.3 mg
707	clonidine-chlorthalidone	Clorpres	0.1/15 mg, 0.2/15 mg, 0.3/15 mg
5101	clopidogrel	Plavix	75mg, 300mg
4804	colesevelam	WelChol	625mg tab, 3.75g powder
4803	colestipol	Colestid	1 gr tab, 5 gr granules
5301	dabigatran	Pradaxa	75mg, 150mg
5003	daltaperin	Fragmin	2500U, 5000U, 7500U, 10,000U, 12,500U, 15,000U, 18,000U, 25,000U
3402	dapagliflozin	Farxiga	5mg, 10mg
2003	detemir	Levemir	100U/ml
402	diltiazem	Cardizem, Dilacor, Tiazac	30 mg, 60 mg, 90 mg, 120 mg, 180 mg, 240 mg, 300 mg, 360 mg, 420 mg
501	doxazosin	Cardura	1 mg, 2 mg, 4 mg, 8 mg
303	enalapril	Vasotec	2.5 mg, 5 mg, 10 mg, 20 mg
411	enalapril/felodipine *	Lexxel	5/2.5 mg, 5/5 mg

Code	Generic Name	Brand Names	Strengths Available
313	enalapril-hydrochlorothiazide*	Vaseretic	5/12.5 mg, 10/25 mg
5002	enoxaparin	Lovenox	30mg, 40mg, 60mg, 80mg, 100mg, 120mg, 150mg, 300mg
1001	eplerenone	Inspra	25 mg, 50 mg
602	eprosartan	Teveten	400 mg, 600 mg
609	eprosartan-hydrochlorothiazide	Teveten-HCT	600/12.5 mg, 600/25 mg
115	ethacrynic acid	Edecrin	25g
3001	exanatide	Byetta	5mcg, 10mcg
4601	exetimibe	Zetia	10mg
403	felodipine	Plendil	2.5 mg, 5 mg, 10 mg
4902	fenofibrate	Lipofen, Lofibra, Tricor, Triglide	40mg, 48mg, 50mg, 54mg, 120mg, 145mg, 160mg
4903	fenofibrate - micronized	Lofibra, Antara	43mg, 67mg, 130mg, 134mg, 200mg
6104	flunisolide	Aerobid	.25 mg
6105	fluticasone propionate	Flovent Diskus, Flovent, Flovent HFA	50 mcg, 100 mcg, 250 mcg, 44 mcg, 110 mcg, 220 mcg
6501	fluticasone/ salmeterol*	Advair Diskus, Advair HFA	100mcg-50 mcg, 250 mcg-50 mcg, 500 mcg-50 mcg, 45 mcg-21 mcg, 115 mcg-21 mcg, 230 mcg-21 mcg
6502	fluticasone/ vilanterol*	BREO ELLIPTA	100 mcg-25 mcg
4003	fluvastatin, fluvastatin XL	Lescol, Lescol XL	20mg, 40mg, 80mg
6202	formoterol fumarate	Foradil Aerolizer Inhaler	12 mcg
304	fosinopril	Monopril	10 mg, 20 mg, 40 mg
314	fosinopril-hydrochlorothiazide*	Monopril-HCT	10/12.5 mg, 20/12.5 mg
106	furosemide	Lasix	20 mg, 40 mg, 80 mg
4901	gemfibrozil	Lopid	600mg
2004	glargine	Lantus	100U/ml
3102	glimeperide	Amaryl	1mg, 2mg, 4mg
3301	glimepiride/pioglitazone*	Duetact	2/30mg, 4/30mg
3302	glimepiride/rosiglitazone*	Avandaryl	1/4mg, 2/4mg, 4/4mg,2/8mg, 4/8mg
3103	glipizide	Glucotrol	5mg, 10mg
3104	glipizide XR	Glucotrol XR	2.5mg, 5mg, 10mg
3201	glipizide/metformin*	Metaglip	2.5/250mg, 2.5/500mg, 5/500mg
2005	glulisine	Apidra	100U/ml

Code	Generic Name	Brand Names	Strengths Available
3105	glyburide	Diabeta, Micronase	1.25mg, 2.5mg, 5mg
3106	glyburide – micronized	Glynase	1.5mg, 3mg, 6mg
3202	glyburide/metformin*	Glucovance	1.25/250mg, 2.5/500mg, 5/500mg
703	guanabenz	Wytensin	4 mg, 8 mg
704	guanfacine	Tenex	1 mg, 2 mg
901	hydralazine	Apresoline	10 mg, 25 mg, 50 mg, 100 mg
905	hydralazine-hydrochlorothiazide	Apresazide and Hydra-Zide	25/25 mg, 50/50 mg, 100/50 mg
107	hydrochlorothiazide	Hydrodiuril & others	12.5 mg, 25 mg, 50 mg
6203	indacaterol maleate	Arcapta Neohaler	75 mcg
109	indapamide	Lozol	1.25 mg, 2.5 mg
603	irbesartan	Avapro	75 mg, 150 mg, 300 mg
610	irbesartan-hydrochlorothiazide	Avalide	150/12.5 mg, 300/12.5 mg, 300/25 mg
2008	isophane	Humulin N, Novolin N	100U/ml
902	isosorbide dinitrate	Isordil	2.5 mg, 5 mg, 10 mg, 20 mg, 30 mg, 40 mg
906	isosorbide dinitrate-hydralazine	BiDil	20/37.5 mg
903	isosorbide mononitrate	Imdur	10 mg, 20 mg, 30 mg, 60 mg, 120 mg
404	isradipine	DynaCirc	2.5 mg, 5 mg, 10 mg
221	labetalol	Normodyne, Trandate	100 mg, 200 mg, 300 mg
2404	linagliptin	Tradjenta	5mg
2503	linagliptin-metformin*	Jentadueto	2.5/500mg, 2.5/850mg, 2.5/1000mg
3002	liraglutide	Victoza	18mg/3ml
305	lisinopril	Zestril, Prinivil	2.5 mg, 5 mg, 10 mg, 20 mg, 30 mg, 40 mg
315	lisinopril-hydrochlorothiazide*	Prinzide, Zestoretic	10/12.5 mg, 20/12.5 mg, 20/25 mg
2006	lispro	Humalog	100U/ml
2007	lispro protamine	Humalog Mix	50/50, 75/25
604	losartan	Cozaar	25 mg, 50 mg, 100 mg
611	losartan-hydrochlorothiazide	Hyzaar	50/12.5 mg, 100/12.5 mg, 100/25 mg
4004	lovastatin, lovastatin XR	Mevacor, Altoprev	10mg, 20mg, 40mg, 60mg
4101	lovastatin/niacin*	Advicor	500/20mg, 750/20mg, 1000/20mg, 1000/40mg
2301	metformin	Glucophage, Fortamet, Appformin,	500mg, 750mg, 850mg, 1000mg

Code	Generic Name	Brand Names	Strengths Available
		Glumetza	
2701	metformin/repaglinide*	Prandimet	1/500mg, 2/500mg
705	methyldopa	Aldomet	250 mg, 500 mg
706	methyldopa-hydrochlorothiazide	Aldoril	250/15 mg, 250/25 mg
110	metolazone	Mykrox, Zaroxolyn	2.5 mg, 5 mg, 10 mg
206	metoprolol succinate (extended release)	Toprol XL	25 mg, 50 mg, 100 mg, 200 mg
205	metoprolol tartrate	Lopressor	25 mg, 50 mg, 100 mg
215	metoprolol-hydrochlorothiazide*	Lopressor HCT	50/25 mg, 100/25 mg, 100/50 mg
2102	miglitol	Glyset	25mg, 50mg, 100mg
904	minoxidil	Loniten	2.5 mg, 10 mg
306	moexipril	Univasc	7.5 mg, 15 mg
316	moexipril-hydrochlorothiazide*	Uniretic	7.5/12.5 mg, 15/12.5 mg, 15/25 mg
6106	mometasone	Asmanex Twisthaler/ Twist	220 mcg, 110 mcg
6503	mometasone/ formoterol*	Dulera	100 mcg-5 mcg, 200 mcg-5 mcg
6301	montelukast	Singulair	4 mg, 5 mg, 10 mg
207	nadolol	Corgard	20 mg, 40 mg, 80 mg, 120 mg, 160 mg
216	nadolol-bendroflumethiazide*	Corzide	40/5 mg, 80/5 mg
2601	nateglinide	Starlix	60mg, 120mg
219	nebivolol	Bystolic	2.5 mg, 5 mg, 10 mg, 20 mg
4501	niacin**	Niaspan	500mg, 750mg, 1000mg
405	nicardipine	Cardene	20 mg, 30 mg, 45 mg, 60 mg
406	nifedipine	Adalat, Procardia	10 mg, 20 mg, 30 mg, 60 mg, 90 mg
407	nisoldipine	Sular	8.5 mg, 10 mg, 17 mg, 20 mg, 25.5 mg, 30 mg, 34 mg, 40 mg
605	olmesartan	Benicar	5 mg, 20 mg, 40 mg
612	olmesartan medoxomil-hydrochlorothiazide	Benicar HCT	20/12.5 mg, 40/12.5 mg, 40/25 mg
4701	omega – 3 fatty acids**	Lovaza	1 gr
208	penbutolol	Levatol	20 mg
307	perindopril	Aceon	2 mg, 4 mg, 8 mg
209	pindolol	Visken	5 mg, 10 mg
2801	pioglitazone	Actos	15mg, 30mg, 45mg

Code	Generic Name	Brand Names	Strengths Available
2901	pioglitazone/metformin*	ActoPlus	15/500mg, 15/850mg, 15/1000mg, 30/1000mg
4007	pitavastatin	Livalo	1mg, 2mg, 4mg
111	polythiazide	Renese	1 mg, 2 mg, 5 mg
2201	pramlintide	Symlin	60 and 120
5103	prasugrel	Effient	5mg, 10mg
4005	pravastatin	Pravachol	10mg, 20mg, 40mg, 80mg
502	prazosin	Minipress	1 mg, 2 mg, 5 mg
504	prazosin/polythiazide*	Minizide	1/0.5 mg, 2/0.5 mg, 5/0.5 mg
210	propranolol	Inderal	10 mg, 20 mg, 40 mg, 60 mg, 80 mg
217	propranolol la-hydrochlorothiazide*	Inderide LA	40/25 mg, 80/25 mg
211	propranolol long-acting	Inderal LA	60 mg, 80 mg, 120 mg, 160 mg
308	quinapril	Accupril	5 mg, 10 mg, 20 mg, 40 mg
317	quinapril-hydrochlorothiazide*	Accuretic	10/12.5 mg, 20/12.5 mg, 20/25 mg
309	ramipril	Altace	1.25 mg, 2.5 mg, 5 mg, 10 mg
2009	regular	Humulin R, Novolin R	100U/ml, 500U/ml
2010	regular:isophane	NPH	Novolin 70/30, Humuilin 50/50, Humulin 70/30
2602	repaglinide	Prandin	0.5mg, 1mg, 2mg
801	reserpine	Serpalan, Serpasil	0.1 mg, 0.25 mg
805	reserpine/hydralazine/hydrochlorothiazide*	Ser Ap Es	0.1/25/15 mg
803	reserpine-chlorothiazide	Diupres	0.125/250 mg, 0.125/500 mg
802	reserpine-chlorthalidone	Demi-Regroton	0.125/25, 0.25/50
804	reserpine-hydrochlorothiazide	Hydropres	0.125/25 mg, 0.125/50 mg
5201	rivaroxaban	Xarelto	10mg, 15mg, 20mg
2802	rosiglitazone	Avandia	2mg, 4mg, 8mg
2902	rosiglitazone/metformin*	Avandamet	2/500mg, 2/1000mg, 4/500mg, 4/1000mg
4002	rosuvastatin	Crestor	5mg, 10mg, 20mg, 40mg
6204	salmeterol xinafoate	Serevent Diskus	50 mcg
2403	saxagliptin	Onglyza	2.5mg, 5mg
2504	sexagliptin/metformin XR*	Kombiglyze	2.5/1000mg, 5/500mg, 5/1000mg
4006	simvastatin	Zocor	10mg, 20mg, 40mg, 80mg

Code	Generic Name	Brand Names	Strengths Available
4201	simvastatin/exetimibe*	Vytorin	10/10mg, 10/20mg, 10/40mg, 10/80mg
4102	simvastatin/niacin*	Simcor	500/20mg, 500/40mg, 750/20mg, 1000/20mg, 1000/40mg
4401	simvastatin/sitagliptan*	Juvisync	10/100mg, 20/100mg, 40/100mg
2401	sitagliptin	Januvia	25mg, 50mg, 100mg
2502	sitagliptin/metformin XR*	Janumet XR	50/500mg, 50/1000mg, 100/1000mg
2501	sitagliptin/metformin*	Janumet	50/500mg, 50/1000mg, 100/1000mg
2402	sitagliptin/simvastatin (off-market)*	Juvasync	100-10mg, 100-20mg, 100-40mg
1002	spironolactone	Aldactone	25 mg, 50 mg, 100 mg
112	spironolactone/hydrochlorthiazide*	Aldactazide	25/25 mg, 50/50 mg
606	telmisartan	Micardis	20 mg, 40 mg, 80 mg
613	telmisartan-hydrochlorothiazide	Micardis-HCT	40/12.5 mg, 80/12.5 mg, 80/25 mg
503	terazosin	Hytrin	1 mg, 2 mg, 5 mg, 10 mg
6401	theophylline (12 hr)	Theo-Dur, TheoCap	100 mg, 200 mg, 300 mg, 450 mg
6402	theophylline (24 hr)	Theo-Time, Theo-24, Uniphyl, Theochron, Quibron-T	100 mg, 200 mg, 300 mg, 400 mg, 600 mg
5102	ticagrelor	Brilinta	90mg
5104	ticlopidine	Ticlid	250mg
212	timolol	Blocadren	5 mg, 10 mg, 20 mg
218	timolol-hydrochlorothiazide*	Timolide	10/25 mg
3107	tolazamide	Tolinase	250mg, 500mg
3108	tolbutaminde	Orinase	500mg
113	torsemide	Demadex	5 mg, 10 mg, 20 mg, 100 mg
310	trandolapril	Mavik	1 mg, 2 mg, 4 mg
412	trandolapril/verapamil*	Tarka	1/240 mg, 2/180 mg, 2/240 mg, 4/240 mg
114	triamterene	Dyrenium	50 mg, 100 mg
108	triamterene/hydrochlorothiazide*	Dyazide, Maxide	37.5/25 mg, 50/25 mg, 75/50 mg
607	valsartan	Diovan	40 mg, 80 mg, 160 mg, 320 mg
614	valsartan-hydrochlorothiazide	Diovan-HCT	80/12.5 mg, 160/12.5 mg, 160/25 mg, 320/12.5 mg, 320/25 mg
408	verapamil	Calan, Isoptin, Verelan, Coer, Covera HS	40 mg, 80 mg, 100 mg 120 mg, 200 mg, 180 mg, 240 mg, 300 mg, 360 mg
5001	warfarin	Coumadin, Jantoven	1mg, 2mg, 2.5mg, 3mg, 4mg, 5mg, 6mg, 7.5mg, 10mg

Code	Generic Name	Brand Names	Strengths Available
6302	zafirlukast	Accolate	10 mg, 20 mg
6303	zileuton	Zyflor CR, Zyflo	600 mg, 1.2 g

Drug Codes for Antihypertensive Agents

Diuretics – *Class Code = 100*

Code	Generic Name	Brand Names	Strengths Available
101	amiloride	Midamor	5 mg
102	amiloride/hydrochlorothiazide*	Moduretic	5/50 mg
103	bumetanide	Bumex	0.5 mg, 1 mg, 2 mg
104	chlorothiazide	Diuril	250 mg, 500 mg
105	chlorthalidone	Hygroton and others	25 mg, 50 mg, 100 mg
106	furosemide	Lasix	20 mg, 40 mg, 80 mg
107	hydrochlorothiazide	Hydrodiuril & others	12.5 mg, 25 mg, 50 mg
108	triamterene/hydrochlorothiazide*	Dyazide, Maxide	37.5/25 mg, 50/25 mg, 75/50 mg
109	indapamide	Lozol	1.25 mg, 2.5 mg
110	metolazone	Mykrox, Zaroxolyn	2.5 mg, 5 mg, 10 mg
111	polythiazide	Renese	1 mg, 2 mg, 5 mg
112	spironolactone/hydrochlorthiazide*	Aldactazide	25/25 mg, 50/50 mg
113	torsemide	Demadex	5 mg, 10 mg, 20 mg, 100 mg
114	triamterene	Dyrenium	50 mg, 100 mg
115	Ethacrynic acid	Edecrin	25 g

* list specific strength of each ingredient

Beta Blockers – *Class Code = 200*

Code	Generic Name	Brand Names	Strengths Available
201	acebutolol	Sectral	200 mg, 400 mg
202	atenolol	Tenormin	25 mg, 50 mg, 100 mg
203	betaxolol	Kerlone	10 mg, 20 mg
204	bisoprolol	Zebeta	5 mg, 10 mg
205	metoprolol tartrate	Lopressor	25 mg, 50 mg, 100 mg
206	metoprolol succinate (extended release)	Toprol XL	25 mg, 50 mg, 100 mg, 200 mg
207	nadolol	Corgard	20 mg, 40 mg, 80 mg, 120 mg, 160 mg
208	penbutolol	Levatol	20 mg
209	pindolol	Visken	5 mg, 10 mg
210	propranolol	Inderal	10 mg, 20 mg, 40 mg, 60 mg, 80 mg
211	propranolol long-acting	Inderal LA	60 mg, 80 mg, 120 mg, 160 mg
212	timolol	Blocadren	5 mg, 10 mg, 20 mg
213	atenolol-chlorthalidone	Tenoretic	50/25 mg, 100/25 mg
214	bisoprolol-hydrochlorothiazide*	Ziac	2.5/6.25 mg, 5/6.25 mg, 10/6.25 mg
215	metoprolol-hydrochlorothiazide*	Lopressor HCT	50/25 mg, 100/25 mg, 100/50 mg

Beta Blockers – *Class Code = 200* (cont)

Code	Generic Name	Brand Names	Strengths Available
216	nadolol-bendroflumethiazide*	Corzide	40/5 mg, 80/5 mg
217	propranolol LA-hydrochlorothiazide*	Inderide LA	40/25 mg, 80/25 mg
218	timolol-hydrochlorothiazide*	Timolide	10/25 mg
219	Nebivolol	Bystolic	2.5 mg, 5 mg, 10 mg, 20 mg

* list specific strength of each ingredient

Alpha/Beta Blockers *Class Code = 200*

Code	Generic Name	Brand Names	Strengths Available
220	carvedilol	Coreg	3.125 mg, 6.25 mg, 12.5 mg, 25 mg
221	labetalol	Normodyne, Trandate	100 mg, 200 mg, 300 mg
222	carvedilol extended release	Coreg CR	10 mg, 20 mg, 40 mg, 80 mg

ACE Inhibitors – *Class Code = 300*

Code	Generic Name	Brand Names	Strengths Available
301	benazepril	Lotensin	5 mg, 10 mg, 20 mg, 40 mg
302	captopril	Capoten	12.5 mg, 25 mg, 50 mg, 100 mg
303	enalapril	Vasotec	2.5 mg, 5 mg, 10 mg, 20 mg
304	fosinopril	Monopril	10 mg, 20 mg, 40 mg
305	lisinopril	Zestril, Prinivil	2.5 mg, 5 mg, 10 mg, 20 mg, 30 mg, 40 mg
306	moexipril	Univasc	7.5 mg, 15 mg
307	perindopril	Aceon	2 mg, 4 mg, 8 mg
308	quinapril	Accupril	5 mg, 10 mg, 20 mg, 40 mg
309	ramipril	Altace	1.25 mg, 2.5 mg, 5 mg, 10 mg
310	trandolapril	Mavik	1 mg, 2 mg, 4 mg
311	benazepril-hydrochlorothiazide*	Lotensin HCT	5/6.25 mg, 10/12.5 mg, 20/12.5 mg, 20/25 mg
312	captopril-hydrochlorothiazide*	Capozide	25/15 mg, 25/25 mg, 50/15 mg, 50/25 mg
313	enalapril-hydrochlorothiazide*	Vaseretic	5/12.5 mg, 10/25 mg
314	fosinopril-hydrochlorothiazide*	Monopril-HCT	10/12.5 mg, 20/12.5 mg
315	lisinopril-hydrochlorothiazide*	Prinzide, Zestoretic	10/12.5 mg, 20/12.5 mg, 20/25 mg
316	moexipril-hydrochlorothiazide*	Uniretic	7.5/12.5 mg, 15/12.5 mg, 15/25 mg
317	quinapril-hydrochlorothiazide*	Accuretic	10/12.5 mg, 20/12.5 mg, 20/25 mg

* list specific strength of each ingredient

Calcium Channel Blockers – *Class Code = 400*

Code	Generic Name	Brand Names	Available Strengths
401	amlodipine	Norvasc	2.5 mg, 5 mg, 10 mg
402	diltiazem	Cardizem, Dilacor, Tiazac	30 mg, 60 mg, 90 mg, 120 mg, 180 mg, 240 mg, 300 mg, 360 mg, 420 mg
403	felodipine	Plendil	2.5 mg, 5 mg, 10 mg
404	isradipine	DynaCirc	2.5 mg, 5 mg, 10 mg
405	nicardipine	Cardene	20 mg, 30 mg, 45 mg, 60 mg
406	nifedipine	Adalat, Procardia	10 mg, 20 mg, 30 mg, 60 mg, 90 mg
407	nisoldipine	Sular	8.5 mg, 10 mg, 17 mg, 20 mg, 25.5 mg, 30 mg, 34 mg, 40 mg
408	verapamil	Calan, Isoptin, Verelan, Coer, Covera HS	40 mg, 80 mg, 100 mg 120 mg, 200 mg, 180 mg, 240 mg, 300 mg, 360 mg

ACE Inhibitor/Calcium Channel Blocker Combinations *Class Code = 300, 400*

Code	Generic Name	Brand Names	Available Strengths
410	amlodipine/benazepril*	Lotrel	2.5/10 mg, 5/10 mg, 5/20 mg, 5/40 mg, 10/20 mg, 10/40 mg
411	enalapril/felodipine *	Lexxel	5/2.5 mg, 5/5 mg
412	trandolapril/verapamil*	Tarka	1/240 mg, 2/180 mg, 2/240 mg, 4/240 mg

* list specific strength of each ingredient

Calcium Channel Blocker;Angiotensin II Receptor Blocker Combination
Class Code = 400, 600

Code	Generic Name	Brand Names	Available Strengths
420	amlodipine/valsartan*	Exforge	5/160 mg, 10/160 mg, 5/320 mg, 10/320 mg
421	amlodipine/valsartan/hydrochlorothiazide	Exforge HCT	5/160/12.5 mg, 10/160/12.5 mg, 5/160/25 mg, 10/160/25 mg, 10/320/25 mg
422	amlodipine/olmesartan	AZOR	5/20 mg, 5/40 mg, 10/20 mg, 10/40 mg

* list specific strength of each ingredient

Alpha blockers – *Class Code = 500*

Code	Generic Name	Brand Names	Available Strengths
501	doxazosin	Cardura	1 mg, 2 mg, 4 mg, 8 mg
502	prazosin	Minipress	1 mg, 2 mg, 5 mg
503	terazosin	Hytrin	1 mg, 2 mg, 5 mg, 10 mg
504	prazosin/polythiazide	Minizide	1/0.5 mg, 2/0.5 mg, 5/0.5 mg

Angiotensin II receptor antagonists (ARB) – *Class Code = 600*

Code	Generic Name	Brand Names	Available Strengths
601	candesartan	Atacand	4 mg, 8 mg, 16 mg, 32 mg
602	eprosartan	Teveten	400 mg, 600 mg
603	irbesartan	Avapro	75 mg, 150 mg, 300 mg
604	losartan	Cozaar	25 mg, 50 mg, 100 mg
605	olmesartan	Benicar	5 mg, 20 mg, 40 mg
606	telmisartan	Micardis	20 mg, 40 mg, 80 mg
607	valsartan	Diovan	40 mg, 80 mg, 160 mg, 320 mg
608	candesartan-hydrochlorothiazide	Atacand HCT	16/12.5 mg, 32/12.5 mg, 32/25 mg
609	eprosartan-hydrochlorothiazide	Teveten-HCT	600/12.5 mg, 600/25 mg
610	irbesartan-hydrochlorothiazide	Avalide	150/12.5 mg, 300/12.5 mg, 300/25 mg
611	losartan-hydrochlorothiazide	Hyzaar	50/12.5 mg, 100/12.5 mg, 100/25 mg
612	olmesartan medoxomil-hydrochlorothiazide	Benicar HCT	20/12.5 mg, 40/12.5 mg, 40/25 mg
613	telmisartan-hydrochlorothiazide	Micardis-HCT	40/12.5 mg, 80/12.5 mg, 80/25 mg
614	valsartan-hydrochlorothiazide	Diovan-HCT	80/12.5 mg, 160/12.5 mg, 160/25 mg, 320/12.5 mg, 320/25 mg
615	Azilsartan Medoxomil	Edarbi	40 mg, 80 mg

* list specific strength of each ingredient

Centrally Acting Alpha 2 blockers – *Class Code = 700*

Code	Generic Name	Brand Names	Strengths Available
701	clonidine	Catapres	0.1 mg, 0.2 mg, 0.3 mg
702	clonidine topical patch	Catapres TTS	0.1 mg, 0.2 mg, 0.3 mg
703	guanabenz	Wytensin	4 mg, 8 mg
704	guanfacine	Tenex	1 mg, 2 mg
705	methyldopa	Aldomet	250 mg, 500 mg
706	methyldopa-hydrochlorothiazide	Aldoril	250/15 mg, 250/25 mg
707	clonidine-chlorthalidone	Clorpres	0.1/15 mg, 0.2/15 mg, 0.3/15 mg

* list specific strength of each ingredient

Peripheral Adrenergic Blocking Agents – *Class Code = 800*

Code	Generic Name	Brand Names	Strengths Available
801	reserpine		0.1 mg, 0.25 mg
802	reserpine-chlorthalidone	Demi-Regroton	
803	reserpine-chlorothiazide	Diupres	
804	reserpine-hydrochlorothiazide	Hydropres	0.125/25 mg, 0.125/50 mg
805	Ser Ap Es		

Vasodilators – *Class Code = 900*

Code	Generic Name	Brand Names	Strengths Available
901	hydralazine	Apresoline	10 mg, 25 mg, 50 mg, 100 mg
902	isosorbide dinitrate	Isordil	2.5 mg, 5 mg, 10 mg, 20 mg, 30 mg, 40 mg
903	isosorbide mononitrate	Imdur	10 mg, 20 mg, 30 mg, 60 mg, 120 mg
904	minoxidil	Loniten	2.5 mg, 10 mg
905	hydralazine-hydrochlorothiazide		25/25 mg, 50/50 mg, 100/50 mg
906	isosorbide dinitrate-hydralazine	BiDil	20/37.5 mg

* list specific strength of each ingredient

Aldosterone Receptor Blockers – *Class Code = 1000*

Code	Generic Name	Brand Names	Strengths Available
1001	eplerenone	Inspra	25 mg, 50 mg
1002	spironolactone	Aldactone	25 mg, 50 mg, 100 mg

* list specific strength of each ingredient

Direct Renin Inhibitor – *Class Code = 1100*

Code	Generic Name	Brand Names	Strengths Available
1101	aliskiren	Tekturna	150 mg, 300 mg
1102	aliskiren-hydrochlorothiazide	Tekturna HCT	150/12.5 mg, 150/25 mg, 300/12.5 mg, 300/25 mg
1103	aliskiren-valsartan	Valturna	150/160 mg, 300/320 mg

ICARE STUDY
Drug Codes for Hyperglycemic Agents

Insulins– *Class Code = 2000*

Code	Generic Name	Brand Names	Strengths Available
2001	Aspart	Novolog	100U/ml
2002	Aspart Protamine	Novolog Mix	70/30 – 100U/ml
2003	Detemir	Levemir	100U/ml
2004	Glargine	Lantus	100U/ml
2005	Glulisine	Apidra	100U/ml
2006	Lispro	Humalog	100U/ml
2007	Lispro Protamine	Humalog Mix	50/50, 75/25
2008	Isophane	Humulin N, Novolin N	100U/ml
2009	Regular	Humulin R, Novolin R	100U/ml, 500U/ml
2010	Regular:Isophane	NPH	Novolin 70/30, Humuilin 50/50, Humulin 70/30

Alpha-glucosidase inhibitors– *Class Code = 2100*

Code	Generic Name	Brand Names	Strengths Available
2101	Acarbose	Precose	25mg. 50mg, 100mg
2102	Miglitol	Glyset	25mg, 50mg, 100mg

Amylin analogues– *Class Code = 2200*

Code	Generic Name	Brand Names	Strengths Available
2201	Pramlintide	Symlin	60 and 120

Biguanides– *Class Code = 2300*

Code	Generic Name	Brand Names	Strengths Available
2301	Metformin	Glucophage, Fortamet, Appformin, Glumetza	500mg, 750mg, 850mg, 1000mg

Dipeptidyl peptidase 4 inhibitor – *Class Code = 2400*

Code	Generic Name	Brand Names	Strengths Available
2401	Sitagliptin	Januvia	25mg, 50mg, 100mg
2402	Sitagliptin/simvastatin (OFF-MARKET)	Juvasync	100-10mg, 100-20mg, 100-40mg
2403	Saxagliptin	Onglyza	2.5mg, 5mg
2404	Linagliptin	Tradjenta	5mg

Biguanide/dipeptidyl peptidase 4 inibitor– *Class Code = 2500*

Code	Generic Name	Brand Names	Strengths Available
2501	Sitagliptin/metformin	Janumet	50/500mg, 50/1000mg 100/1000mg
2502	Sitagliptin/metformin XR	Janumet XR	50/500mg, 50/1000mg 100/1000mg
2503	Linagliptin-metformin	Jentadueto	2.5/500mg, 2.5/850mg, 2.5/1000mg
2504	Sexagliptin/metformin XR	Kombiglyze	2.5/1000mg 5/500mg, 5/1000mg

meglitinides– *Class Code = 2600*

Code	Generic Name	Brand Names	Strengths Available
2601	Nateglinide	Starlix	60mg, 120mg
2602	Repaglinide	Prandin	0.5mg, 1mg, 2mg

Biguanide/meglitinide– *Class Code = 2700*

Code	Generic Name	Brand Names	Strengths Available
2701	Metformin/repaglinide	Prandimet	500/1mg, 500/2mg

Thiazoladinediones– *Class Code = 2800*

Code	Generic Name	Brand Names	Strengths Available
2801	Pioglitazone	Actos	15mg, 30mg, 45mg
2802	Rosiglitazone	Avandia	2mg, 4mg, 8mg

Biguanide/Thiazoladinediones– *Class Code = 2900*

Code	Generic Name	Brand Names	Strengths Available
2901	Pioglitazone/metformin	ActoPlus	15/500mg, 15/850mg 15/1000mg, 30/1000mg,
2902	Rosiglitazone/metformin	Avandamet	2/500mg, 2/1000mg, 4/500mg, 4/1000mg

Incretin Mimetics– *Class Code = 3000*

Code	Generic Name	Brand Names	Strengths Available
3001	Exanatide	Byetta	5mcg, 10mcg
3002	Liraglutide	Victoza	18mg/3ml

Sulfonylureas– *Class Code = 3100*

Code	Generic Name	Brand Names	Strengths Available
3101	Chlorpropamide	Diabinese	100mg, 250mg
3102	Glimeperide	Amaryl	1mg, 2mg, 4mg
3103	Glipizide	Glucotrol	5mg, 10mg
3104	Glipizide XR	Glucotrol XR	2.5mg, 5mg, 10mg
3105	Glyburide	Diabeta, Micronase	1.25mg, 2.5mg, 5mg
3106	Glyburide – micronized	Glynase	1.5mg, 3mg, 6mg
3107	Tolazamide	Tolinase	250mg, 500mg
3108	Tolbutaminde	Orinase	500mg

Sulfonylurea/biguanide– *Class Code = 3200*

Code	Generic Name	Brand Names	Strengths Available
3201	Glipizide/metformin	Metaglip	2.5/250mg, 2.5/500mg, 5/500mg
3202	Glyburide/metformin	Glucovance	1.25/250mg, 2.5/500mg, 5/500mg

Sulfonylureas/thiazoladinediones – *Class Code = 3300*

Code	Generic Name	Brand Names	Strengths Available
3301	Glimepiride/pioglitazone	Duetact	2/30mg, 4/30mg
3302	Glimepiride/rosiglitazone	Avandaryl	1/4mg, 2/4mg, 4/4mg,2/8mg, 4/8mg

Sodium-glucose co-transporter 2 (SGLT2) – *Class Code = 3400*

Code	Generic Name	Brand Names	Strengths Available
3401	Canagliflozin	Invokana	100mg, 300mg
3402	Dapagliflozin	Farxiga	5mg, 10mg

ICARE STUDY

Drug Codes for Cholesterol Agents

HMG – CoA Reductase inhibitors "statins"– *Class Code = 4000*

Code	Generic Name	Brand Names	Strengths Available
4001	Atorvastatin	Lipitor	10mg, 20mg, 40mg, 80mg
4002	Rosuvastatin	Crestor	5mg, 10mg, 20mg, 40mg
4003	Fluvastatin, Fluvastatin XL	Lescol, Lescol XL	20mg, 40mg, 80mg
4004	Lovastatin, Lovastatin XR	Mevacor, Altoprev	10mg, 20mg, 40mg, 60mg
4005	Pravastatin	Pravachol	10mg, 20mg, 40mg, 80mg
4006	Simvastatin	Zocor	10mg, 20mg, 40mg, 80mg
4007	Pitavastatin	Livalo	1mg, 2mg, 4mg

HMG – CoA Reductase Inh (statin)/Niacin combination– *Class Code = 4100*

Code	Generic Name	Brand Names	Strengths Available
4101	Lovastatin/Niacin	Advicor	20/500mg, 20/750mg, 20/1000mg, 40/1000mg
4102	Simvastatin/Niacin	Simcor	20/500mg, 40/500mg, 20/750mg, 20/1000mg, 40/1000mg

HMG–CoA Reductase Inh (statin)/Cholesterol Absorption Inh combination – *Class Code = 4200*

Code	Generic Name	Brand Names	Strengths Available
4201	Simvastatin/exetimibe	Vytorin	10/10mg, 20/10mg, 40/10mg, 80/10mg

HMG–CoA Reductase Inh (statin)/Calcium Channel Blocker combination – *Class Code = 4300*

Code	Generic Name	Brand Names	Strengths Available
4301	Atorvastatin/amlodipine	Caduet	10/2.5mg, 20/2.5mg, 40/2.5mg, 10/5mg, 20/5mg, 40/5mg, 80/5mg, 10/10mg, 20/10mg, 40/10mg, 80/10mg

HMG–CoA Reductase Inh (statin)/Dipeptidyl Peptidase-4 Inh combination – *Class Code = 4400*

Code	Generic Name	Brand Names	Strengths Available
4401	Simvastatin/Sitagliptan	Juvisync	10/100mg, 20/100mg, 40/100mg

Niacin (Rx) – *Class Code = 4500*
*Many over –the-counter products available. Only document prescribed products

Code	Generic Name	Brand Names	Strengths Available
4501	Niacin	Niaspan	500mg, 750mg, 1000mg

Cholesterol Absorption Inh combination – *Class Code = 4600*

Code	Generic Name	Brand Names	Strengths Available
4601	Exetimibe	Zetia	10mg

Omega - 3 Fatty acids – *Class Code = 4700*
*Many over –the-counter products available. Only document prescribed products

Code	Generic Name	Brand Names	Strengths Available
4701	Omega – 3 fatty Acids	Lovaza	1 gr

Bile Acid Sequestrants – *Class Code = 4800*

Code	Generic Name	Brand Names	Strengths Available
4801	Cholestyramine,	Questran,	4 gr
4802	Cholesteramine light	Questran Light, Prevalite	4 gr
4803	Colestipol	Colestid	1 gr tab, 5 gr granules
4804	Colesevelam	WelChol	625mg tab, 3.75g powder

Fibric Acids – *Class Code = 4900*

Code	Generic Name	Brand Names	Strengths Available
4901	Gemfibrozil	Lopid	600mg
4902	Fenofibrate	Lipofen, Lofibra, Tricor, Triglide	40mg, 48mg, 50mg, 54mg, 120mg, 145mg, 160mg
4903	Fenofibrate - Micronized	Lofibra, Antara	43mg, 67mg, 130mg, 134mg, 200mg

ICARE STUDY

Drug Codes for Antiplatelet and Anticoagulant Agents

Anticoagulants – *Class Code = 5000*

Code	Generic Name	Brand Names	Strengths Available
5001	Warfarin	Coumadin, Jantoven	1mg, 2mg, 2.5mg, 3mg, 4mg, 5mg, 6mg, 7.5mg, 10mg
5002	Enoxaparin	Lovenox	30mg, 40mg, 60mg, 80mg, 100mg, 120mg, 150mg, 300mg
5003	Daltaperin	Fragmin	2500U, 5000U, 7500U, 10,000U, 12,500U, 15,000U, 18,000U, 25,000U

Adenosine diphosphate inhibitor (thienopyridine) – *Class Code = 5100*

Code	Generic Name	Brand Names	Strengths Available
5101	Clopidogrel	Plavix	75mg, 300mg
5102	Ticagrelor	Brilinta	90mg
5103	Prasugrel	Effient	5mg, 10mg
5104	Ticlopidine	Ticlid	250mg

Factor Xa inhibitors – *Class Code = 5200*

Code	Generic Name	Brand Names	Strengths Available
5201	Rivaroxaban	Xarelto	10mg, 15mg, 20mg
5202	Apixaban	Eliquis	2.5mg, 5mg

Direct Thrombin inhibitors – *Class Code = 5300*

Code	Generic Name	Brand Names	Strengths Available
5301	Dabigatran	Pradaxa	75mg, 150mg

Salicylates – *Class Code = 5400*

Code	Generic Name	Brand Names	Strengths Available
5401	Aspirin	Ecotrin, Bufferin, Aspergum	81mg, 227mg, 325mg, 500mg, 650mg

Drug Codes for Asthma

Inhaled Corticosteroids – *Class Code = 6100*

Code	Generic Name	Brand Names	Strengths Available
6101	Beclomethasone	Beclovent, QVAR	42 mcg, 40 mcg, 80 mcg
6102	Budesonide	Pulmicort Flexhaler	90 mcg, 180 mcg
6103	Ciclesonide	Alvesco	80 mcg, 160 mcg
6104	Flunisolide	Aerobid	.25 mg
6105	Fluticasone Propionate	Flovent Diskus, Flovent, Flovent HFA	50 mcg, 100 mcg, 250 mcg, 44 mcg, 110 mcg, 220 mcg
6106	Mometasone	Asmanex Twisthaler/ Twist	220 mcg, 110 mcg

Long-Acting Beta-Agonists – *Class Code = 6200*

Code	Generic Name	Brand Names	Strengths Available
6201	Arformoterol Tartrate	Brovana	15 mcg
6202	Formoterol Fumarate	Foradil Aerolizer Inhaler	12 mcg
6203	Indacaterol Maleate	Arcapta Neohaler	75 mcg
6204	Salmeterol Xinafoate	Serevent Diskus	50 mcg

Leukotriene Modifiers -- *Class Code = 6300*

Code	Generic Name	Brand Names	Strengths Available
6301	Montelukast	Singulair	4 mg, 5 mg, 10 mg
6302	Zafirlukast	Accolate	10 mg, 20 mg
6303	Zileuton	Zyflor CR, Zyflo	600 mg, 1.2 g

Theophyllines (SR) – *Class Code = 6400*

Code	Generic Name	Brand Names	Strengths Available
6401	Theophylline (12 hr)	Theo-Dur, TheoCap	100 mg, 200 mg, 300 mg, 450 mg
6402	Theophylline (24 hr)	Theo-Time, Theo-24, Uniphyl, Theochron, Quibron-T	100 mg, 200 mg, 300 mg, 400 mg, 600 mg

Combinations- Inhaled Corticoidsteroid and LABA – *Class Code = 6500*

Code	Generic Name	Brand Names	Available Strengths
6501	Fluticasone/ Salmeterol	Advair Diskus, Advair HFA	100mcg-50 mcg, 250 mcg-50 mcg, 500 mcg-50 mcg, 45 mcg-21 mcg, 115 mcg-21 mcg, 230 mcg-21 mcg
6502	Fluticasone/ Vilanterol	BREO ELLIPTA	100 mcg-25 mcg
6503	Mometasone/ Formoterol	Dulera	100 mcg-5 mcg, 200 mcg-5 mcg
6504	Budesonide/ Formoterol	Symbicort	80 mcg-4.5 mcg, 160 mcg-4.5 mcg

References

1. Roger VL, Go AS, Lloyd-Jones DM, et al. Heart disease and stroke statistics--2011 update: a report from the American Heart Association. *Circulation.* Feb 1 2011;123(4):e18-e209.

2. Smith SC, Jr., Allen J, Blair SN, et al. AHA/ACC guidelines for secondary prevention for patients with coronary and other atherosclerotic vascular disease: 2006 update: endorsed by the National Heart, Lung, and Blood Institute. *Circulation.* May 16 2006;113(19):2363-2372.

3. Brown DL, Boden-Albala B, Langa KM, et al. Projected costs of ischemic stroke in the United States. *Neurology.* Oct 24 2006;67(8):1390-1395.

4. Keyhani S, Scobie JV, Hebert PL, McLaughlin MA. Gender disparities in blood pressure control and cardiovascular care in a national sample of ambulatory care visits. *Hypertension.* Apr 2008;51(4):1149-1155.

5. Fang G, Robinson J, Carter BL, Brookhart MA. Regional variation and age disparities in the use of evidence-based therapies in the elderly after acute myocardial infarction in the US. JAMA (in review).

6. Milchak JL, Carter BL, James PA, Ardery G. Measuring adherence to practice guidelines for the management of hypertension: an evaluation of the literature. *Hypertension.* Dec 2004;44(5):602-608.

7. Cabana MD, Rand CS, Powe NR, et al. Why don't physicians follow clinical practice guidelines? A framework for improvement. *JAMA.* 1999;282(15):1458-1465.

8. Qureshi AI, Suri MF, Guterman LR, Hopkins LN. Ineffective secondary prevention in survivors of cardiovascular events in the US population: report from the Third National Health and Nutrition Examination Survey. *Arch Intern Med.* Jul 9 2001;161(13):1621-1628.

9. Rodondi N, Peng T, Karter AJ, et al. Therapy modifications in response to poorly controlled hypertension, dyslipidemia, and diabetes mellitus. *Ann Intern Med.* Apr 4 2006;144(7):475-484.

10. Higashi T, Shekelle PG, Solomon DH, et al. The quality of pharmacologic care for vulnerable older patients. *Ann Intern Med.* May 4 2004;140(9):714-720.

11. Levy BT, Daly JM, Xu Y, Ely JW. Mailed Fecal Immunochemical Tests Plus Educational Materials to Improve Colon Cancer Screening Rates in Iowa Research Network (IRENE) Practices. *J Am Board Fam Med.* Jan 2012;25(1):73-82.

12. Levy BT, Dawson J, Hartz AJ, James PA. Colorectal cancer testing among patients cared for by Iowa family physicians. *Am J Prev Med.* Sep 2006;31(3):193-201.

13. Choudhry NK, Fischer MA, Avorn J, et al. The implications of therapeutic complexity on adherence to cardiovascular medications. *Arch Intern Med.* May 9 2011;171(9):814-822.

14. Chan PS, Oetgen WJ, Buchanan D, et al. Cardiac performance measure compliance in outpatients: the American College of Cardiology and National Cardiovascular Data Registry's PINNACLE (Practice Innovation And Clinical Excellence) program. *Journal of the American College of Cardiology.* Jun 29 2010;56(1):8-14.

15. Rosenthal TC. Advancing Medical Homes: Evidence-based literature review to inform health policy. http://www.ahec.buffalo.edu.

16. Audet AM, Davis K, Schoenbaum SC. Adoption of patient-centered care practices by physicians: results from a national survey. *Arch Intern Med.* Apr 10 2006;166(7):754-759.

17. Feifer C, Nemeth L, Nietert PJ, et al. Different paths to high-quality care: three archetypes of top-performing practice sites. *Ann Fam Med.* May-Jun 2007;5(3):233-241.
18. O'Malley AS, Forrest CB, Politzer RM, Wulu JT, Shi L. Health center trends, 1994-2001: what do they portend for the federal growth initiative? *Health Aff (Millwood).* Mar-Apr 2005;24(2):465-472.
19. Phelan EA, Balderson B, Levine M, et al. Delivering Effective Primary Care to Older Adults: A Randomized, Controlled Trial of the Senior Resource Team at Group Health Cooperative. *J Am Geriatr Soc.* Nov 2007;55(11):1748-1756.
20. Rosenthal T, Campbell-Heider N. The Rural Health Care Team. In: Geyman JP NT, Hart G, ed. *Textbook of Rural Health Care*. New York: Mcgraw-Hill; 2001.
21. Grumbach K, Bodenheimer T. Can health care teams improve primary care practice? *Jama.* Mar 10 2004;291(10):1246-1251.
22. Peek CJ, Baird MA, Coleman E. Primary care for patient complexity, not only disease. *Fam Syst Health.* Dec 2009;27(4):287-302.
23. Wagner EH, LaCroix AZ, Grothaus L, et al. Preventing disability and falls in older adults: a population-based randomized trial. *Am J Public Health.* Nov 1994;84(11):1800-1806.
24. Wagner EH, Austin BT, Von Korff M. Organizing care for patients with chronic illness. *Milbank Q.* 1996;74(4):511-544.
25. Wagner EH. Chronic disease management: what will it take to improve care for chronic illness? *Eff Clin Pract.* Aug-Sep 1998;1(1):2-4.
26. Wagner EH, Austin BT, Davis C, Hindmarsh M, Schaefer J, Bonomi A. Improving chronic illness care: translating evidence into action. *Health Aff (Millwood).* Nov-Dec 2001;20(6):64-78.
27. Wagner EH, Glasgow RE, Davis C, et al. Quality improvement in chronic illness care: a collaborative approach. *Jt Comm J Qual Improv.* Feb 2001;27(2):63-80.
28. Bodenheimer T, Wagner EH, Grumbach K. Improving primary care for patients with chronic illness. *Jama.* Oct 9 2002;288(14):1775-1779.
29. Bodenheimer T, Wagner EH, Grumbach K. Improving primary care for patients with chronic illness: the chronic care model, Part 2. *Jama.* Oct 16 2002;288(15):1909-1914.
30. Coleman EA, Grothaus LC, Sandhu N, Wagner EH. Chronic care clinics: a randomized controlled trial of a new model of primary care for frail older adults. *J Am Geriatr Soc.* Jul 1999;47(7):775-783.
31. McCulloch DK, Price MJ, Hindmarsh M, Wagner EH. A population-based approach to diabetes management in a primary care setting: early results and lessons learned. *Eff Clin Pract.* Aug-Sep 1998;1(1):12-22.
32. Simon GE, VonKorff M, Rutter C, Wagner E. Randomised trial of monitoring, feedback, and management of care by telephone to improve treatment of depression in primary care. *Bmj.* Feb 26 2000;320(7234):550-554.
33. Feifer C, Ornstein SM, Nietert PJ, Jenkins RG. System supports for chronic illness care and their relationship to clinical outcomes. *Top Health Inf Manage.* Nov 2001;22(2):65-72.
34. Leveille SG, Wagner EH, Davis C, et al. Preventing disability and managing chronic illness in frail older adults: a randomized trial of a community-based partnership with primary care. *J Am Geriatr Soc.* Oct 1998;46(10):1191-1198.
35. Cowper PA, Weinberger M, Hanlon JT, et al. The cost-effectiveness of a clinical pharmacist intervention among elderly outpatients. *Pharmacotherapy.* Mar-Apr 1998;18(2):327-332.

36. Hammond RW, Schwartz AH, Campbell MJ, et al. Collaborative drug therapy management by pharmacists--2003. *Pharmacotherapy.* Sep 2003;23(9):1210-1225.

37. **Carter BL**, Helling DK. Ambulatory care pharmacy services: has the agenda changed? *Ann Pharmacother.* Jun 2000;34(6):772-787.

38. Carter BL, Zillich AJ, Elliott WJ. How pharmacists can assist physicians with controlling blood pressure. *J Clin Hypertens (Greenwich).* Jan-Feb 2003;5(1):31-37.

39. Chrischilles EA, Carter BL, Lund BC, et al. Evaluation of the Iowa Medicaid pharmaceutical case management program. *J Am Pharm Assoc (Wash DC).* May-Jun 2004;44(3):337-349.

40. Hanlon JT, Weinberger M, Samsa GP, et al. A randomized, controlled trial of a clinical pharmacist intervention to improve inappropriate prescribing in elderly outpatients with polypharmacy. *Am J Med.* Apr 1996;100(4):428-437.

41. Malone DC, Carter BL, Billups SJ, et al. An economic analysis of a randomized, controlled, multicenter study of clinical pharmacist interventions for high-risk veterans: the IMPROVE study. Impact of Managed Pharmaceutical Care Resource Utilization and Outcomes in Veterans Affairs Medical Centers. *Pharmacotherapy.* Oct 2000;20(10):1149-1158.

42. Malone DC, Carter BL, Billups SJ, et al. Can clinical pharmacists affect SF-36 scores in veterans at high risk for medication-related problems? *Med Care.* 2001;39(2):113-122.

43. Lipton HL, Byrns PJ, Soumerai SB, Chrischilles EA. Pharmacists as agents of change for rational drug therapy. *Int J Technol Assess Health Care.* Summer 1995;11(3):485-508.

44. Pauley TR, Magee MJ, Cury JD. Pharmacist-managed, physician-directed asthma management program reduces emergency department visits. *Ann Pharmacother.* Jan 1995;29(1):5-9.

45. Tillman DJ, Charland SL, Witt DM. Effectiveness and economic impact associated with a program for outpatient management of acute deep vein thrombosis in a group model health maintenance organization. *Arch Intern Med.* 2000;160(19):2926-2932.

46. Tsuyuki RT, Johnson JA, Teo KK, et al. A randomized trial of the effect of community pharmacist intervention on cholesterol risk management: the Study of Cardiovascular Risk Intervention by Pharmacists (SCRIP). *Arch Intern Med.* 2002;162(10):1149-1155.

47. Stewart S, Pearson S, Horowitz JD. Effects of a home-based intervention among patients with congestive heart failure discharged from acute hospital care. *Arch Intern Med.* 1998;158(10):1067-1072.

48. Ellis SL, **Carter BL**, Malone DC, et al. Clinical and economic impact of ambulatory care clinical pharmacists in management of dyslipidemia in older adults: the IMPROVE study. Impact of Managed Pharmaceutical Care on Resource Utilization and Outcomes in Veterans Affairs Medical Centers. *Pharmacotherapy.* 2000;20(12):1508-1516.

49. Gattis WA, Hasselblad V, Whellan DJ, O'Connor CM. Reduction in heart failure events by the addition of a clinical pharmacist to the heart failure management team: results of the Pharmacist in Heart Failure Assessment Recommendation and Monitoring (PHARM) Study. *Arch Intern Med.* 1999;159(16):1939-1945.

50. Murray MD, Young J, Hoke S, et al. Pharmacist intervention to improve medication adherence in heart failure: a randomized trial. *Ann Intern Med.* May 15 2007;146(10):714-725.

51. Wilt VM, Gums JG, Ahmed OI, Moore LM. Outcome analysis of a pharmacist-managed anticoagulation service. *Pharmacotherapy.* 1995;15(6):732-739.

52. Witt DM, Sadler MA, Shanahan RL, Mazzoli G, Tillman DJ. Effect of a centralized clinical pharmacy anticoagulation service on the outcomes of anticoagulation therapy. *Chest.* May 2005;127(5):1515-1522.
53. Merenich JA, Olson KL, Delate T, Rasmussen J, Helling DK, Ward DG. Mortality reduction benefits of a comprehensive cardiac care program for patients with occlusive coronary artery disease. *Pharmacotherapy.* Oct 2007;27(10):1370-1378.
54. Straka RJ, Taheri R, Cooper SL, Smith JC. Achieving cholesterol target in a managed care organization (ACTION) trial. *Pharmacotherapy.* Mar 2005;25(3):360-371.
55. Olson KL, Rasmussen J, Sandhoff BG, Merenich JA. Lipid management in patients with coronary artery disease by a clinical pharmacy service in a group model health maintenance organization. *Arch Intern Med.* Jan 10 2005;165(1):49-54.
56. Rothman RL, Malone R, Bryant B, et al. A randomized trial of a primary care-based disease management program to improve cardiovascular risk factors and glycated hemoglobin levels in patients with diabetes. *Am J Med.* Mar 2005;118(3):276-284.
57. Yamada C, Johnson JA, Robertson P, Pearson G, Tsuyuki RT. Long-term impact of a community pharmacist intervention on cholesterol levels in patients at high risk for cardiovascular events: extended follow-up of the second study of cardiovascular risk intervention by pharmacists (SCRIP-plus). *Pharmacotherapy.* Jan 2005;25(1):110-115.
58. Zillich AJ, McDonough RP, Carter BL, Doucette WR. Influential characteristics of physician/pharmacist collaborative relationships. *Ann Pharmacother.* May 2004;38(5):764-770.
59. Zillich AJ, Milchak JL, Carter BL, Doucette WR. Utility of a questionnaire to measure physician-pharmacist collaborative relationships. *J Am Pharm Assoc (2003).* Jul-Aug 2006;46(4):453-458.
60. Zillich AJ, Sutherland JM, Kumbera PA, Carter BL. Hypertension outcomes through blood pressure monitoring and evaluation by pharmacists (HOME study). *J Gen Intern Med.* Dec 2005;20(12):1091-1096.
61. Carter BL, Bergus GR, Dawson JD, et al. A Cluster Randomized Trial to Evaluate Physician/Pharmacist Collaboration to Improve Blood Pressure Control. *J Clin Hypertens (Greenwich).* Apr 2008;10(4):260-271.
62. Santschi V, Chiolero A, Burnand B, Colosimo AL, Paradis G. Impact of pharmacist care in the management of cardiovascular disease risk factors: a systematic review and meta-analysis of randomized trials. *Arch Intern Med.* Sep 12 2011;171(16):1441-1453.
63. Cooley WC, McAllister JW, Sherrieb K, Clark RE. The Medical Home Index: development and validation of a new practice-level measure of implementation of the Medical Home model. *Ambul Pediatr.* Jul-Aug 2003;3(4):173-180.
64. The Medical Home Index: Adult. Center for Medical Home Improvement. http://www.medicalhomeimprovement.org. Accessed September 13, 2012.
65. Designing theoretically-informed implementation interventions. *Implement Sci.* 2006;1:4.
66. Zillich AJ, Doucette WR, **Carter BL**, Kreiter CD. Development and initial validation of an instrument to measure physician-pharmacist collaboration from the physician perspective. *Value Health.* Jan-Feb 2005;8(1):59-66.
67. McDonough RP, Doucette WR. Developing collaborative working relationships between pharmacists and physicians. *J Amer Pharmaceut Assoc.* 2001;8:55-69.

68. Francis JJ, Grimshaw JM, Zwarenstein M, et al. Testing a TheoRY-inspired MEssage (TRY-ME): a sub-trial within the Ontario Printed Educational Message (OPEM) trial. *Implement Sci.* Nov 26 2007;2(1):39.

69. Grimshaw JM, Zwarenstein M, Tetroe JM, et al. Looking inside the black box: a theory-based process evaluation alongside a randomised controlled trial of printed educational materials (the Ontario printed educational message, OPEM) to improve referral and prescribing practices in primary care in Ontario, Canada. *Implement Sci.* Nov 26 2007;2(1):38.

70. Carter BL, Clarke W, Ardery G, et al. A cluster-randomized effectiveness trial of a physician-pharmacist collaborative model to improve blood pressure control. *Circ Cardiovasc Qual Outcomes.* Jul 2010;3(4):418-423.

71. Prochaska JO, DiClemente CC, Velicer WF, Rossi JS. Standardized, individualized, interactive, and personalized self-help programs for smoking cessation. *Health Psychol.* Sep 1993;12(5):399-405.

72. Prochaska JO. Strong and weak principles for progressing from precontemplation to action on the basis of twelve problem behaviors. *Health Psychol.* Jan 1994;13(1):47-51.

73. Prochaska JO, Redding CA, Harlow LL, Rossi JS, Velicer WF. The transtheoretical model of change and HIV prevention: a review. *Health Educ Q.* Winter 1994;21(4):471-486.

74. Prochaska JO, Velicer WF, Rossi JS, et al. Stages of change and decisional balance for 12 problem behaviors. *Health Psychol.* Jan 1994;13(1):39-46.

75. Rollnick S, Heather N. Negotiating behaviour change in medical settings: the development of brief motivational interviewing. Journal of Mental Health. 1992;1:25-38.

76. Doherty SC, Steptoe A, Rink E, Kendrick T, Hilton S. Readiness to change health behaviours among patients at high risk of cardiovascular disease. *J Cardiovasc Risk.* Jun 1998;5(3):147-153.

77. Carter BL, Ardery G, Dawson JD, et al. Physician and pharmacist collaboration to improve blood pressure control. *Arch Intern Med.* Nov 23 2009;169(21):1996-2002.

78. Levy BT, Hartz A, Woodworth G, Xu Y, Sinift S. Interventions to improving osteoporosis screening: an Iowa Research Network (IRENE) study. *J Am Board Fam Med.* Jul-Aug 2009;22(4):360-367.

79. Levy BT, Joshi M, Xu Y, Daly J, James PA. Perceptions of Iowa family physicians regarding colorectal cancer screening. *Med Care.* Sep 2008;46(9 Suppl 1):S103-108.

80. Daly JM, Ely JW, Levy BT, et al. Primary care clinicians' perspectives on management of skin and soft tissue infections: an Iowa Research Network study. *J Rural Health.* Summer 2011;27(3):319-328.

81. Daly JM, Hartz AJ, Xu Y, et al. An assessment of attitudes, behaviors, and outcomes of patients with type 2 diabetes. *J Am Board Fam Med.* May-Jun 2009;22(3):280-290.

82. Daly JM, Levy BT, Merchant ML, Wilbur J. Mailed fecal-immunochemical test for colon cancer screening. *J Community Health.* Jun 2010;35(3):235-239.

83. Daly JM, Merchant ML, Levy BT. Colorectal cancer screening. *Am J Nurs.* Oct 2009;109(10):60-62.

84. Daly JM, Xu Y, Ely JW, Levy BT. A Randomized Colorectal Cancer Screening Intervention Trial in the Iowa Research Network (IRENE): Study Recruitment Methods and Baseline Results. *J Am Board Fam Med.* Jan 2012;25(1):63-72.

85. Ellis SL, Billups SJ, Malone DC, et al. Types of interventions made by clinical pharmacists in the IMPROVE study. Impact of Managed Pharmaceutical Care on Resource Utilization and Outcomes in Veterans Affairs Medical Centers. *Pharmacotherapy.* Apr 2000;20(4):429-435.

86. McCall N, Cromwell J. Results of the Medicare Health Support disease-management pilot program. *N Engl J Med.* Nov 3 2011;365(18):1704-1712.
87. James PA, Li P, Ward MM. Myocardial infarction mortality in rural and urban hospitals: rethinking measures of quality of care. *Ann Fam Med.* Mar-Apr 2007;5(2):105-111.
88. **Carter BL**, Chrischilles EA, Scholz D, Hayase N, Bell N. Extent of services provided by pharmacists in the Iowa Medicaid Pharmaceutical Case Management program. *J Am Pharm Assoc.* Jan-Feb 2003;43(1):24-33.
89. Carter BL, Helling DK, Jones ME, Moessner H, Waterbury CA, Jr. Evaluation of family physician prescribing: influence of the clinical pharmacist. *Drug Intell Clin Pharm.* 1984;18(10):817-821.
90. Carter BL, Malone DC, Billups SJ, et al. Interpreting the findings of the IMPROVE study. *Am J Health Syst Pharm.* 2001;58(14):1330-1337.
91. **Carter BL**, Barnette DJ, Chrischilles E, Mazzotti GJ, Asali ZJ. Evaluation of hypertensive patients after care provided by community pharmacists in a rural setting. *Pharmacotherapy.* 1997;17(6):1274-1285.
92. **Carter BL**, Malone DC, Valuck RJ, Barnette DJ, Sintek CD, Billups SJ. The IMPROVE study: background and study design. Impact of Managed Pharmaceutical Care on Resource Utilization and Outcomes in Veterans Affairs Medical Centers. *Am J Health Syst Pharm.* 1998;55(1):62-67.
93. Von Muenster SJ, Carter BL, Weber CA, et al. Description of pharmacist interventions during physician-pharmacist co-management of hypertension. *Pharm World Sci.* Jan 2008;30(1):128-135.
94. Ardery G, Carter BL, Milchak JL, et al. Explicit and implicit evaluation of physician adherence to hypertension guidelines. *J Clin Hypertens (Greenwich).* Feb 2007;9(2):113-119.
95. Milchak JL, Carter BL, Ardery G, et al. Development of explicit criteria to measure adherence to hypertension guidelines. *J Hum Hypertens.* Jun 2006;20(6):426-433.
96. Milchak JL, Carter BL, Ardery G, Dawson JD, Harmston M, Franciscus CL. Physician adherence to blood pressure guidelines and its effect on seniors. *Pharmacotherapy.* Jul 2008;28(7):843-851.
97. **Milchak JL**, **Carter BL**, **Bakris GL**, **Black HR**, **Jones DW**, Zillich AJ. Selection of explicit criteria for a JNC-7 guideline adherence tool. *American Journal of Hypertension.* 2004;17:144A.
98. **James PA**, Cowan TM, Graham RP. Patient-centered clinical decisions and their impact on physician adherence to clinical guidelines. *J Fam Pract.* 1998;46(4):311-318.
99. **James PA**, Cowan TM, Graham RP, Jaen CR, Majeroni BA, Schwartz JS. Heart Failure in Primary Care; Measuring the Quality of Care. *J Fam Pract.* 1999;48:790-798.
100. **James PA**, Cowan TM, Graham RP, Majeroni BA. Family physicians' attitudes about and use of clinical practice guidelines. *J Fam Pract.* 1997;45(4):341-347.
101. **James PA**, Cowan TM, Graham RP, Majeroni BA, Fox CH, Jaen CR. Using a clinical practice guideline to measure physician practice: translating a guideline for the management of heart failure. *J Am Board Fam Pract.* 1997;10(3):206-212.
102. Carter BL, Butler CD, Rogers JC, Holloway RL. Evaluation of physician decision making with the use of prior probabilities and a decision-analysis model. *Arch Fam Med.* May 1993;2(5):529-534.
103. Carter BL, Hartz A, Bergus G, et al. Relationship between physician knowledge of hypertension and blood pressure control. *J Clin Hypertens (Greenwich).* Jul 2006;8(7):481-486.
104. Anon. http://www.iom.edu/Reports/2009/ComparativeEffectivenessResearchPriorities.aspx Accessed March 14, 2011.

105. Vital signs: prevalence, treatment, and control of high levels of low-density lipoprotein cholesterol --- United States, 1999--2002 and 2005--200. *MMWR Morb Mortal Wkly Rep.* Feb 4 2011;60(4):109-114.
106. Vital signs: prevalence, treatment, and control of hypertension --- United States, 1999--2002 and 2005--2008. *MMWR Morb Mortal Wkly Rep.* Feb 4 2011;60(4):103-108.
107. Nkansah N, Mostovetsky O, Yu C, et al. Effect of outpatient pharmacists' non-dispensing roles on patient outcomes and prescribing patterns. *Cochrane Database Syst Rev.* 2010(7):CD000336.
108. Morrison F, Shubina M, Turchin A. Encounter frequency and serum glucose level, blood pressure, and cholesterol level control in patients with diabetes mellitus. *Arch Intern Med.* Sep 26 2011;171(17):1542-1550.
109. Fleming TR. Addressing missing data in clinical trials. *Ann Intern Med.* Jan 18 2011;154(2):113-117.
110. Carter BL, Doucette WR, Franciscus CL, Ardery G, Kluesner KM, Chrischilles EA. Deterioration of blood pressure control after discontinuation of a physician-pharmacist collaborative intervention. *Pharmacotherapy.* Mar 2010;30(3):228-235.
111. Wentzlaff DM, Carter BL, Ardery G, et al. Sustained blood pressure control following discontinuation of a pharmacist intervention. *J Clin Hypertens (Greenwich).* Jun 2011;13(6):431-437.
112. Taveira TH, Wu WC, Martin OJ, Schleinitz MD, Friedmann P, Sharma SC. Pharmacist-led cardiac risk reduction model. *Prev Cardiol.* Fall 2006;9(4):202-208.
113. Bove AA, Santamore WP, Homko C, et al. Reducing cardiovascular disease risk in medically underserved urban and rural communities. *Am Heart J.* Feb 2011;161(2):351-359.
114. Chiu CC, Wu SS, Lee PY, Huang YC, Tan TY, Chang KC. Control of modifiable risk factors in ischemic stroke outpatients by pharmacist intervention: an equal allocation stratified randomized study. *J Clin Pharm Ther.* Oct 2008;33(5):529-535.
115. Ma J, Berra K, Haskell WL, et al. Case management to reduce risk of cardiovascular disease in a county health care system. *Arch Intern Med.* Nov 23 2009;169(21):1988-1995.
116. Lee JK, Grace KA, Taylor AJ. Effect of a pharmacy care program on medication adherence and persistence, blood pressure, and low-density lipoprotein cholesterol: a randomized controlled trial. *Jama.* Dec 6 2006;296(21):2563-2571.
117. Ellis SL, Carter BL, Malone DC, et al. Clinical and economic impact of ambulatory care clinical pharmacists in management of dyslipidemia in older adults: the IMPROVE study. Impact of Managed Pharmaceutical Care on Resource Utilization and Outcomes in Veterans Affairs Medical Centers. *Pharmacotherapy.* Dec 2000;20(12):1508-1516.
118. Kulchaitanaroaj P, Brooks JM, Ardery G, Newman D, Carter BL. Incremental costs associated with physician and pharmacist collaboration to improve blood pressure control. Pharmacotherapy 2012;32(8):772-780.
