# Supplementary material for: Selection bias and subject refusal in a cluster-randomized controlled trial
Source: BMC Med Res Methodol. 2017 Jul 10;17:94. doi: 10.1186/s12874-017-0368-7 (PMC5504663; doi:10.1186/s12874-017-0368-7)
Supplement: Supplementary file 3 [file 12874_2017_368_MOESM3_ESM.docx]

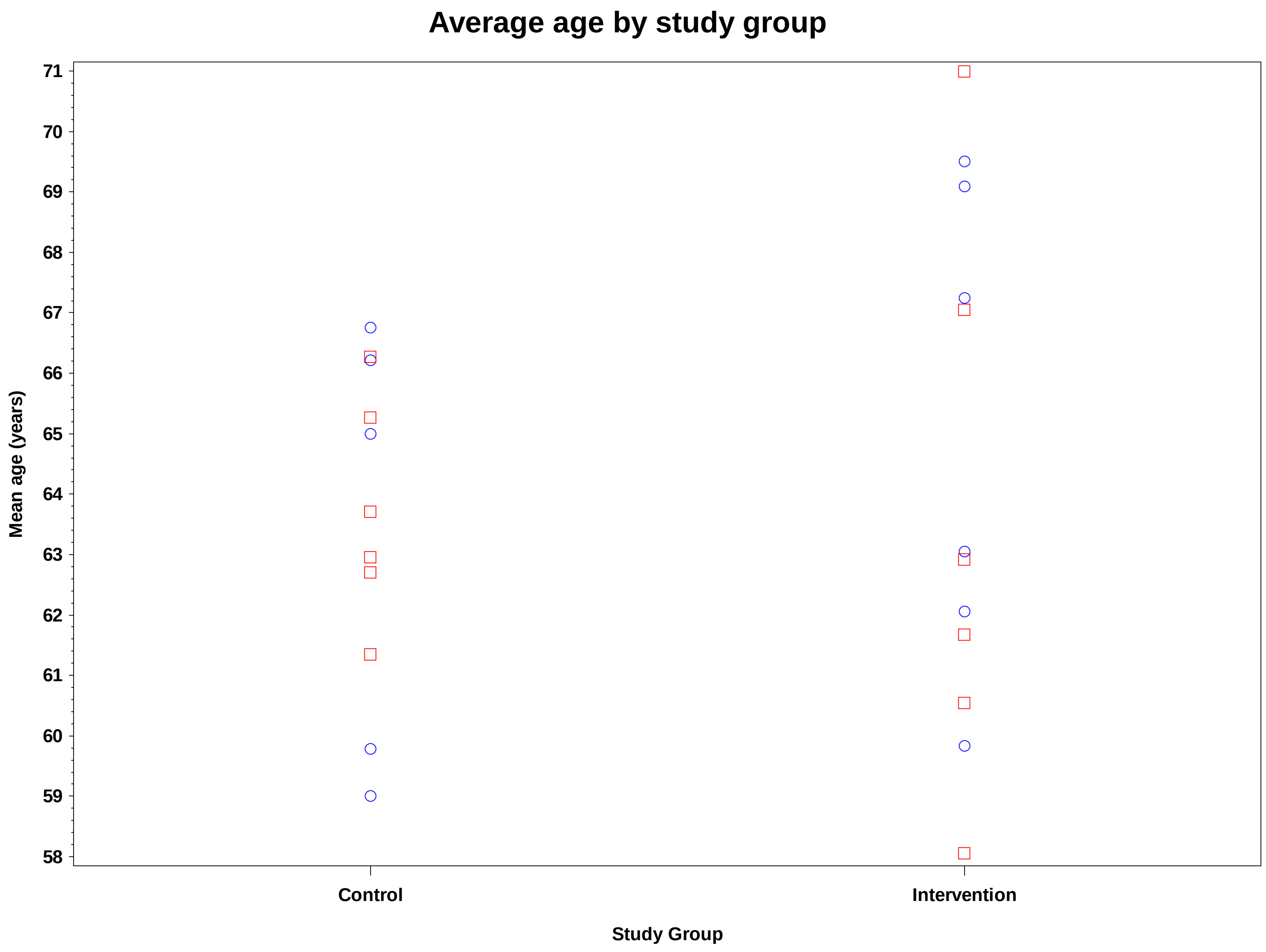
Average age by study group
Mean age (years)
71
70
69
68
67
66
65
64
63
62
61
60
59
58
Control
Intervention
Study Group

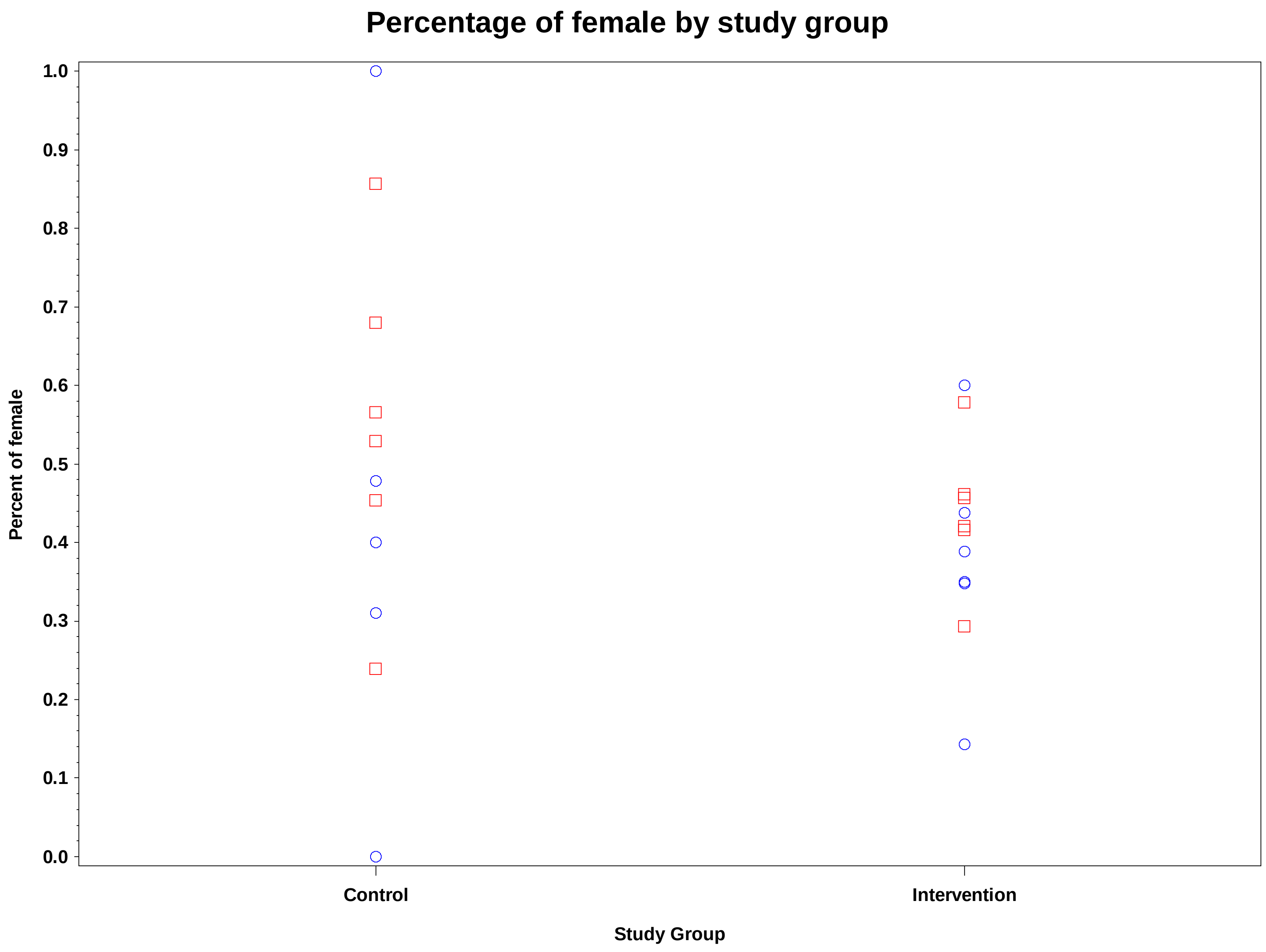

Percentage of female by study group
Percent of female
1.0
0.9
0.8
0.7
0.6
0.5
0.4
0.3
0.2
0.1
0.0
Control
Intervention
Study Group

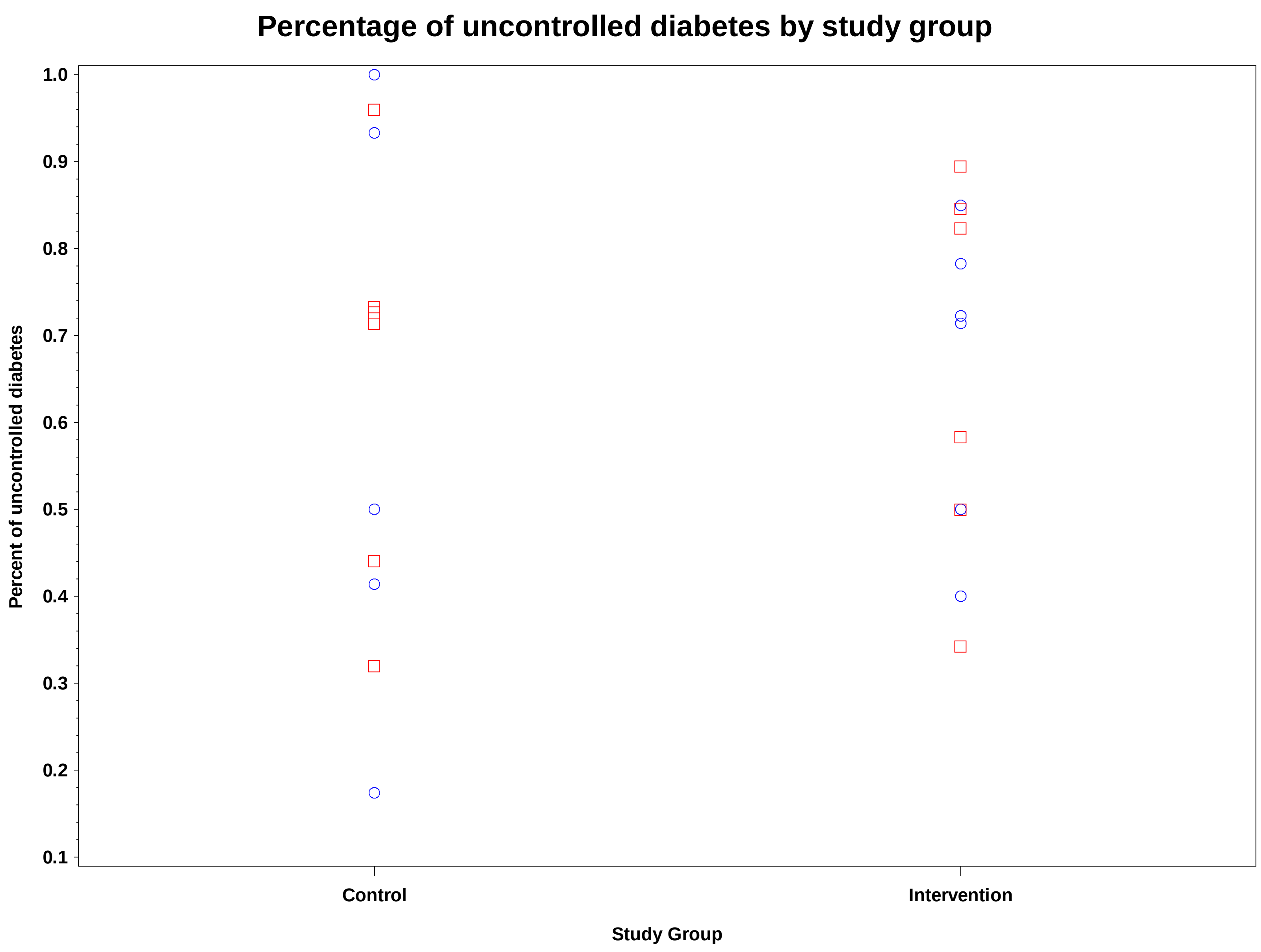

Percentage of uncontrolled diabetes by study group
Percent of uncontrolled diabetes
1.0
0.9
0.8
0.7
0.6
0.5
0.4
0.3
0.2
0.1
Control
Intervention
Study Group

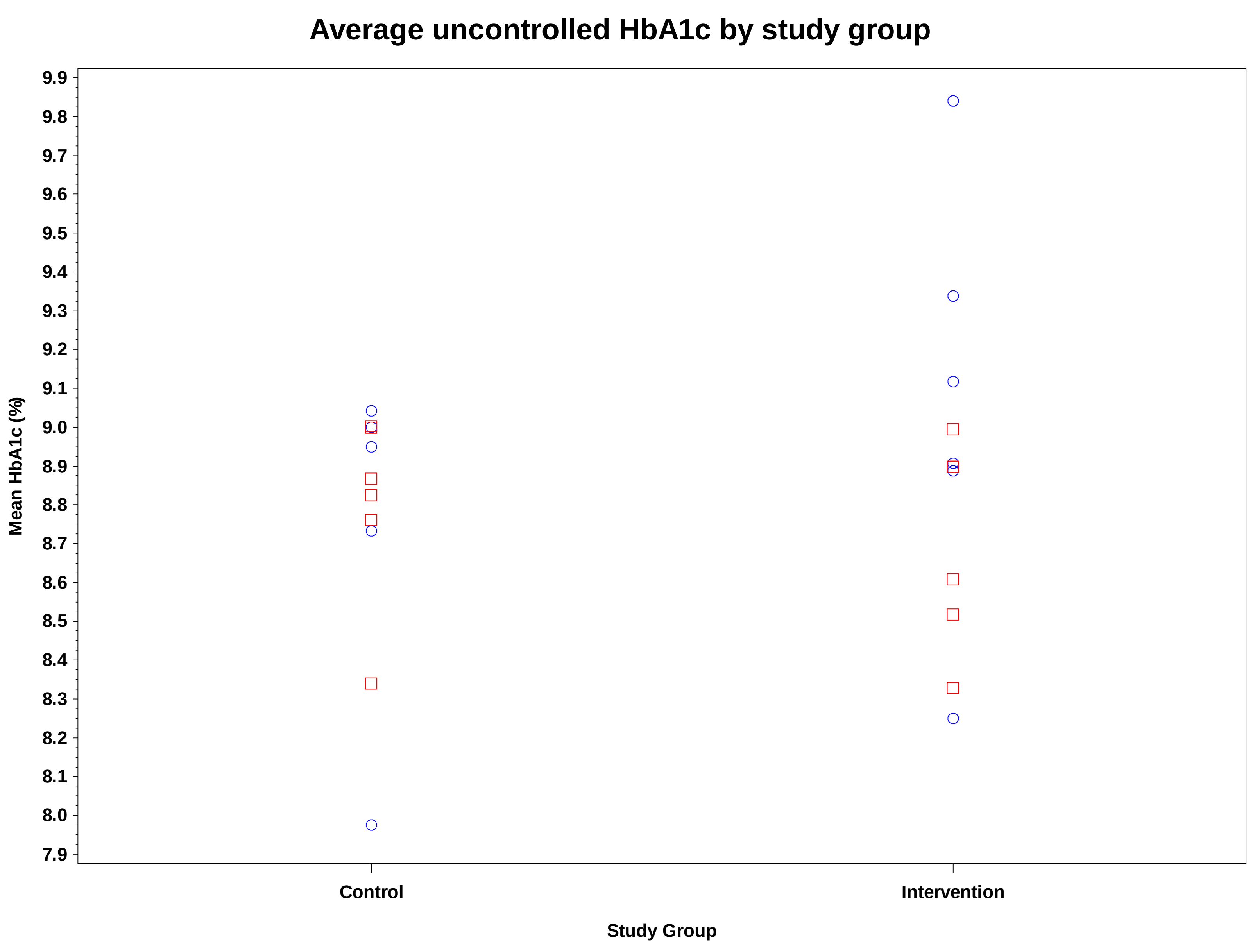

Average uncontrolled HbA1c by study group
Mean HbA1c (%)
9.9
9.8
9.7
9.6
9.5
9.4
9.3
9.2
9.1
9.0
8.9
8.8
8.7
8.6
8.5
8.4
8.3
8.2
8.1
8.0
7.9
Control
Intervention
Study Group

Percentage of uncontrolled hyperlipidemia by study group

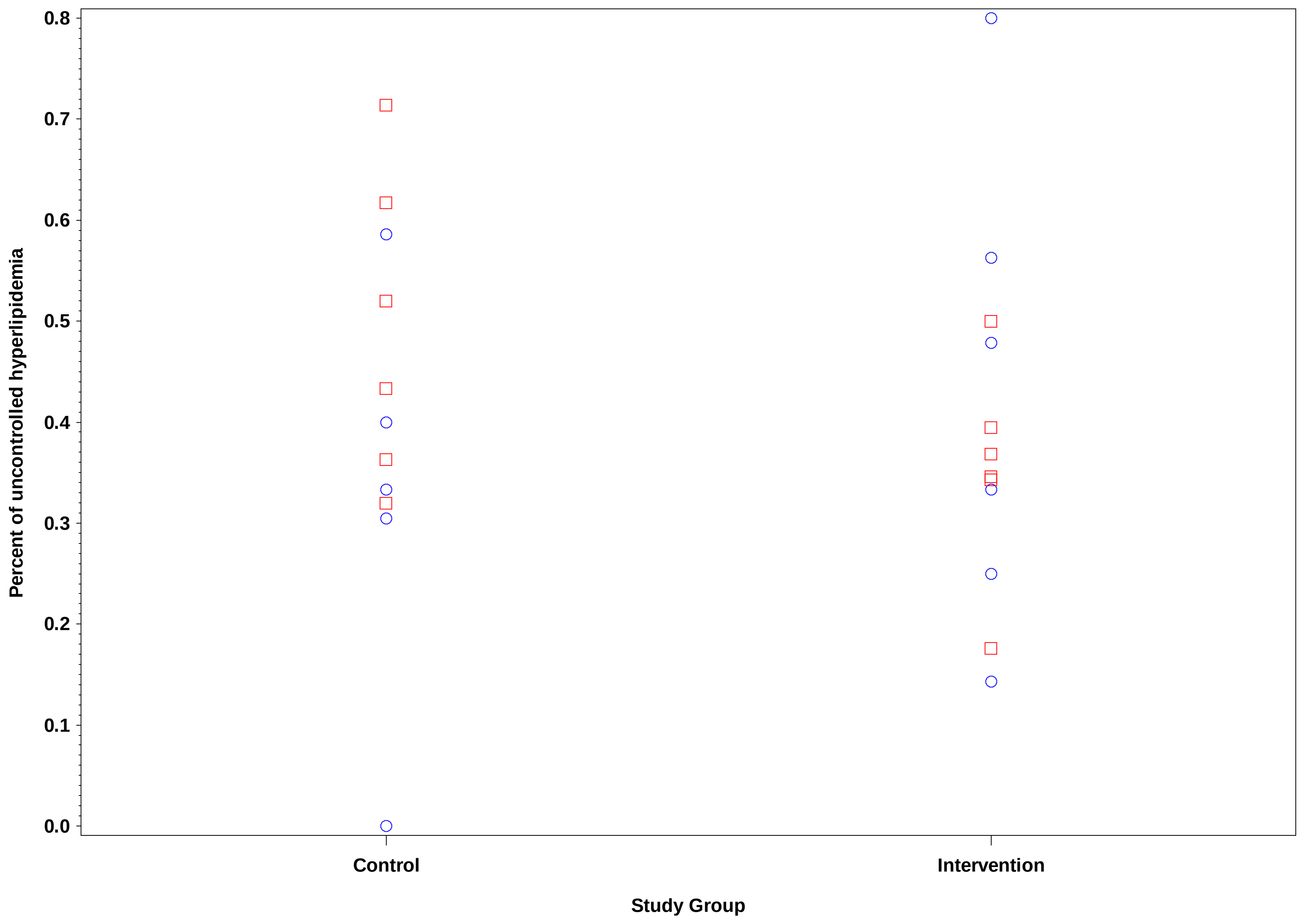

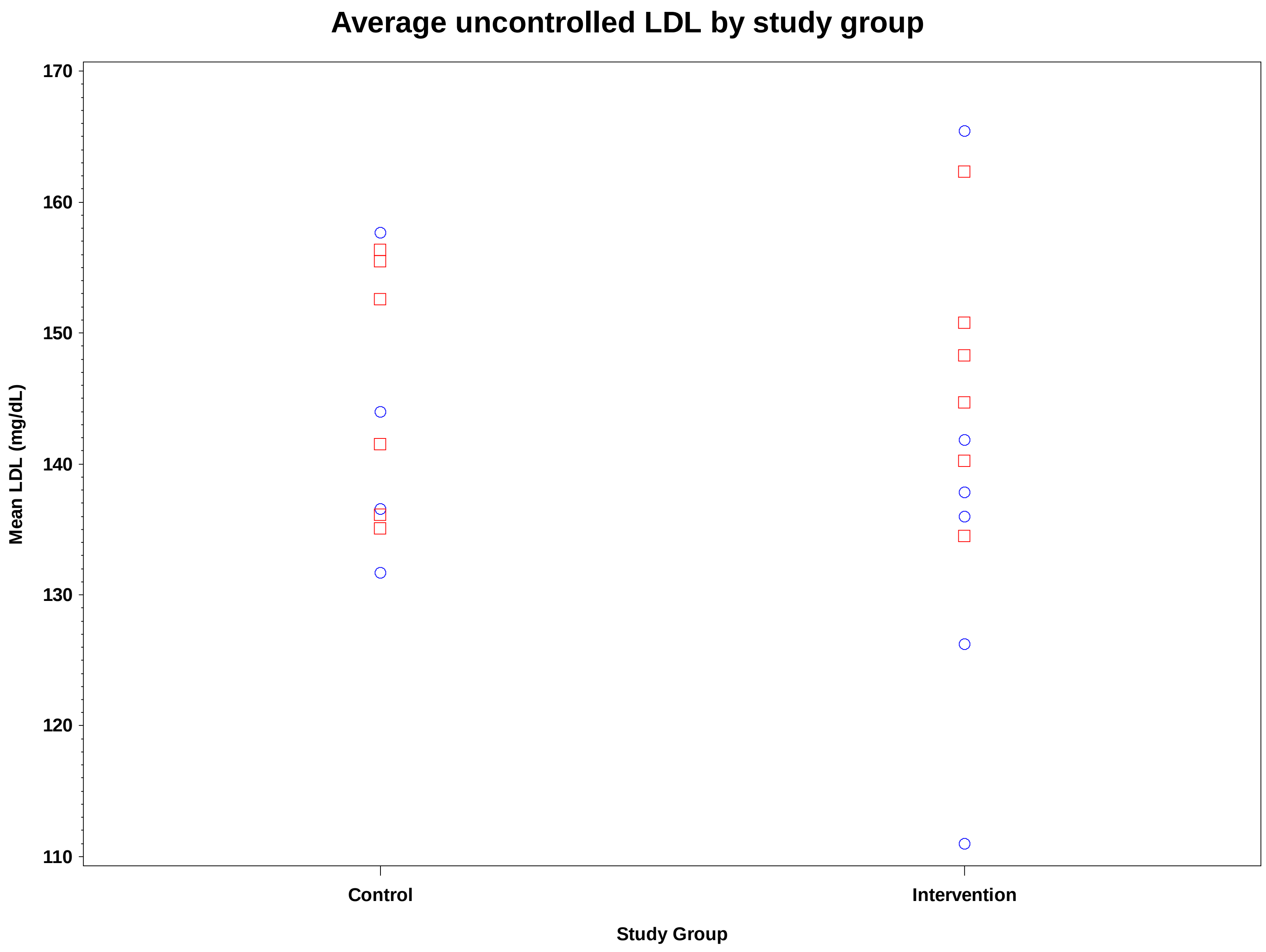
Average uncontrolled LDL by study group
Mean LDL (mg/dL)
170
160
150
140
130
120
110
Control
Intervention
Study Group

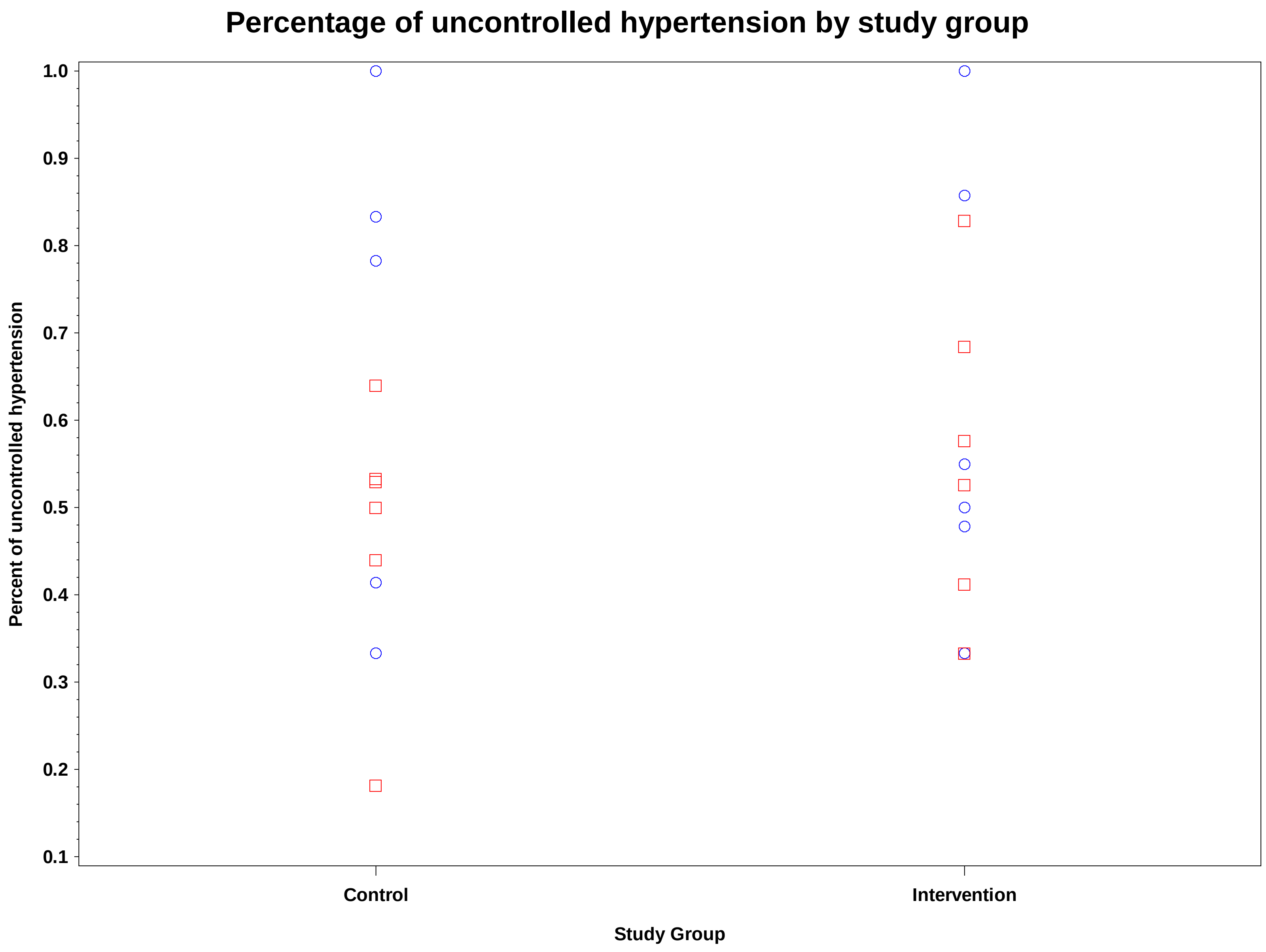
Percentage of uncontrolled hypertension by study group
Percent of uncontrolled hypertension
1.0
0.9
0.8
0.7
0.6
0.5
0.4
0.3
0.2
0.1
Control
Intervention
Study Group

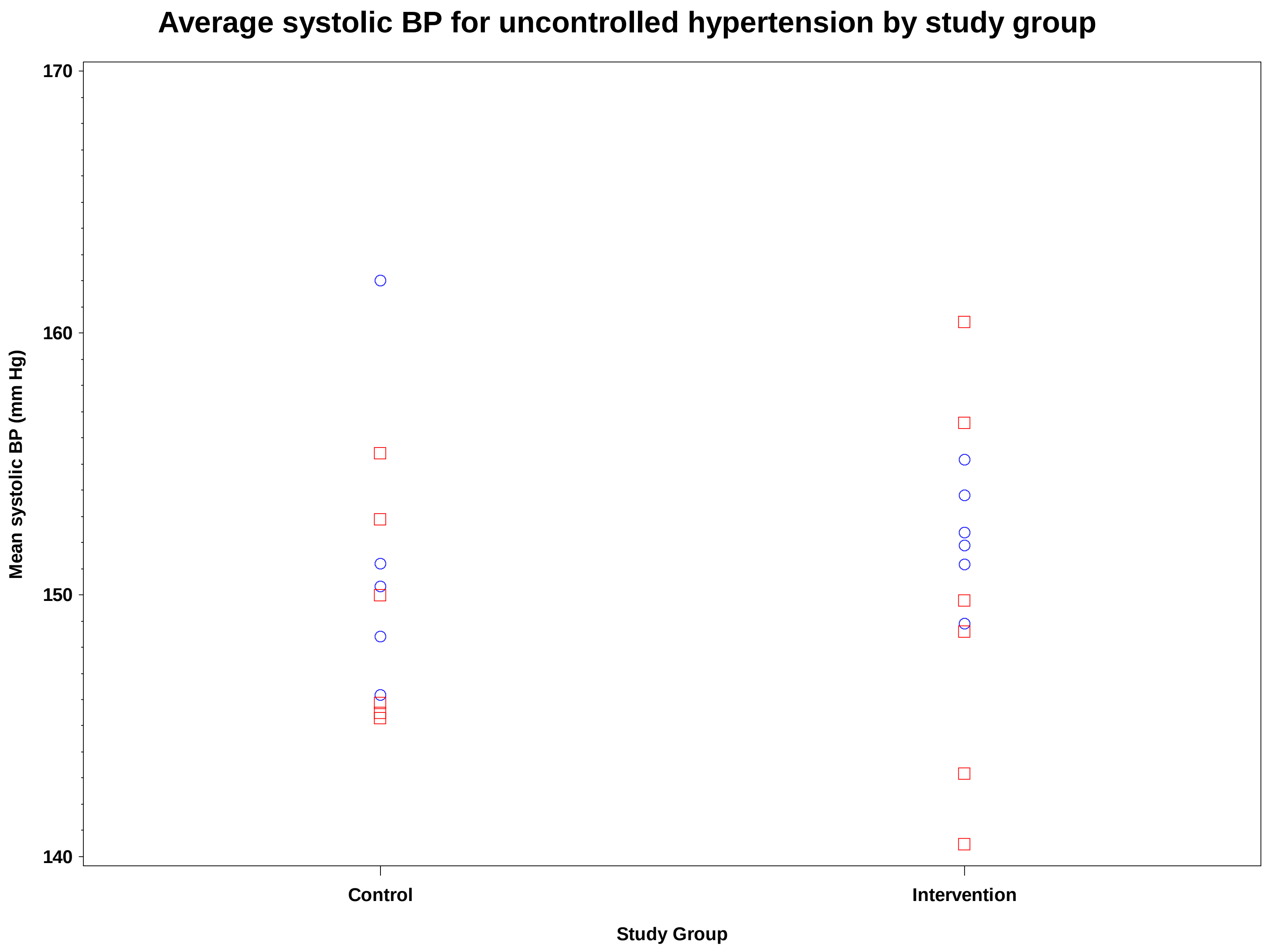

Average systolic BP for uncontrolled hypertension by study group
170
160
150
140
Mean systolic BP (mm Hg)
Control
Intervention
Study Group

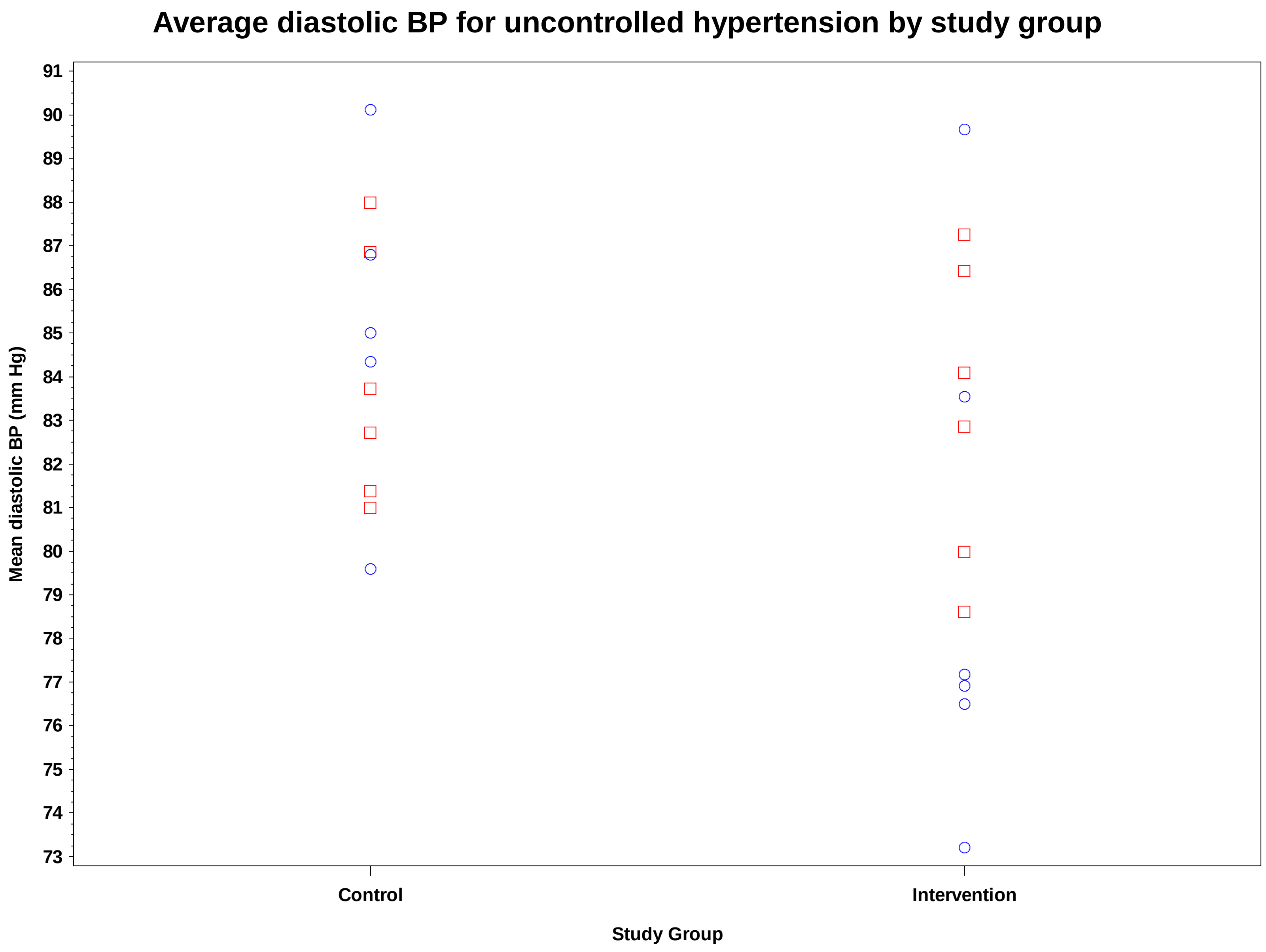
Average diastolic BP for uncontrolled hypertension by study group
Mean diastolic BP (mm Hg)
91
90
89
88
87
86
85
84
83
82
81
80
79
78
77
76
75
74
73
Control
Intervention
Study Group

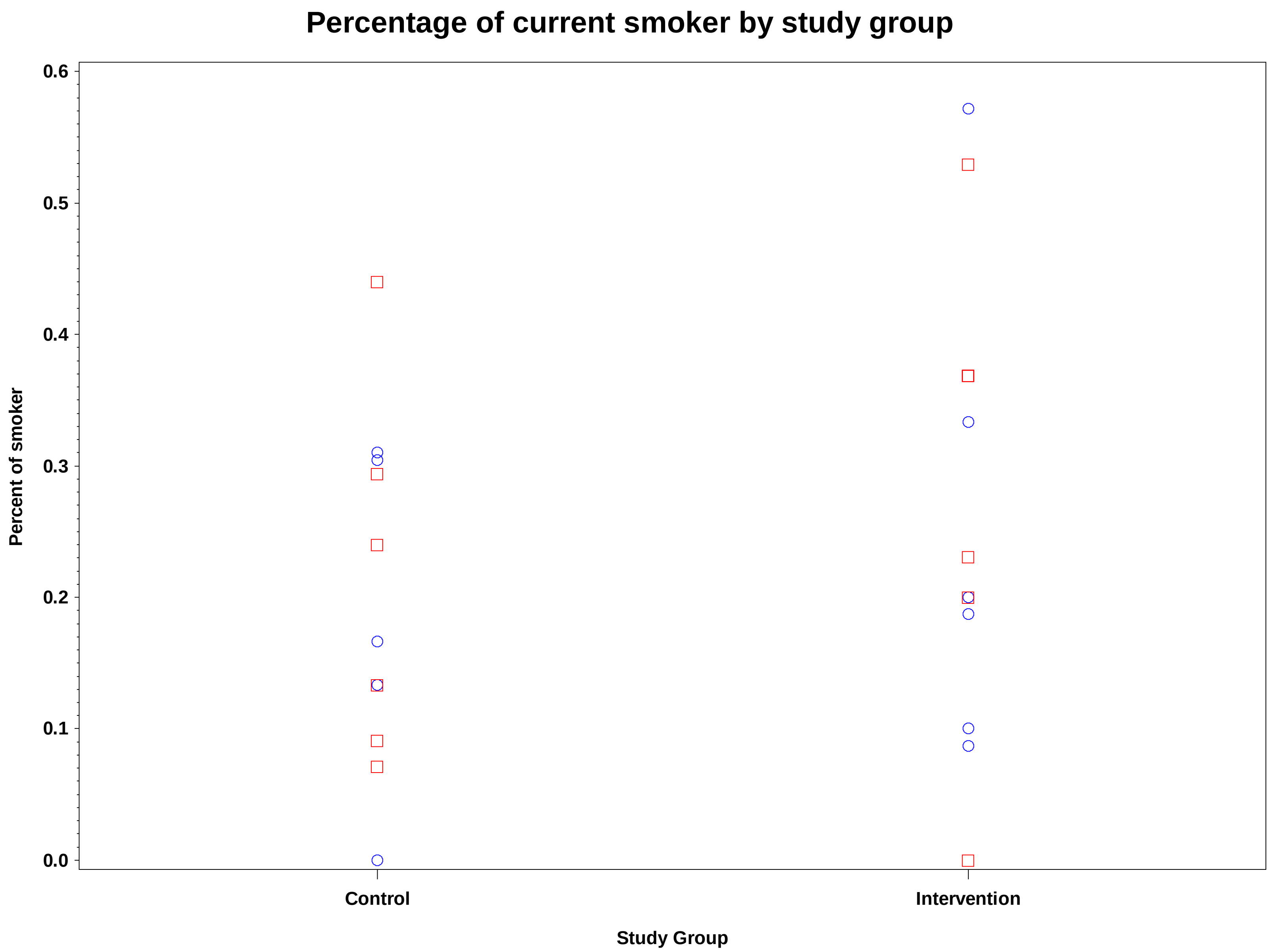

Percentage of current smoker by study group
Percent of smoker
0.0
0.1
0.2
0.3
0.4
0.5
0.6
Control
Intervention
Study Group
